# CCAFE: Estimating case and control allele frequencies from GWAS summary statistics

**DOI:** 10.1016/j.xhgg.2026.100616

**Published:** 2026-04-20

**Authors:** Hayley R. Stoneman, Hugo Lemus Gomez, Adelle Price, Christopher R. Gignoux, Audrey E. Hendricks

**Affiliations:** 1 Department of Biomedical Informatics, University of Colorado Anschutz Medical Campus, Aurora, CO 80045, USA; 2 Human Medical Genetics and Genomics Program, University of Colorado Anschutz Medical Campus, Aurora, CO 80045, USA; 3 Department of Mathematical and Statistical Sciences, University of Colorado Denver, Denver, CO 80204, USA; 4 Colorado Center for Personalized Medicine, University of Colorado Anschutz Medical Campus, Aurora, CO 80045, USA

**Keywords:** summary statistics, statistical genetics, R package, allele frequencies, GWAS

## Abstract

Genetic summary statistics can be used in a variety of analyses, such as causal inference, genetic correlation, and risk scores, to provide insights into the genetic architecture of conditions and traits. However, complete statistics are often not reported, limiting the utility of these data. Indeed, many post hoc analyses of diseases require case and control allele frequencies (AFs), which are not always published. Here, we present methods and software to derive case and control AFs from genome-wide association study (GWAS) summary statistics using the odds ratio, case and control sample sizes, and either the total (case and control aggregated) AF or the standard error (SE). In simulations and real data, derivations of case and control AFs using total AFs are highly accurate, whereas using SE underestimates AFs when covariates were included in the GWAS. While estimating case and control AFs using the total AF is preferred due to its high accuracy, the SE is more commonly available. Thus, we developed a bias adjustment using gnomAD AFs as a proxy for true AFs, reducing bias when using the SE. The methods and software provided here expand the utility of publicly available genetic summary statistics and promote the reusability of genomic data. The R package Case-Control Allele Frequency Estimation (CCAFE) is freely available on Bioconductor and GitHub.

## Teaser

We present methods and an accompanying software package, CCAFE, to derive case and control allele frequencies when missing from GWAS summary statistics. Our approach expands the utility and reusability of public genetic summary data.

## Introduction

Growth in genomics research has rapidly increased the number of genome-wide association studies (GWASs), which are often made publicly available through summary statistics to ease storage and privacy concerns. This increase in summary-level data has catalyzed our understanding of the relationship between genetics and health and disease through the development and application of methods such as GWAS meta-analysis,^1^ polygenic risk scores (PRSs),^2^^,^^3^ Mendelian randomization (MR),^4^ and external common controls.^5^^,^^6^^,^^7^ GWAS summary statistics often include a subset of odds ratios (ORs) or effect sizes (beta), standard errors (SEs), *p* values, allele frequencies (AFs), and sample sizes. However, there is a lack of standardization in reporting summary statistics, in both content and format, presenting challenges for data reuse.^8^^,^^9^^,^^10^ In 2021, a study of 327 previously curated, publicly available summary statistics files found over 100 unique formats^11^ resulting from different types of traits studied (i.e., binary or quantitative), the software used for analysis, or simply the summary statistics that the author chose to include in the final summary file. While movements have been made to standardize summary statistics reporting,^8^^,^^12^ inconsistency in reporting, including missing case and control AFs, can limit and sometimes fully hinder the use of these data for further studies.

In an assessment of the 2021 requirements for submission to the GWAS catalog, Hayhurst et al. found that over 50% of studies submitted between January 2020 and July 2022 were missing at least one mandatory field.^13^ Notably, the most commonly missing fields were effect AFs (>40% missing) and SEs (>25% missing)—although SEs can be recapitulated from the effect estimate and *p* value, which are more commonly included (0% and <20% missing, respectively). Inclusion of both case and control AFs was even more rare. Further, clear standards for reporting the effect AFs do not exist, with researchers frequently reporting the total sample AF, resulting in the aggregation of case and control samples. Without access to case- and control-specific AFs, secondary uses for summary data such as case-case GWASs, group PRSs,^14^ and external controls may not be possible. The implementation of standards for the content and format of GWAS summary statistics has the potential to greatly increase the number of usable datasets for downstream analyses in the future. However, the close to 90,000 available studies in the GWAS catalog as of March 2025 are not likely to be updated, limiting the utility of a vast amount of existing genetic summary data.

In 2022, Yang et al. presented a framework to reconstruct GWAS case and control AFs using case and control sample sizes, ORs, and SEs as part of a meta-analysis software for the reconstruction of allelic and genotypic frequencies and counts (ReACt).^14^ While Yang et al. used the derived case and control AFs in secondary analyses, such as case-case GWAS and meta-analysis, there was no evaluation of the derived case-control AFs and thus no understanding of the method’s accuracy or performance to estimate case and control AFs. To address this gap, we performed a rigorous evaluation of this SE-based case and control AF derivation in simulation and real data. Upon finding biased performance, we further developed a bias adjustment for Yang et al.’s method. Additionally, estimation of case and control AFs is embedded within the ReACt software and is not available as an independent function, further limiting use. We provide a user-friendly, stand-alone function that implements this method (and our subsequent bias adjustment) within the Case-Control Allele Frequency Estimation (CCAFE) software.

As another option for estimating case and control AFs, we developed a computationally efficient method that uses the total AF (instead of the SE). In real data and simulations, we evaluated and compared this AF-based method with Yang et al.’s SE method with and without our bias adjustment.

We provide functions for all methods in the *CCAFE R* software package,^15^ available on GitHub and Bioconductor.^16^ CCAFE enables derivation of GWAS case and control AFs using the GWAS total sample AF or SE, number of cases and controls, and effect estimates, supporting broader use of GWAS summary statistics. These well-documented and user-friendly functions will help to expand the use of summary statistics, enabling novel applications of existing data.

## Material and methods

### Implementation

#### CaseControl_AF mathematical framework

We derive case and control AFs for a given variant *i* using the case and control sample sizes (*n*_*case*_ and *n*_*control*_), *OR*_*i*_, and total AF (*AF*_*total*,*i*_). *AF*_*total*,*i*_ and *OR*_*i*_ can be represented as shown in Equations 1 and 2.

(Equation 1) $$AF_{total,i}=\frac{\left(n_{case}AF_{case,i}+n_{control}AF_{control,i}\right)}{n_{total}}$$

(Equation 2) $${OR}_{i}=\frac{a_{i}d_{i}}{b_{i}c_{i}}$$

Here, *a*, *b*, *c*, and *d* are the cells of a two-by-two contingency table representing the allele counts of the effect and non-effect, or alternate and reference, alleles for cases and controls. These quantities can be calculated as shown in Equations 3, 4, 5, and 6.

(Equation 3) $$a_{i}=2n_{case}*AF_{case,i}$$

(Equation 4) $$b_{i}=2n_{case}\left(1-AF_{case,i}\right)$$

(Equation 5) $$c_{i}=2n_{control}*AF_{control,i}$$

(Equation 6) $$d_{i}=2n_{control}\left(1-AF_{control,i}\right)$$

Substituting Equations 3, 4, 5, and 6 into the OR equation in Equation 2, we arrive at Equation 7.

(Equation 7) $${OR}_{i}=\frac{AF_{case,i}\left(1-AF_{control,i}\right)}{\left(1-AF_{case,i}\right)AF_{control,i}}$$

We can then use Equation 1 to solve for *AF*_*case*,*i*_ (shown in Equation 8) and substitute it into Equation 7, which results in a quadratic as shown in Equation 9.

(Equation 8) $$AF_{case,i}=\frac{n_{total}}{n_{case}}AF_{total,i}-\frac{n_{control}}{n_{case}}AF_{control,i}$$

(Equation 9) $$AF_{control,i}^{2}\left[\frac{n_{control}}{n_{case}}\left({OR}_{i}-1\right)\right]+AF_{control,i}\left[{OR}_{i}\left(1-\left(\frac{n_{total}}{n_{case}}AF_{total,i}\right)\right)+\frac{1}{n_{case}}\left(n_{control}+n_{total}AF_{total,i}\right)\right]-\frac{n_{total}}{n_{case}}AF_{total,i}=0$$

We solve for the quadratic roots and choose *AF*_*control*,*i*_ to be the root between 0 and 1. The full derivation is shown in Note S1. Additionally, we show there is only one solution for *AF*_*control*,*i*_ that falls between the bounds of 0 and 1 (Notes S2 and S3; Figure S1; Tables S1 and S2). *AF*_*case*,*i*_ is then estimated using Equation 8.

#### CaseControl_SE mathematical framework

The full derivation for the case and control AFs using SE can be found in the supplemental information of the original publication by Yang et al. Briefly, the method relies on allele counts *a*, *b*, *c*, and *d* as shown in Equations 3, 4, 5, and 6. Since this results in four unknown quantities, four equations are used to solve the system. These four unknown quantities are related to the SE, the sample size of cases and controls, and the OR. Ultimately, Yang et al. solve for *d* and use the resulting quadratic to solve for the allele counts and then the AFs. To fulfill the assumption that the solution is between [0, 1], Yang et al. use the minor AF (MAF), resulting in a loss of connection with an allele. While the minor and major alleles can likely be accurately inferred when the MAF is very different from 0.5, accurate inference of the minor allele becomes difficult as the MAF approaches 0.5. Notably, the resulting quadratic solved in this framework utilizes the total allele number (AN; i.e., 2∗*n* for autosomes) in cases and controls rather than the AF. This results in the need for sex-chromosome-specific implementations and thus requires information on the number of X and Y chromosomes in case and control samples.

#### CaseControl_SE bias correction

To reduce bias in the estimation of case and control AFs using the SE, we present a bias adjustment method. As the bias between the estimated AF and true AF was observed to be a curve, we tested a variety of appropriate models, finding the second-order polynomial to fit best. Because the bias trends with the MAF bin, we fit regression models by MAF bin. We evaluated using 5% vs. 10% MAF bins and observed nearly identical least squares (LS) difference. We therefore chose 10% MAF bins for computational efficiency. We recommend 10,000 variants per MAF bin in the proxy dataset for optimal bias correction (Note S4; Figures S2 and S3). For the implementation here, we fit a second-order polynomial where the outcome is the estimated MAF from the SE method and the predictor is the gnomAD MAF (Equation 10) for each MAF bin ([0, 0.1) [0.1, 0.2) [0.2, 0.3) [0.3, 0.4) [0.4, 0.5]) (Figure S4). We identified and excluded outliers defined as observations with an absolute value of the studentized residual >3 and refit the models.

The bias adjustment algorithm is shown below.

(Equation 10) $${\hat{MAF}}_{CaseControl\_SE}=\hat{a}*MAF_{gnomAD}^{2}+\hat{b}*{MAF}_{gnomAD}+\hat{c}$$

The bias can then be estimated as

(Equation 11) $$\hat{bias}={MAF}_{gnomAD}-{\hat{MAF}}_{CaseControl_{SE}}\text{.}$$

To complete the bias adjustment for variants not in the proxy data, the following steps are used.

(1) Polynomial regression (second order) is fit using the proxy data (*MAF*_*gnomAD*_) as the predictor and the CaseControl_SE MAF (${MAF}_{CaseControl\_SE}$) as the outcome by gnomAD MAF bin, resulting in estimates for $\hat{a},\hat{b},\text{and}\;\hat{c}$.
(2) For variants not in the proxy dataset (i.e., CaseControl_SE MAF, but unknown gnomAD MAF), solve for the estimated gnomAD MAF (${\hat{MAF}}_{gnomAD}$) using $\hat{a},\hat{b},\text{and}\;\hat{c}$.
  (Equation 12) $$0=\hat{a}*MAF_{gnomAD}^{2}+\hat{b}*{MAF}_{gnomAD}+\left(\hat{c}-{MAF}_{CaseControl\_SE}\right)$$
(3) Calculate ${\hat{MAF}}_{CaseControl\_SE}$.
  (Equation 13) $${\hat{MAF}}_{CaseControl\_SE}=\hat{a}*{\hat{MAF}}_{gnomAD}^{2}+\hat{b}*{\hat{MAF}}_{gnomAD}+\hat{c}$$
(4) Calculate $\hat{bias}$.
  (Equation 14) $$\hat{bias}={\hat{MAF}}_{gnomAD}-{\hat{MAF}}_{CaseControl\_SE}$$
(5) Calculate ${MAF}_{CaseControl\_SE}^{*}$.
  (Equation 15) $${MAF}_{CaseControl\_SE}^{*}={\hat{MAF}}_{CaseControl\_SE}+\hat{bias}$$

#### Implementation in R

We include the method from Yang et al. as a function, named CaseControl_SE, in our R package. As this method was originally embedded in a GWAS meta-analysis software developed in C, we replicated the results using their original software in C and our version in R to ensure complete and robust translation of the code (Note S5). We also include our framework as the function CaseControl_AF. Both methods (using either total AF or SE) rely on solving the roots of a quadratic. As we know that these quantities (AFs) exist and are real numbers, this can be done easily in R in closed form, ensuring scalability for large datasets. Both methods use the number of cases and controls, ORs, and either the total AF or SE as inputs. Note that the OR can be derived from the beta estimate, and the SE can be derived given the effect estimate and either the *p* value or the test statistic. To use the SE to derive the MAF for sex chromosomes, the user must also include the number of X and Y chromosomes per case and control sample.

Additionally, the user may obtain bias-corrected MAF estimates from CaseControl_SE by including a data frame with variants from the observed data and a proxy dataset, such as gnomAD. This data frame must contain variant information (chromosome and position) and the proxy MAF (e.g., from gnomAD) and be merged so that the AF matches the same allele. As the bias correction can be estimated by a subset of variants, this data frame does not need to contain all variants in the observed dataset, which helps ensure more efficient computation. The adjusted MAFs (total, case, and control) are appended as three additional columns to the original data input.

### Simulation study

We used the R package *PhenotypeSimulator*^17^ to simulate models with genotypes for 10,000 variants for one binary phenotype and zero, one, or three covariates (Figure S5). *PhenotypeSimulator* generates phenotypes as the sum of genetic variant effects, covariate effects, and observational noise. We simulated multiple sample sizes (*n* = 1,000, 6,000, 10,000, 50,000, and 100,000) with equal numbers of cases and controls, as well as two imbalanced sample sizes (*n* = 6,000: 600 cases and 5,400 controls emulating the Pan-UK Biobank (Pan-UKBB) African Diabetes sample; *n* = 50,000: 1,200 cases and 48,800 controls). When including one covariate, a binary variable, such as sex, was simulated with a probability of 0.5. For the three covariate simulation scenarios, two additional variables were simulated: one categorical variable, such as educational attainment or geographical region (five categories), and a continuous variable, e.g., similar to age.

The binary phenotype was simulated with *PhenotypeSimulator* using a binomial distribution with the probability determined by the proportion of variance explained for causal variants (genVar). The additional parameters of the simulation model split the remaining variance (i.e., 1 – genVar) proportionally: kinship (eta), correlated covariates (rho), uncorrelated covariates (delta), and random noise (phi). Non-causal variants have an effect size of zero by default. For our simulations, we simulated the proportion variance explained by causal variants (genVar) as 0.8, leaving 0.2 leftover, which was split 0.6 for uncorrelated covariates (delta) and 0.4 for random noise (phi). In other words, 0.6 × 0.2 = 0.12 and 0.4 × 0.2 = 0.08 of the variance was explained by uncorrelated covariates and random noise, respectively. In the initial simulations, we did not simulate correlated covariates or relatedness (i.e., eta = 0). We simulated 100 causal variants and 9,900 non-causal variants. The 9,900 non-causal variants were simulated using the same framework, with the proportion of variance explained only by covariates. More information can be found in the *PhenotypeSimulator* publication^17^ and the CRAN manual (https://cran.r-project.org/web/packages/PhenotypeSimulator/index.html).

The simulated genotype, phenotype, and covariate data were then used to fit a logistic regression model from which the OR and SE for each genetic variant were obtained. Using the logistic regression summary statistics (e.g., OR and SE) in CCAFE, the case and control AFs were estimated and compared to the true calculated values from the case and control samples assessed using Lin’s concordance correlation coefficient (CCC)^18^ (additional details are provided in data and code availability). Lin’s CCC measures the concordance between two measures of the same variable (here, the estimated AF and true AF), with perfect agreement at 1. To assess the bias correction framework, we simulated proxy MAFs by adding a small amount of random noise (uniform distribution from −0.075 to 0.075) to the simulated total MAFs for both non-causal and causal variants.

To evaluate the methods in the presence of population stratification, we simulated population-specific AFs for two groups using gnomAD African (AFR-like) and European (EUR-like) AFs. We combined the AFs, weighting 80% AFR-like and 20% EUR-like. For a random subset of 10% (*N* = 1,000) of variants, we only used the EUR-like AF. We simulated the two reference groups to have different case probabilities (1.5× and 1× for AFR-like and EUR-like, respectively), thus creating confounding due to population stratification. Simulation of the phenotype and three covariates proceeded as previously described. The first 10 principal components (PCs) were estimated using the *stats* R package and were used for the adjusted regressions. We also tested the effect of relatedness by including kinship in *PhenotypeSimulator* (eta = 0.6), which allows part of the genetic variance to be explained by relatedness between individuals rather than causal SNPs.

Genotype dosage can help account for uncertainty in genotype calling. Thus, we evaluated a scenario in which the OR may be estimated from genotype dosage while the AF is estimated from genotype calls. We simulated AC for bi-allelic variants (i.e., 0, 1, or 2) for 10,000 variants in 10,000 individuals using *rmultinom* in R with a *p* equal to the AF. We added a small (±0.1) or moderate (±0.2) amount of random noise using a uniform distribution to each genotype. We then assessed concordance in AFs calculated using the true genotypes without added uncertainty, genotype dosages with added uncertainty, and genotype calls obtained by rounding genotype dosages.

### Real data application

#### Publicly available datasets

We tested the ability of the methods to reconstruct case and control AFs from real datasets for which case and control AFs were publicly available (Table 1). These datasets had a range of case and control sample sizes, numbers of genetic variants, and covariates included in the original GWAS. We used 148 variants from a 2018 prostate cancer PGS (https://www.ebi.ac.uk/gwas/studies/GCST006085), which was generated from data collected for a GWAS of 79,148 cases and 61,106 controls.^19^ This GWAS published control AFs, which were used with the OR to derive the case AFs (Note S6). Additionally, we use the Pan-UKBB GWAS summary statistics for diabetes in EUR and AFR samples (https://pan-dev.ukbb.broadinstitute.org/docs/per-phenotype-files/index.html).^20^ Here 9,178,564 genome-wide variants were available. The Pan-UKBB EUR GWAS contained 16,550 cases and 403,923 controls, while the Pan-UKBB AFR GWAS contained 668 cases and 5,956 controls. We also used the Pan-UKBB EUR GWAS data to evaluate the effect of summary statistic rounding. As the AF usually has more significant digits reported, we evaluated Lin’s CCC with rounding from nine to two decimal places for AFs, whereas we evaluated five to two decimal places for SEs. We compared the estimated case and control AFs to the known AFs using Lin’s CCC per MAF bin.

**Table 1:** Lin’s concordance correlation coefficient in simulations

Cases	Controls	Total	Covariates	Lin’s CCC			
				Cases		Controls	
				CCAFE AF	CCAFE SE	CCAFE AF	CCAFE SE
500	500	1,000	0	0.9916	0.9811	0.9917	0.9811
			1	0.9905	0.7430	0.9914	0.7420
			3	0.9916	0.6913	0.9905	0.6899
3,000	3,000	6,000	0	0.9982	0.9952	0.9983	0.9951
			1	0.9981	0.8457	0.9980	0.8442
			3	0.9981	0.6690	0.9982	0.6690
5,000	5,000	10,000	0	0.9988	0.9967	0.9988	0.9966
			1	0.9987	0.8607	0.9988	0.8599
			3	0.9986	0.6747	0.9986	0.6747
25,000	25,000	50,000	0	0.9995	0.9989	0.9995	0.9989
			1	0.9993	0.8641	0.9993	0.8643
			3	0.9994	0.6806	0.9993	0.6808
50,000	50,000	100,000	0	0.9995	0.9993	0.9995	0.9993
			1	0.9995	0.7589	0.9994	0.7593
			3	0.9995	0.6761	0.9995	0.6754
600	5,400	6,000	0	0.9921	0.9941	0.9980	0.9882
			1	0.9916	0.8410	0.9980	0.8369
			3	0.9918	0.8234	0.9980	0.8189
1,200	48,800	50,000	0	0.9956	0.9992	0.9994	0.9955
			1	0.9954	0.9903	0.9994	0.9868
			3	0.9954	0.9773	0.9994	0.9731

All simulations contained 10,000 variants (100 causal). AF and SE columns contain Lin’s CCC between estimated and true case and control AFs for CaseControl_AF and CaseControl_SE, respectively.

#### Correction using gnomAD as proxies

We applied our bias correction to the Pan-UKBB diabetes GWAS data (>9 million variants), filtering down to chromosome 1 bi-allelic variants from gnomAD as proxies (https://gnomad.broadinstitute.org/downloads). We lifted over Pan-UKBB from GRCh37 to GRCh38 using LiftOver and merged with gnomAD v.3.1.2,^21^ retaining the intersection of variants. We removed variants where the allele pair did not match between the two datasets. We flipped informative alleles (e.g., A/G and C/T) and the AF so that the reference and alternate alleles were the same between datasets. Non-informative allele pairs (i.e., A/T and C/G) were removed. The final dataset contained 1,212,618 variants on chromosome 1. We used the gnomAD non-Finnish European (NFE) group as a proxy for total MAFs in the Pan-UKBB EUR sample and the gnomAD AFR/African American (AFR/AFRAM) group as a proxy for MAFs in the Pan-UKBB AFR sample. We then adjusted the estimated MAFs for all >9 million genome-wide variants in each sample.

## Results

### Simulation results

**Figure 1:**
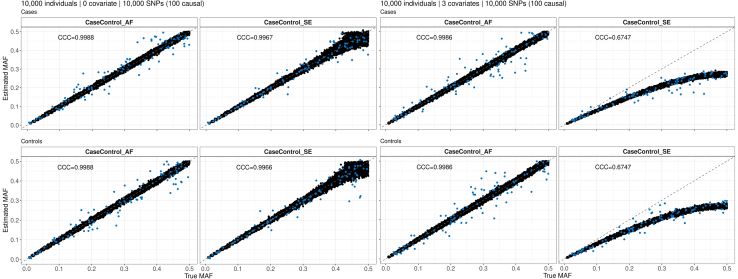
Estimated case and control AFs from summary statistics Simulated genotypes and phenotypes were generated using the *PhenotypeSimulator* R package. Genotypes for 10,000 variants, of which 100 were causal (shown in blue), were generated for 5,000 cases and 5,000 controls. Logistic regression was used along with 0 (A) or 3 (B) covariates to generate per-variant summary statistics. CaseControl_AF and CaseControl_SE methods were used to estimate the case and control AFs. When covariates were included in the original GWAS, bias increased as MAF increased for CaseControl_SE, resulting in a systematic underestimation of MAFs (B). CaseControl_AF was accurate across the MAF spectrum, regardless of whether covariates were included. Lin’s CCC is shown between the true simulated MAF and the estimated MAF.

For CaseControl_SE, the previously published method that uses SE rather than total AF, we found an increase in the variance in AF estimates at higher MAFs (i.e., MAF > 0.4) (Figures 1 and S6). When simulated covariates were included in the regression to produce the summary statistics, CaseControl_SE also had increasing bias (average difference between the estimated and true MAF) as the MAF increased, with the MAF systematically underestimated (Figures 1 and S7–S10). Conversely, CaseControl_AF, the method introduced here that uses total AF, provided accurate estimates with small variation across the MAF spectrum in all tested scenarios, even with the presence of covariates (Figure S6). This was reinforced by the Lin’s CCC between the true MAF and the estimated MAF, which decreased as the number of covariates increased for CaseControl_SE but remained near 1 for CaseControl_AF (Table 1). Notably for both methods, the estimates for some causal variants were variable compared to the estimates for the non-causal variants (Figures 1 and S6–S8). In addition, by evaluating AF-to-MAF transformations in the CaseControl_AF method (which can use either AF or MAF), we found that using the total MAF rather than the total AF consistently increased the variability of the estimates across the MAF spectrum (Figure S11).

We further assessed the effects of population stratification, relatedness, and genotype dosage. For both Control_AF and CaseControl_SE, we found that confounding due to population stratification (Figure S12) increased the variability of the AF estimates compared to simulations without confounding, whereas relatedness in the sample used to generate the summary statistics did not noticeably affect the results (Figure S13). Correcting for population stratification by including the first 10 PCs reduced the variability but not to the level seen without population stratification (Figure 1). While variability increased, the bias was not substantially affected in the presence of population stratification or relatedness. We found high concordance between the true AF, dosage AF, and genotype from dosage AF, even given the small and moderate uncertainty added to the latter two scenarios (Figure S14).

### Real data analysis

**Figure 2:**
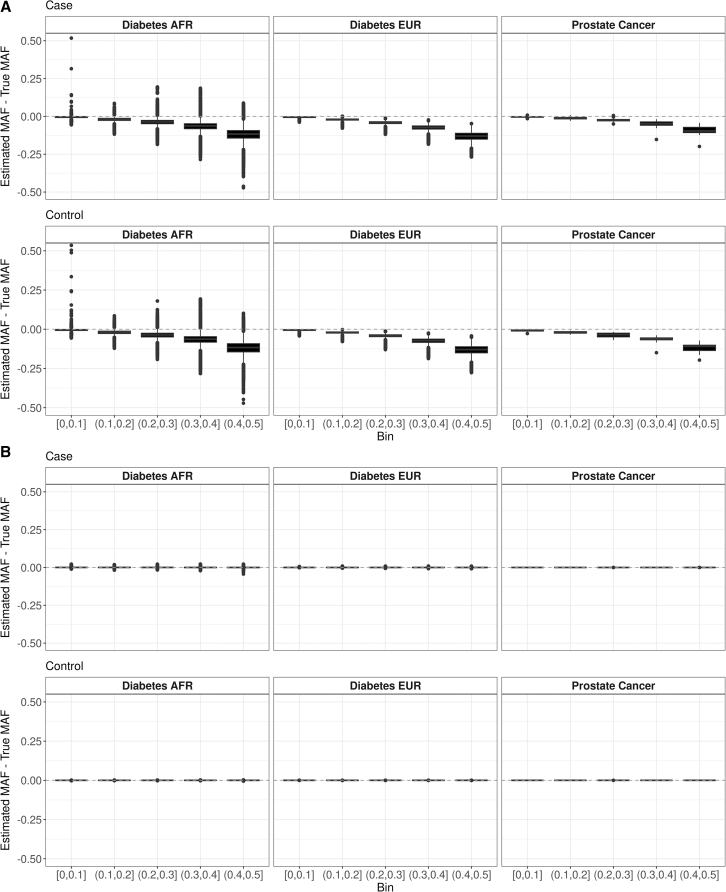
Comparison of case and control AF estimation in real datasets Results of estimating case MAFs for six datasets with various sample sizes. The prostate cancer dataset (*n*_*case*_ = 79,148; *n*_*control*_ = 61,106) has 148 variants from a 2018 PRS. Diabetes EUR (*n*_*case*_ = 16,550; *n*_*control*_ = 403,923) and Diabetes AFR (*n*_*case*_ = 668; *n*_*control*_ = 5,956) contain >9 million variants from the PanUKBB GWAS. The lower and upper hinges of boxes correspond to the 25^th^ and 75^th^ percentiles, respectively. Upper and lower whiskers are the largest and smallest values no further than 1.5× the inter-quartile range (IQR) from the hinge, and the center line represents the median. (A) CaseControl_SE, the method proposed in the ReACt software using SE, underestimates the true MAF, with bias increasing and precision (width of the boxplot) decreasing as the true MAF increases. (B) CaseControl_AF, the framework developed here using total AF produces a highly accurate estimation of known AFs using CaseControl_AF, with some variability in the Diabetes AFR dataset, which has a smaller sample size.

CaseControl_SE underestimated the true case and control AFs in all datasets, especially for variants with a higher MAF (Figure 2A). We identified the highest bias for variants with small SEs (i.e., SE < 0.02) (Figures S15 and S16). Variants with smaller SEs were often those with a large MAF (e.g., MAF > 0.3). Additionally, as expected, the minimum SE was smaller when case sample sizes were larger, resulting in a positive correlation between bias and sample size. When using the SE to reconstruct the case and control AFs, the CCC was lower for higher MAF bins (i.e., CCC < 0.06 for MAF [0.4, 0.5] vs. CCC > 0.90 for MAF [0, 0.1)), and this pattern was particularly compounded for datasets with larger numbers of cases (Tables S2 and S3). Conversely, CaseControl_AF accurately reconstructed the case and control AFs in all datasets (Figure 2B), and the CCC of the estimated case and control AFs with the true AFs remained at or near 1 (Tables S2 and S3). Rounding the SE to three or more decimal places produced no notable decrease in accuracy; however, rounding to two decimal places resulted in a decrease in accuracy for CaseControl_SE, especially for MAFs > 0.1 (Tables S4 and S5). While Lin’s CCC also decreased when rounding AF to two decimal places, overall concordance was still high (>0.99).

### CaseControl_SE bias correction

**Figure 3:**
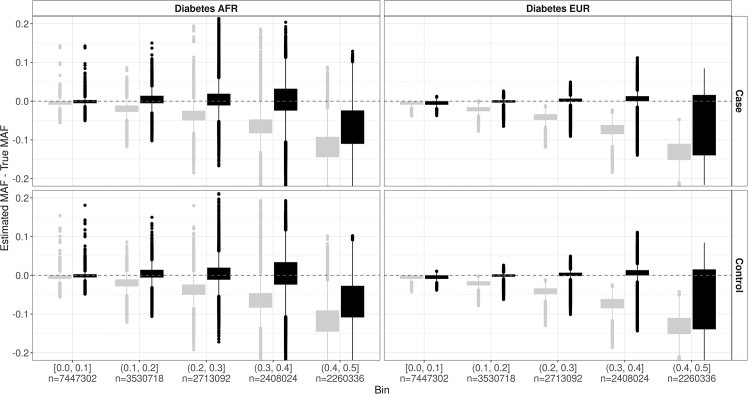
Correction mitigates bias in CaseControl_SE MAF estimates We use our bias correction to adjust the case and control MAF estimates from CaseControl_SE for >9 million genome-wide variants from the AFR and EUR Pan-UKBB Diabetes datasets. To estimate the bias correction, we used >1.2 million variants on chromosome 1 that were harmonized between Pan-UKBB and gnomAD v.3.1.2. The gnomAD African/African American (AFR/AFRAM) group was used as a proxy for true MAFs for the AFR sample (left) and the gnomAD non-Finnish European (NFE) group was used as a proxy for true MAFs for the EUR sample (right). We see an improvement (i.e., less bias and greater Lin’s CCC) when using the bias correction (gray; AFR CCC = 0.9877, EUR CCC = 0.9943) compared to the uncorrected CaseControl_SE MAF estimates (black; AFR CCC = 0.9369, EUR CCC = 0.9382). The lower and upper hinges of boxes correspond to the 25^th^ and 75^th^ percentiles, respectively. Upper and lower whiskers are the largest and smallest values no further than 1.5× the inter-quartile range (IQR) from the hinge, and the center line represents the median.

We developed a bias correction framework using gnomAD v.3.1.2 AFs as proxies for the true AFs. We tested our framework using the Pan-UKBB Diabetes datasets. The adjusted MAFs using the bias correction had less bias and a higher Lin’s CCC for both the AFR and EUR samples than the unadjusted method (Figure 3; Table 2). The average CCC across all variants improved from 0.9369 to 0.9877 for the PanUKBB AFR diabetes GWAS and from 0.9382 to 0.9943 for the EUR sample. While the overall bias is much lower with the bias correction, we still observe an increase in bias and variance as the MAF increases, especially for higher MAF bins (Figure 3). Similar results were found in simulations (Figure S17). Notably, we observe similar bias in the causal vs. non-causal variants for both the bias-corrected and uncorrected estimates.

**Table 2:** Concordance of inferred AF in real GWAS datasets for cases

Trait	Cases	Controls	MAF bin	*N* variants	Lin’s CCC		
					CCAFE AF	CCAFE SE	CCAFE SE corrected
Prostate cancer	79,148	61,106	[0.0, 0.1]	18	1	0.9643	N/A^a^
			(0.1, 0.2]	27	1	0.8560	N/A
			(0.2, 0.3]	35	1	0.6592	N/A
			(0.3, 0.4]	32	1	0.2401	N/A
			(0.4, 0.5]	36	1	0.0596	N/A
PanUKBB Diabetes EUR	16,550	403,923	[0.0, 0.1]	3,721,679	0.99998	0.9701	0.9651
			(0.1, 0.2]	1,766,962	0.99993	0.7346	0.9805
			(0.2, 0.3]	1,355,742	0.99989	0.3977	0.9404
			(0.3, 0.4]	1,203,911	0.99986	0.1425	0.8244
			(0.4, 0.5]	1,130,270	0.99985	0.0213	0.2022
PanUKBB Diabetes AFR	668	5,956	[0.0, 0.1]	3,453,818	0.99871	0.9624	0.9774
			(0.1, 0.2]	1,871,888	0.99640	0.7179	0.8891
			(0.2, 0.3]	1,487,374	0.99500	0.4168	0.7523
			(0.3, 0.4]	1,242,683	0.99409	0.1666	0.4580
			(0.4, 0.5]	1,126,168	0.99358	0.0278	0.2146

Lin’s concordance correlation coefficient for CaseControl_AF, CaseControl_SE, and bias-corrected CaseControl_SE between true and estimated MAFs in real GWAS datasets.

a Prostate cancer MAFs were not corrected due to the small number of overlapping variants with gnomAD.

## Discussion

Here, we present methods and software (CCAFE) to estimate case- and control-specific AFs from GWAS summary statistics. While our method using total AF outperforms the SE-based method and has lower bias and variability in its estimates, both methods allow flexibility based on what summary statististics are avaiable. This is especially important given the inconsistency in the summary statistics released in repositories such as the GWAS catalog, with the effect AF missing in >40% of them.^12^ Indeed, SE is often more available and easily derivable (e.g., given the effect estimate and either a *p* value or test statistic) than total AF. To increase robust use of the SE method, we developed and provided a bias adjustment within the R package that greatly decreases the bias in the case and control AF estimates, which can be especially large for higher variants with higher MAFs.

In both simulations and real data, we show that CaseControl_AF provides highly accurate estimates of case and control AFs across a variety of sample sizes and in the presence of covariates. Conversely, while CaseControl_SE provides accurate AF estimates when no covariates are included in the original GWAS, it is biased when the original GWAS includes covariates. Since CaseControl_AF is much less biased and more precise, we recommend using CaseControl_AF when total AFs are available. We hypothesize that the bias in CaseControl_SE arises from an underestimation of the SE used for the derivation in the ReACt method when covariates are included in the original GWAS. Specifically, the ReACT method derivation assumes a simple linear regression with no covariates. However, most publicly available GWAS summary statistics are from models that adjust for covariates. Adjusting for covariates usually reduces the model’s residual error and, consequently, the SE as well, resulting in a smaller SE than a simple regression without covariates.^22^ Using the MAF in CaseControl_SE also results in more variability, most likely due to a loss of information in allele polarity, which can result in errors when harmonizing datasets. To ensure the estimated AF is between zero and one, a key assumption of the SE method by Yang et al. is that the estimated allele is always the minor allele. As such, there is no connection between the reported alleles in the summary statistics and the estimated MAF. This loss of information regarding alleles can complicate secondary analyses, as accurately inferring the minor allele may not be possible, especially when the MAF is close to 0.5. Conversely, CaseControl_AF outputs the estimated case and control AFs for which the total sample AF was reported, retaining allele and AF pairing information.

Notably, both methods assume that the total AN is known. In reality, for a given variant, the total AN and the AN for cases and controls may vary across the variants. When available, a vector, rather than a single value, can be used for AN, providing variant-level information about the total AN. Unfortunately, variant-level AN is often not provided in summary statistics.

An interesting scenario for use beyond those explored here is meta-analysis. In a fixed-effects meta-analysis where the assumption is that all studies share the same underlying true effect, we expect the methods to work similarly to the scenarios evaluated here. However, in a meta-analysis using a random-effects or similar framework where the underlying true effects differ in the studies, it is possible that there would be additional variability in the AF estimates not captured by the current methods we present. Best practice is to publish both the meta-analysis summary statistics and the summary statistics for each study, in which case the methods presented here could be easily used.

As SE is more commonly available in GWAS summary data than total AF, we developed a bias correction that can be used when the total sample AF is not available. While bias correction requires harmonization of the observed data with a publicly available data source, such as gnomAD, we find that even a subset of the data, such as chromosome 1, as we use here, is sufficient to estimate and correct for bias. This eases computational and person-time burden and enables bias correction for variants not in the public proxy data. While the bias correction improves the accuracy of the case and control AF estimates, variability and bias in higher MAF bins remain and are especially prominent when the sample size is small. Additionally, a publicly available database containing ancestrally matched proxy AFs is required for the bias correction, highlighting the need for large, public, and ancestrally diverse databases. While gnomAD is an expansive resource for genomics, studies of admixed samples may not have an ancestrally matched gnomAD group to use as the proxy. For bias correction of the Pan-UKBB Diabetes GWAS in AFR individuals, we used gnomAD MAFs from the AFR/AFRAM group as proxies and found that, while not a perfect genetic-similarity match, bias of the estimates was still greatly reduced. Summary data harmonization methods, such as *Summix2*,^23^ can adjust AFs to match genetic similarity between samples and could be used to harmonize the population structure between the proxy and GWAS data here. The use of this bias correction framework for ancestrally diverse or admixed samples requires further investigation.

### Conclusion

We have introduced methods and software to derive case and control AFs from GWAS summary data. These AFs can then be used in downstream analyses, such as in association studies as external controls, case-case GWAS, and meta-analysis. The functions available in the CCAFE package provide researchers with user-friendly and open-source methods to enhance the reuse of publicly available genetic summary statistics.

## Data and code availability

The CCAFE software is available for download on GitHub (https://github.com/wolffha/CCAFE/) and through Bioconductor (https://bioconductor.org/packages/CCAFE/). The code used to generate simulated datasets and perform analyses is available at https://github.com/wolffha/CaseControlAF_manuscript.

## Acknowledgments

This work was supported by the 10.13039/100000051National Human Genome Research Institute (R35HG011293, R01HG011345, U01HG011715, and R01HL151152). This work also used the computing resources at the Center for Computational Mathematics, University of Colorado Denver, including the Alderaan cluster, supported by the 10.13039/100000001National Science Foundation award OAC-2019089.

## Declaration of interests

A.E.H. is an associate editor for *HGG Advances*. C.R.G. owns stock in 23andMe, Inc.

## Web resources

Bioconductor - CCAFE, https://bioconductor.org/packages/CCAFE/
CRAN - PhenotypeSimulator, https://cran.r-project.org/web/packages/PhenotypeSimulator/index.html
EBI - Prostate Cancer GWAS, https://www.ebi.ac.uk/gwas/studies/GCST006085
GitHub - CCAFE, https://github.com/wolffha/CCAFE/
GitHub - CaseControlAF_manuscript, https://github.com/wolffha/CaseControlAF_manuscript
gnomAD, https://gnomad.broadinstitute.org/downloads
Pan-UK Biobank, https://pan-dev.ukbb.broadinstitute.org/docs/per-phenotype-files/index.html

## Supplemental information

Document S1. Figures S1–S17, Tables S1–S5, and Notes S1–S6

Document S2. Article plus supplemental information
