# Supplementary material for: CCAFE: Estimating case and control allele frequencies from GWAS summary statistics
Source: HGG Adv. 2026 Apr 20;7(3):100616. doi: 10.1016/j.xhgg.2026.100616 (PMC13347939; doi:10.1016/j.xhgg.2026.100616)
Supplement: Document S1. Figures S1–S17, Tables S1–S5, and Notes S1–S6 [file mmc1.pdf]

HGGA, Volume 7

Supplemental information

CCAFE: Estimating case and control allele frequencies from GWAS summary statistics

Hayley R. Stoneman, Hugo Lemus Gomez, Adelle Price, Christopher R. Gignoux, and Audrey E. Hendricks

Note S1. Full Derivation for CaseControl_AF

Known:

$$AF_{total} = \frac{(N_{case}AF_{case} + N_{control}AF_{control})}{N_{total}} \tag{1}$$

$$OR = \frac{ad}{bc} \tag{2}$$

Where:

$a = 2N_{case} * AF_{case}$

$b = 2N_{case}(1 - AF_{case})$

$c = 2N_{control} * AF_{control}$

$d = 2N_{control}(1 - AF_{control})$

Find $AF_{control}$ and AF_{case}:

Substitute for $a, b, c, \& \ d$ into equation (2) and simplify:

$$OR = \frac{(2N_{case} * AF_{case})[2N_{control}(1 - AF_{control})]}{[2N_{case}(1 - AF_{case})](2N_{control} * AF_{control})} \tag{3}$$

$$OR = \frac{AF_{case}(1 - AF_{control})}{(1 - AF_{case})AF_{control}} \tag{4}$$

Substitute for AF_{case} using equation (1) and distribute:

$$OR = \frac{(\frac{N_{total}}{N_{case}}AF_{total} - \frac{N_{control}}{N_{case}}AF_{control} \ )(1 - AF_{control})}{[1 - \frac{N_{total}}{N_{case}}AF_{total} + \frac{N_{control}}{N_{case}}AF_{control}]AF_{control}} \tag{5}$$

$$OR = \frac{\frac{N_{total}}{N_{case}}AF_{total} - AF_{control}\frac{N_{control}}{N_{case}} - AF_{control}\left(\frac{N_{total}}{N_{case}}AF_{pop}\right) + AF_{control}^2(\frac{N_{control}}{N_{case}})}{AF_{control}[1 - \left(\frac{N_{total}}{N_{case}}AF_{total}\right)] + AF_{control}^2(\frac{N_{control}}{N_{case}})} \tag{6}$$

$$\begin{aligned} AF_{control}^2\left(\frac{N_{control}}{N_{case}}\right)OR + AF_{control}\left[1 - \left(\frac{N_{total}}{N_{case}}AF_{total}\right)\right]OR \\ = AF_{control}^2\left(\frac{N_{control}}{N_{case}}\right) - AF_{control}\left(\frac{N_{control}}{N_{case}} + \frac{N_{total}}{N_{case}}AF_{total}\right) + \frac{N_{total}}{N_{case}}AF_{total} \end{aligned} \tag{7}$$

Arrange as quadratic equation:

$$AF_{control}^2\left[\frac{N_{control}}{N_{case}}(OR - 1)\right] + AF_{control}\left[OR\left(1 - \left(\frac{N_{total}}{N_{case}}AF_{total}\right)\right) + \frac{1}{N_{case}}(N_{control} + N_{total}AF_{total})\right] - \frac{N_{total}}{N_{case}}AF_{total} = 0 \tag{8}$$

Let:

$$\left[a = \frac{N_{control}}{N_{case}}(OR - 1)\right] \tag{9}$$

$$b = \left[OR\left(1 - \left(\frac{N_{total}}{N_{case}}AF_{total}\right)\right) + \frac{1}{N_{case}}(N_{control} + N_{total}AF_{total})\right] \quad (10)$$

$$c = -\frac{N_{total}}{N_{case}}AF_{total} \quad (11)$$

$$AF_{control}^2 a + AF_{control} b + c = 0 \quad (12)$$

Choose the root greater than 0 and less than 1 to be $AF_{control}$ where x_1, x_2 are the roots

$$x_{1,2} = \frac{-b \pm \sqrt{b^2 - 4ac}}{2a} \quad (13)$$

$$AF_{control} = \begin{cases} x_1, & if\ 0 \le x_1 \le 1 \\ x_2, & otherwise \end{cases}$$

Use the calculated $AF_{control}$ to solve for AF_{case} where:

$$AF_{case} = \frac{N_{total}}{N_{case}}AF_{total} - \frac{N_{control}}{N_{case}}AF_{control} \quad (14)$$

Note S2. Simulations Show There is Only One Root [0,1]

Given the quadratic in equation (8) and the coefficients in equations (9 – 11), we sought to determine the possible solutions for the roots as shown in equation (13). Keeping N_{total} constant at 10,000, we examined 144 different scenarios with varying AF, OR, N_{case}, and $N_{control}$ as shown in Table S1 below (all combinations and results in Table S2).

We used the values of the parameters to compute *a, b, c* as shown in equations (9-11), followed by calculation of the two roots using equation (13). Root 1 (x_1) and root 2 (x_2) were calculated as follows:

$$x_1 = \frac{-b - \sqrt{b^2 - 4ac}}{2a} \quad (15)$$

$$x_2 = \frac{-b + \sqrt{b^2 - 4ac}}{2a} \quad (16)$$

We observed the results shown in Figure S1, indicating that for possible combinations of parameters, only one root (x_2) lies within [0,1]. Also note that when $a > 0$, $x_1 < 0$, and when $a < 0$, $x_1 > 1$.

Note S3. Proof That Only 1 Root Lies Within [0,1]

We first note that there are two conditions for which we may need to assess the bounds of the roots, $a > 0\ and\ a < 0$. Looking at equation (9), we see that a is positive when $OR > 1$ and a is negative when $OR < 1$. Since we are working with biallelic variants, we are able to simply switch the allele ($1 - AF$) which will switch the OR to either greater than or less than 1. Thus, we only need to solve the proof under one condition; specifically, we will show that only 1 root lies within [0,1] for the case in which $a > 0$.

We start with the following inequality

$$a + b + c \geq 0 \tag{17}$$

We can show that this is true by plugging in our values for a, b, c from equations (9-11). First, we rearrange each equation to have the same denominator.

Rearrange:

$$a = \frac{OR * N_{control} - N_{control}}{N_{case}} \tag{18}$$

Rearrange:

$$b = OR\left(\frac{N_{case} - N_{total}AF_{total}}{N_{case}}\right) + \frac{N_{control} + N_{total}AF_{total}}{N_{case}} \tag{19}$$

$$b = \frac{OR * N_{case} - OR * N_{total}AF_{total} + N_{control} + N_{total}AF_{total}}{N_{case}} \tag{20}$$

Rearrange:

$$c = -\frac{N_{total}AF_{total}}{N_{case}} \tag{21}$$

Now plug in equations (18, 20, 21) into (17).

$$\frac{OR * N_{control} - N_{control} + OR * N_{case} - OR * N_{total}AF_{total} + N_{control} + N_{total}AF_{total} - N_{total}AF_{total}}{N_{case}} \geq 0 \tag{22}$$

$$\frac{OR(N_{case} + N_{control}) - OR * N_{total}AF_{total}}{N_{case}} \geq 0 \tag{23}$$

$$\frac{OR * N_{total} - OR * N_{total}AF_{total}}{N_{case}} \geq 0 \tag{24}$$

$$\frac{OR * N_{total}(1 - AF_{total})}{N_{case}} \geq 0 \tag{25}$$

Since we know $0 \leq AF_{total} \leq 1, OR > 0, N_{total} > 0, N_{case} > 0$ we know that the inequality in (25) is true, thus proving that under these conditions, the inequality in (17) is true.

We can now use equation (17) to prove that $x_2 < 1$ (as a reminder, x_2 is defined in equation (16)).

$$a + b + c \geq 0 \tag{17}$$

Rearrange

$$a + b \geq -c \tag{26}$$

Multiply by $4a$ (reminder $a > 0$)

$$4a^2 + 4ab \geq -4ac \tag{27}$$

Add b^2 to both sides

$$4a^2 + 4ab + b^2 \geq b^2 - 4ac \tag{28}$$

Factor the right side

$$(2a + b)^2 \geq b^2 - 4ac \tag{29}$$

Take the square root of both sides

$$2a + b \geq \sqrt{b^2 - 4ac} \tag{30}$$

Subtract b and divide by $2a$

$$1 \geq \frac{-b + \sqrt{b^2 - 4ac}}{2a} \tag{31}$$

Substitute equation (16) in the right side

$$1 \geq x_2 \tag{32}$$

We now use the following inequality to prove that $x_2 \geq 0$ as well. We start with the following:

$$-4ac \geq 0 \tag{33}$$

We know that the equality shown in (33) is true because $c < 0$ and we assumed that we are under the condition in which $a > 0$, making the left side of the equality a positive number (or 0).

Add b^2 to both sides

$$b^2 - 4ac \geq b^2 \tag{34}$$

Take the square root of both sides

$$\sqrt{b^2 - 4ac} \geq b \tag{35}$$

Subtract b and divide by $2a$

$$\frac{-b + \sqrt{b^2 - 4ac}}{2a} \geq 0 \tag{36}$$

Substitute equation (16) in the left side

$$x_2 \geq 0 \tag{37}$$

Finally, we show that the other root, x_1 is outside of the valid range of solutions for $AF_{control}$

As a reminder:

$$-4ac \geq 0 \tag{33}$$

Add b^2 to both sides

$$b^2 - 4ac \geq b^2 \tag{38}$$

Take the square root of both sides, this time keeping the negative b

$$\sqrt{b^2 - 4ac} \geq -b \tag{39}$$

Subtract square root term and divide by $2a$

$$0 \geq \frac{-b - \sqrt{b^2 - 4ac}}{2a} \tag{40}$$

Substitute equation (15) in the right side

$$0 \geq x_1 \tag{41}$$

We have now proved that under the assumption that $a > 0$, $0 \leq x_2 \leq 1$, and $x_1 \leq 0$, thus proving that there is only one valid solution for $AF_{control}$.

Note S4. Recommended Number of Variants Per MAF Bin for SE Bias Correction

In order to determine the recommended number of overlapping variants between the proxy data and the user dataset we assessed the impact on the bias correction for the Pan-UKBB diabetes data using

gnomAD as a proxy for both African and European samples. We again used the 1,212,618 chromosome one variants that were harmonized with Pan-UKBB (see **2.2.2**). We subset the number of variants (100, 500, 1000, 5000, 10000, 15000, 30000, 50000) per bin ([0, 0.1), [0.1, 0.2), [0.2, 0.3), [0.3, 0.4), [0.4, 0.5]) over 10 replicates and plotted the mean and median bias per MAF bin. We compared the bias to the unadjusted estimates for all >9 million genome-wide variants in each sample.

In both Pan-UKBB AFR and EUR samples, we observe minimal difference in the adjusted MAF estimates using any number of variants per bin equal to or greater than 10,000 (**Figure S2-S3**). Therefore, we recommend for best results of bias correction a minimum number of variants per bin of 10,000.

Note S5. Replication of embedded code from ReACt

The function to derive case and control AFs from the SE is embedded within a publicly available meta-analysis software ReACt written in C (https://github.com/Paschou-Lab/ReACt). To provide a standalone function for AF estimation, we translated it into R as the CCAFE software function CaseControl_SE. To ensure that the translation was accurate, we tested whether we could replicate the case-case GWAS (CC-GWAS) results published in Yang et al in R that examined bipolar disorder and schizophrenia GWAS results from the Psychiatric Genomics Consortium in CC-GWAS. When we tested our R implementation of their software using the same data and we were able to replicate the published results.

Note S6. Case AF Derivation for Prostate Cancer GWAS

We selected a prostate cancer PGS based on a GWAS that published OR and control AF for each variant (Schumacher et al., 2018). Using the case and control sample sizes ($N_{case}, N_{control}$) we derived allele counts (AC) for the effect and non-effect alleles using $AC = AF * N$. The OR can be calculated using ACs as follows:

$$OR = \frac{AC_{effect,case} * AC_{non-effect,control}}{AC_{effect,control} * AC_{non-effect,case}} \tag{42}$$

We rearrange this equation to solve for the effect allele AC in cases, as needed for our reference data, as follows:

$$AC_{effect,case} = \frac{OR * AC_{effect,control}}{1 - AC_{effect,control} + OR * AC_{effect,control}} \tag{43}$$

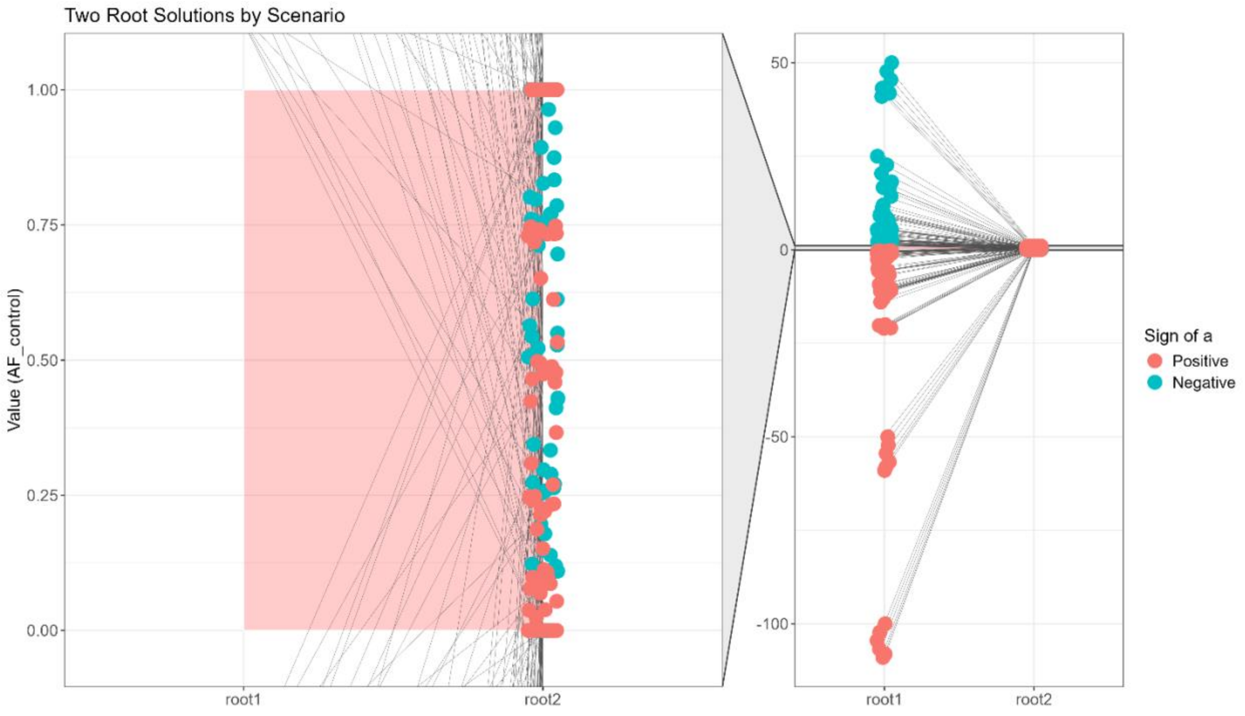

Figure S1. Simulations show only one root solves for valid control AF value. Simulations of 144 different scenarios with varying case and control sample size, OR, and AF were used to calculate the roots (two possible solutions for control AF). Root 2, calculated by equation (16) is always in the valid interval [0,1] (shown in the red rectangle), while root 1, calculated by equation (15) is always outside of the valid interval. We also note that when the coefficient a is positive, root 1 is lower than the valid interval (i.e. < 0) and when a is negative, root 1 is greater than the valid interval (i.e. > 1).

96

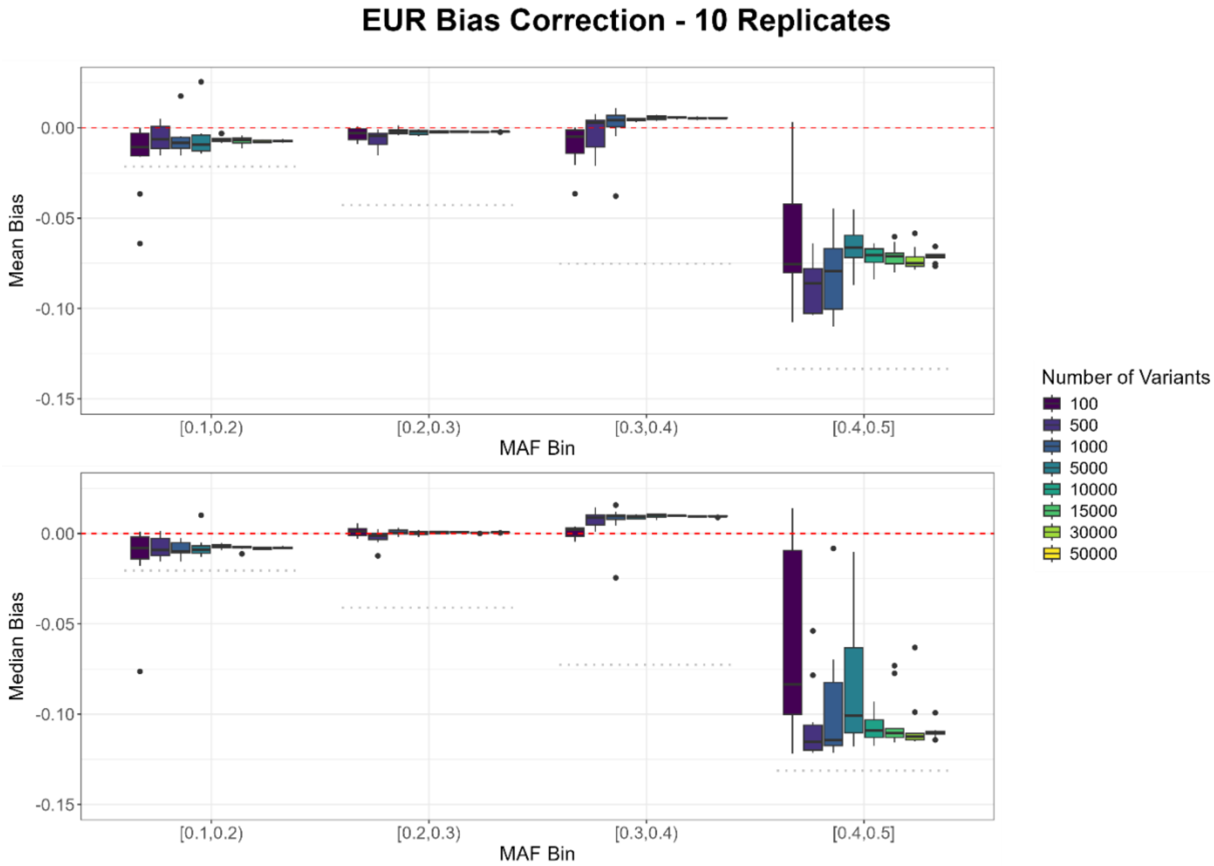

Figure S2. Number of variants needed for bias correction framework to adjust CaseControl_SE estimates using gnomAD as proxies in EUR. The gnomAD v3.1.2 chromosome 1 data (1,212,618 total variants) for the non-Finish European group (NFE) was subset for a given number of variants (shown in the key on the right) per MAF bin (x-axis) for 10 replicates. This subset of variants was used to correct the bias for all >9 million genome-wide variants in the Pan-UKBB EUR diabetes dataset. The mean (top) and median (bottom) bias was calculated per bin. The unadjusted bias is shown with the dotted grey line, while zero bias is shown with the dotted red line.

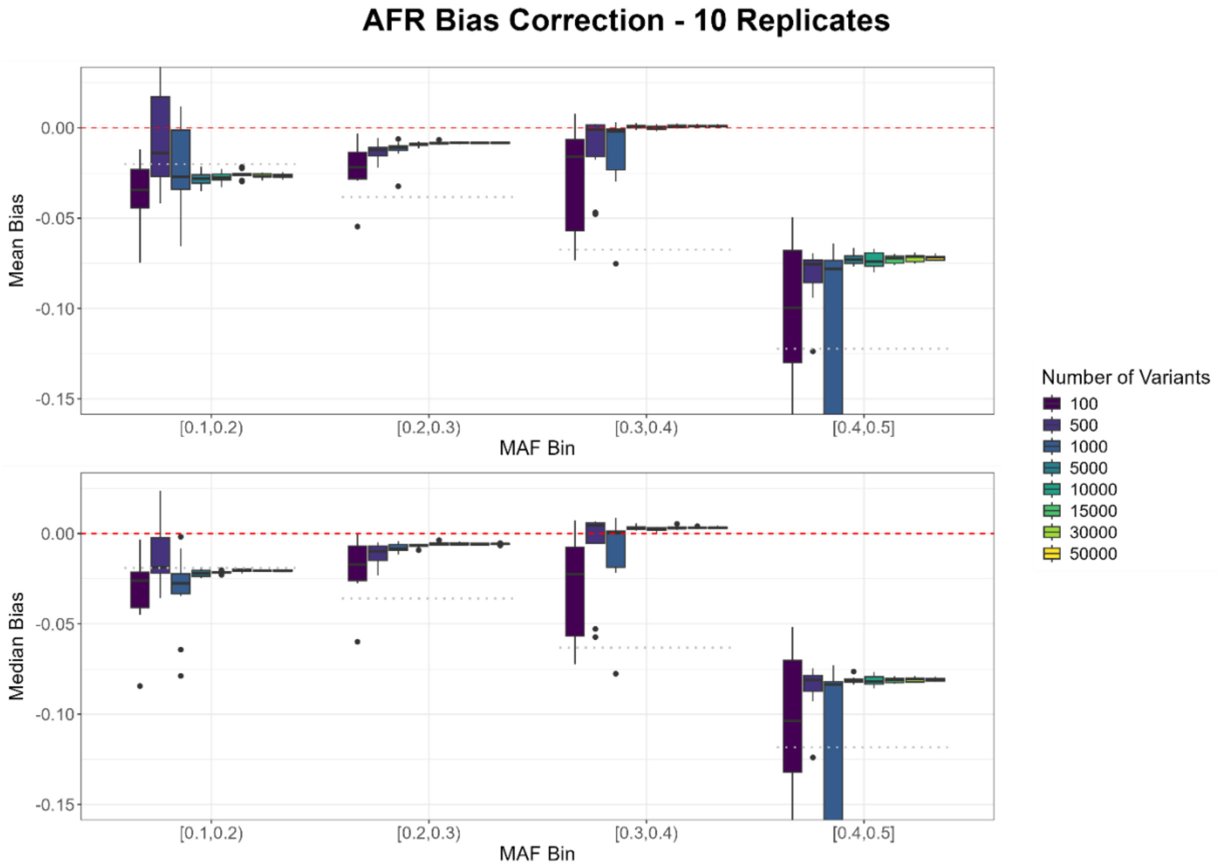

Figure S3. Number of variants needed for bias correction framework to adjust CaseControl_SE estimates using gnomAD as proxies in AFR. The gnomAD v3.1.2 chromosome 1 data (1,212,618 total variants) for the African/African American group (AFR/AFRAM) was subset for a given number of variants (shown in the key on the right) per MAF bin (x-axis) for 10 replicates. This subset of variants was used to correct the bias for all >9 million genome-wide variants in the Pan-UKBB AFR diabetes dataset. The mean (top) and median (bottom) bias was calculated per bin. The unadjusted bias is shown with the dotted grey line, while zero bias is shown with the dotted red line.

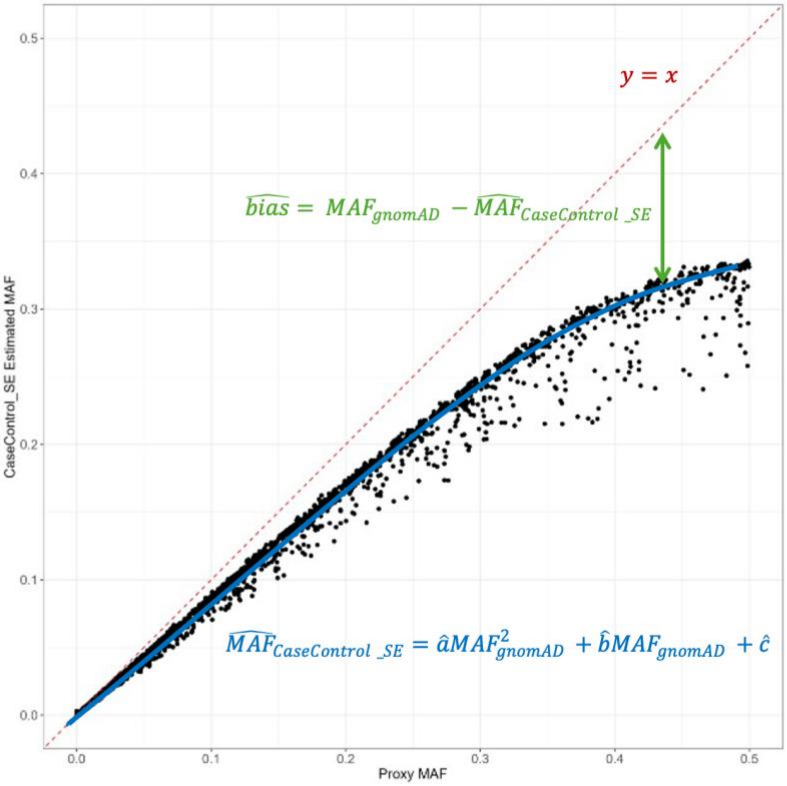

Figure S4. Bias correction framework to adjust CaseControl_SE estimates using gnomAD as proxies. The relationship between the proxy MAF (x-axis) and the estimated MAF (y-axis) is modeled through polynomial regressions (blue). This model is used to estimate the bias (green). The adjusted MAF estimate is estimated by adding the bias to the CaseControl_SE MAF output.

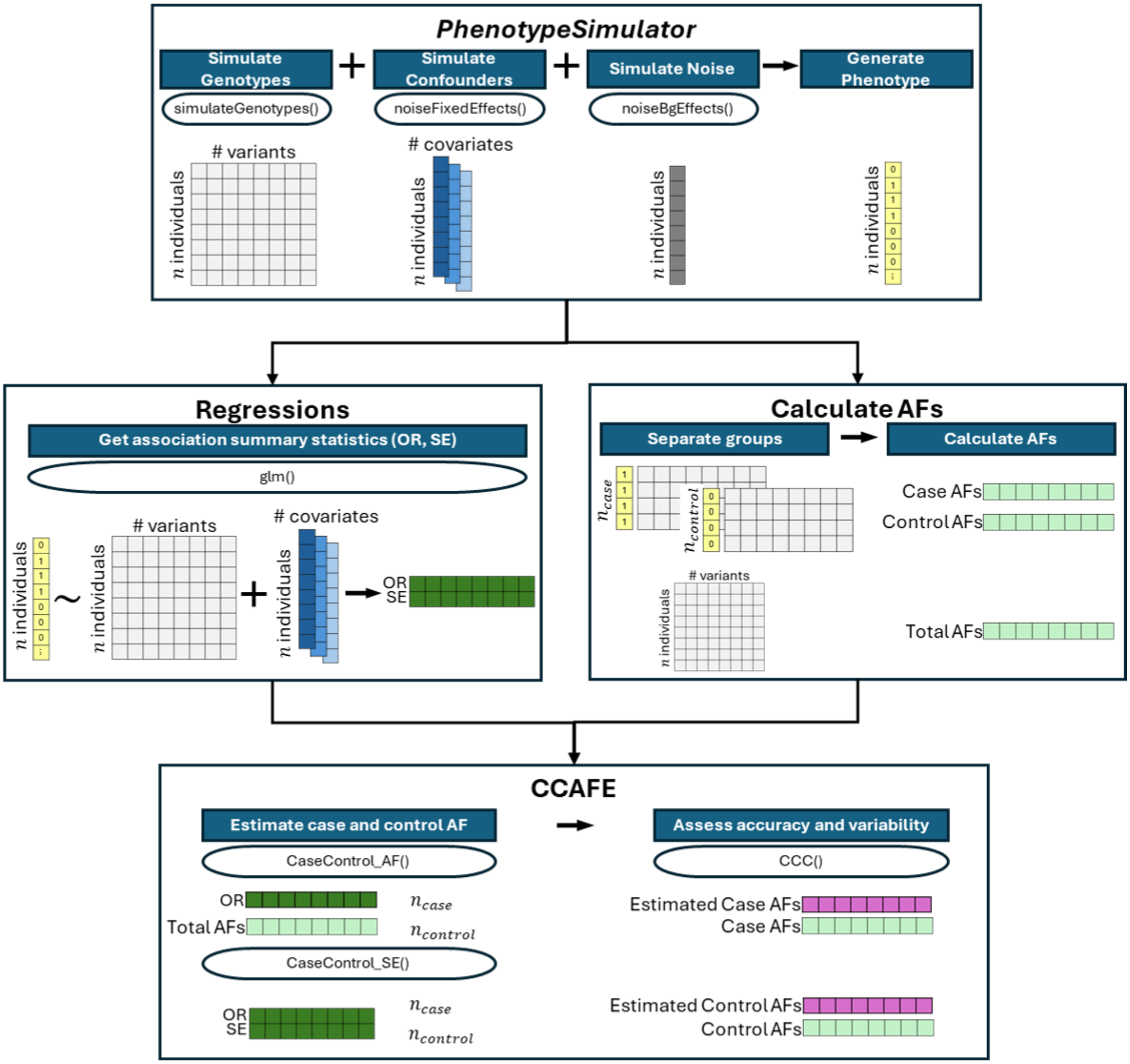

Figure S5. Simulation framework to assess *CCAFE* accuracy and variability. The *PhenotypeSimulator* R package was used to simulate genotypes, a binary phenotype, and covariates. Logistic regression was fit using the simulated genotypes and phenotypes to obtain association summary statistics (OR, SE). The phenotype (i.e., case/control status) was also used to calculate AF for cases and controls. These simulations were used to evaluate CaseControl_AF and CaseControl_SE using the OR, case and control sample sizes, and either total AF or SE respectively The output estimated case and control AFs were compared to the simulated case and control AFs estimating bias, variance, and agreement using Lin's CCC.

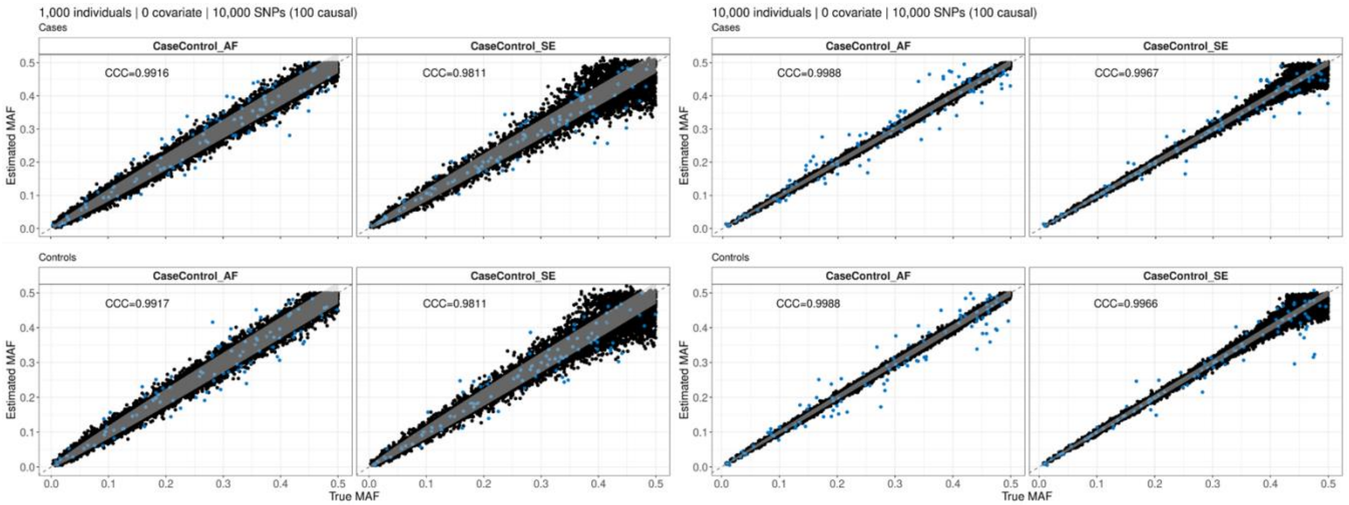

Figure S6. Given no covariates in the original GWAS, CaseControl_AF produces estimates within expected variance while CaseControl_SE has increased variance at high MAFs. Simulated genotypes for 10,000 SNPs of which 100 were causal (blue) and binary (case/control) phenotypes were generated using the PhenotypeSimulator R package with no covariates for N=1,000 (500 cases and 500 controls, **left panel**) and N=10,000 (5,000 cases and 5,000 controls, **right panel**). Logistic regression was used to generate summary statistics. The CCAFE R package was applied to reconstruct the case and control AFs with total AF (1st and 3rd columns) or SE (2nd and 4th columns). The 95% CI of a proportion (AF) given the sample size (N=1000 or 10,000) is shown in the grey ribbon. Using the SE, variance of the estimates was increased for higher MAFs beyond that expected from the CI while variance of the estimates from total AF was similar o the CI. CCC values are reported in Table 1.

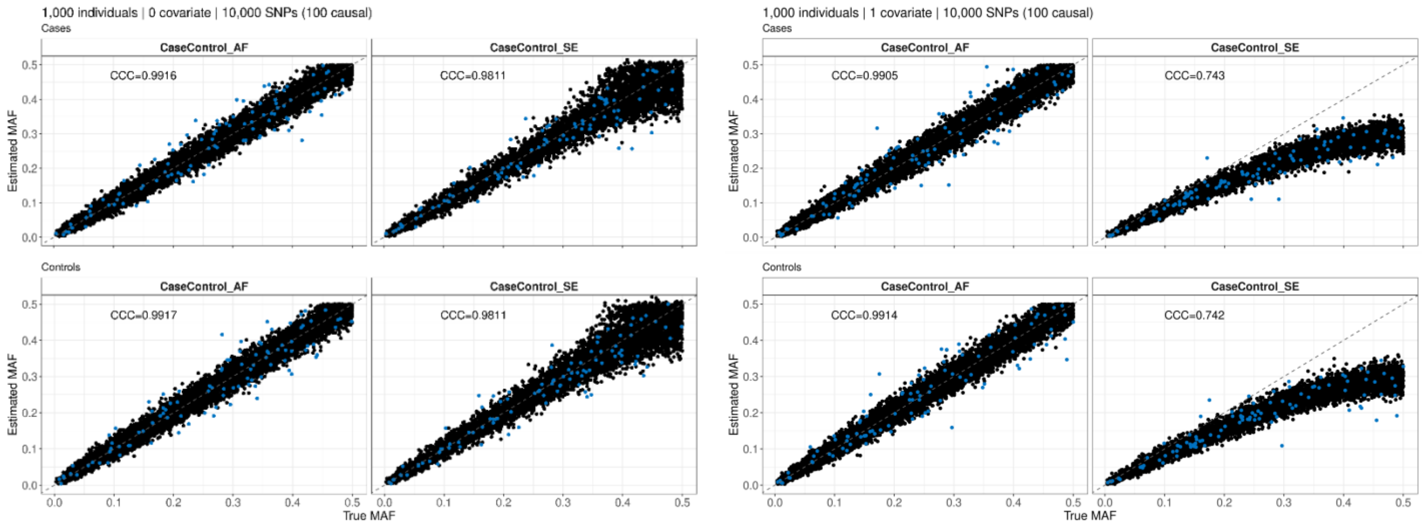

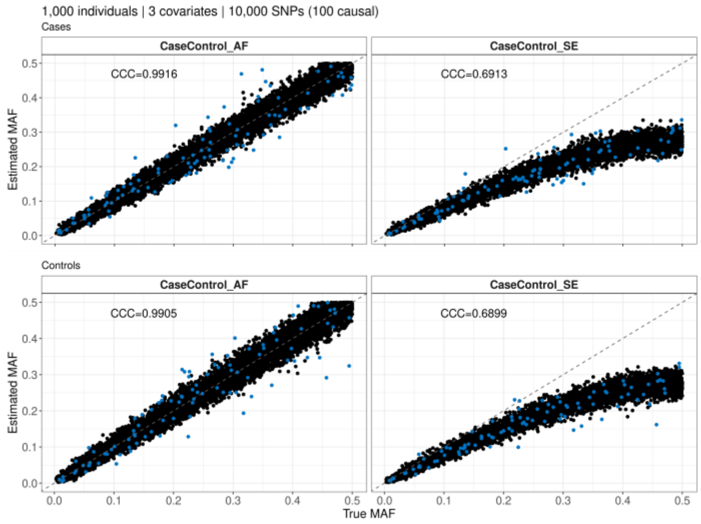

Figure S7. For small sample sizes (N=1,000), CaseControl_AF produces unbiased AF estimates while CaseControl_SE produces biased results in simulation when covariates included. Simulated genotypes and binary (case/control) phenotypes were generated using the PhenotypeSimulator R package for N=1,000 (500 cases and 500 controls). Genotypes for 10,000 SNPs, of which 100 were causal (blue), were generated for 1000 individuals. Logistic regression was used along with 0 (top left), 1 (top right), or 3 (bottom left) simulated covariates to generate summary statistics. The CCAFE R package was used to reconstruct the case and control AFs with total AF (left plots within each quadrant) or SE (right plots within each quadrant). Using the SE, bias existed and was greater for higher MAFs and when more covariates were included. Using total AF was accurate across the simulation parameters evaluated. CCC values are reported in Table 1.

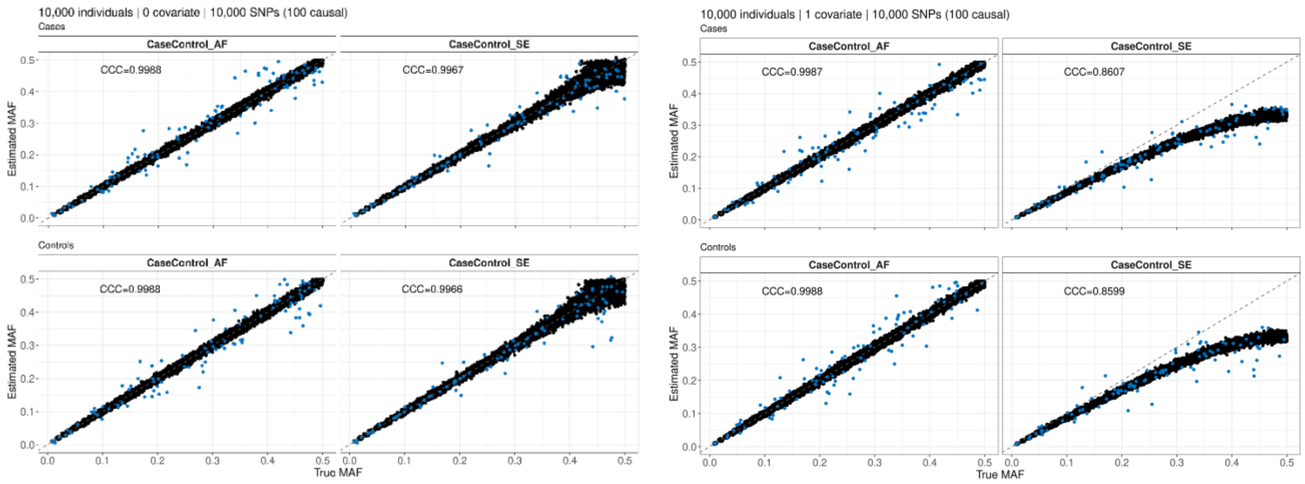

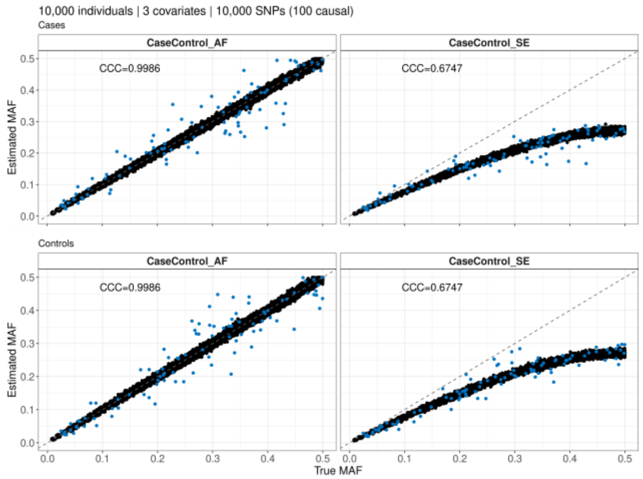

Figure S8. For moderate sample sizes (N=10,000), CaseControl_AF produces unbiased AF estimates while CaseControl_SE produces biased results in simulation when covariates included. Simulated genotypes and binary (case/control) phenotypes were generated using the PhenotypeSimulator R package for N=10,000 (5000 cases and 5000 controls). Genotypes for 10,000 SNPs, of which 100 were causal (blue), were generated for 1000 individuals. Logistic regression was used along with 0 (top left), 1 (top right), or 3 (bottom left) simulated covariates to generate summary statistics. The CCAFE R package was used to reconstruct the case and control AFs with total AF (left plots within each quadrant) or SE (right plots within each quadrant). Using the SE, bias existed and was greater for higher MAFs and when more covariates were included. Using total AF was accurate across the simulation parameters evaluated. CCC values are reported in Table 1.

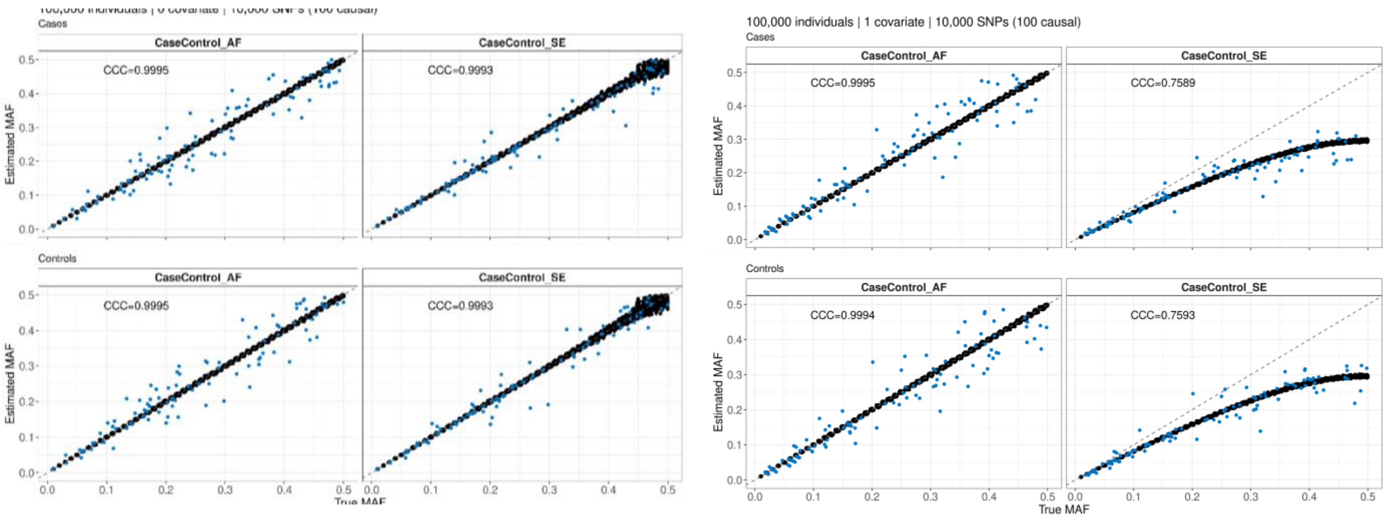

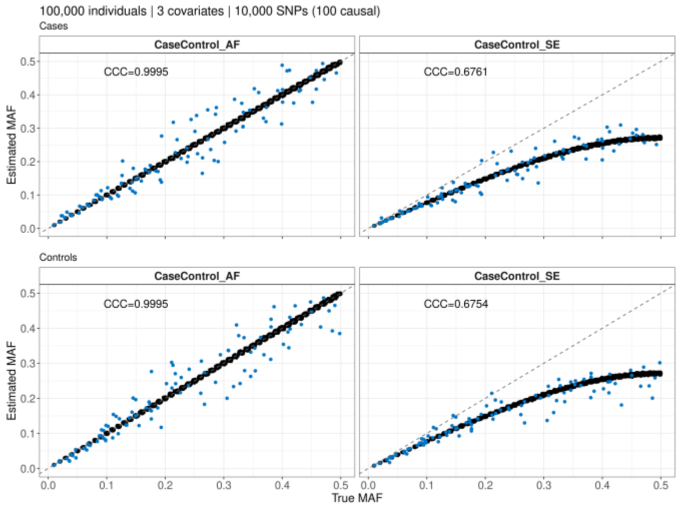

Figure S9. For large sample sizes (N=100,000), CaseControl_AF produces unbiased AF estimates while CaseControl_SE produces biased results in simulation when covariates included. Simulated genotypes and binary (case/control) phenotypes were generated using the PhenotypeSimulator R package for N=100,000 (50,000 cases and 50,000 controls). Genotypes for 10,000 SNPs, of which 100 were causal (blue), were generated for 1000 individuals. Logistic regression was used along with 0 (top left), 1 (top right), or 3 (bottom left) simulated covariates to generate summary statistics. The CCAFE R package was used to reconstruct the case and control AFs with total AF (left plots within each quadrant) or SE (right plots within each quadrant). Using the SE, bias existed and was greater for higher MAFs and when more covariates were included. Using total AF was accurate across the simulation parameters evaluated. CCC values are reported in Table 1.

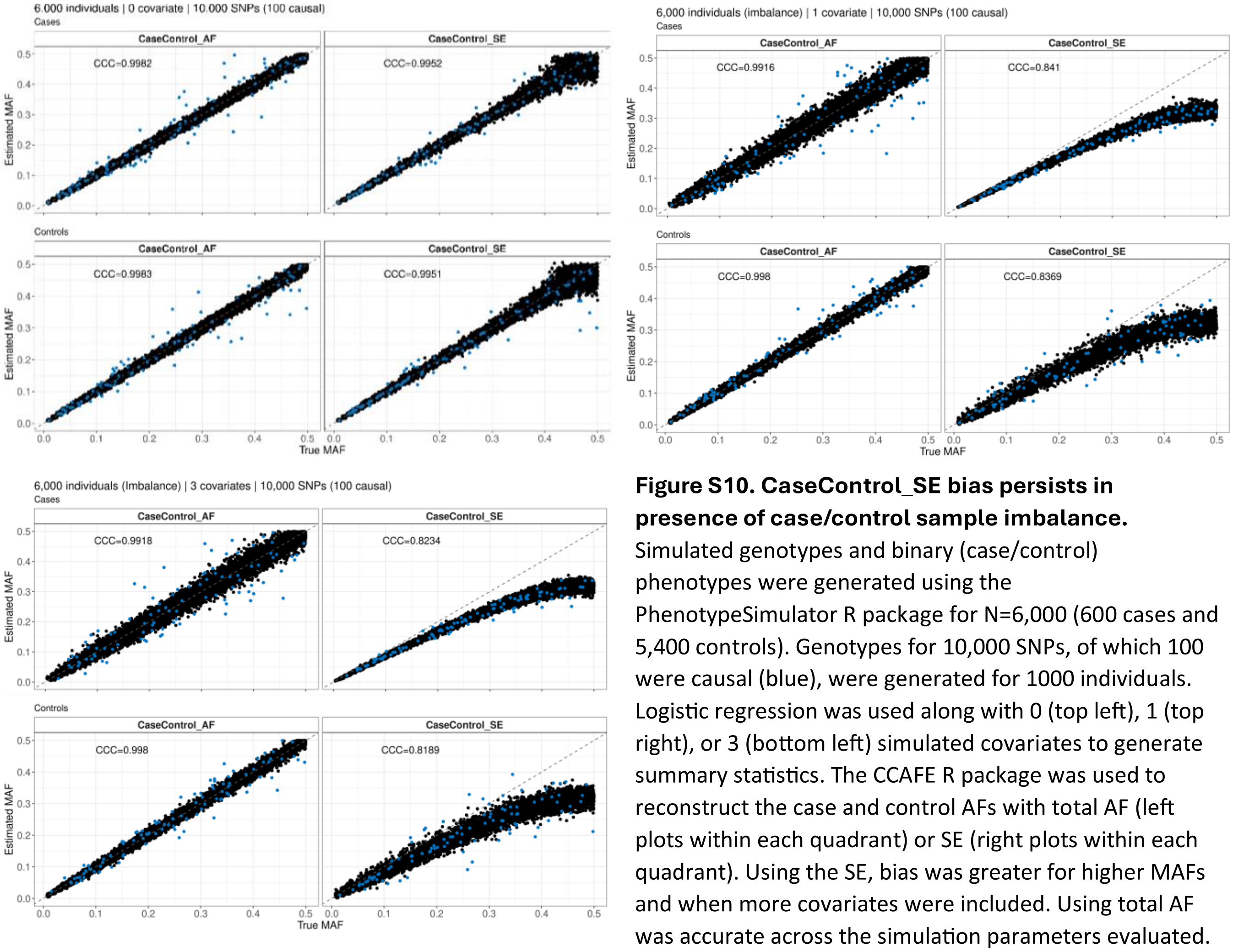

Figure S10. CaseControl_SE bias persists in presence of case/control sample imbalance. Simulated genotypes and binary (case/control) phenotypes were generated using the PhenotypeSimulator R package for N=6,000 (600 cases and 5,400 controls). Genotypes for 10,000 SNPs, of which 100 were causal (blue), were generated for 1000 individuals. Logistic regression was used along with 0 (top left), 1 (top right), or 3 (bottom left) simulated covariates to generate summary statistics. The CCAFE R package was used to reconstruct the case and control AFs with total AF (left plots within each quadrant) or SE (right plots within each quadrant). Using the SE, bias was greater for higher MAFs and when more covariates were included. Using total AF was accurate across the simulation parameters evaluated. CCC values are reported in Table 1.

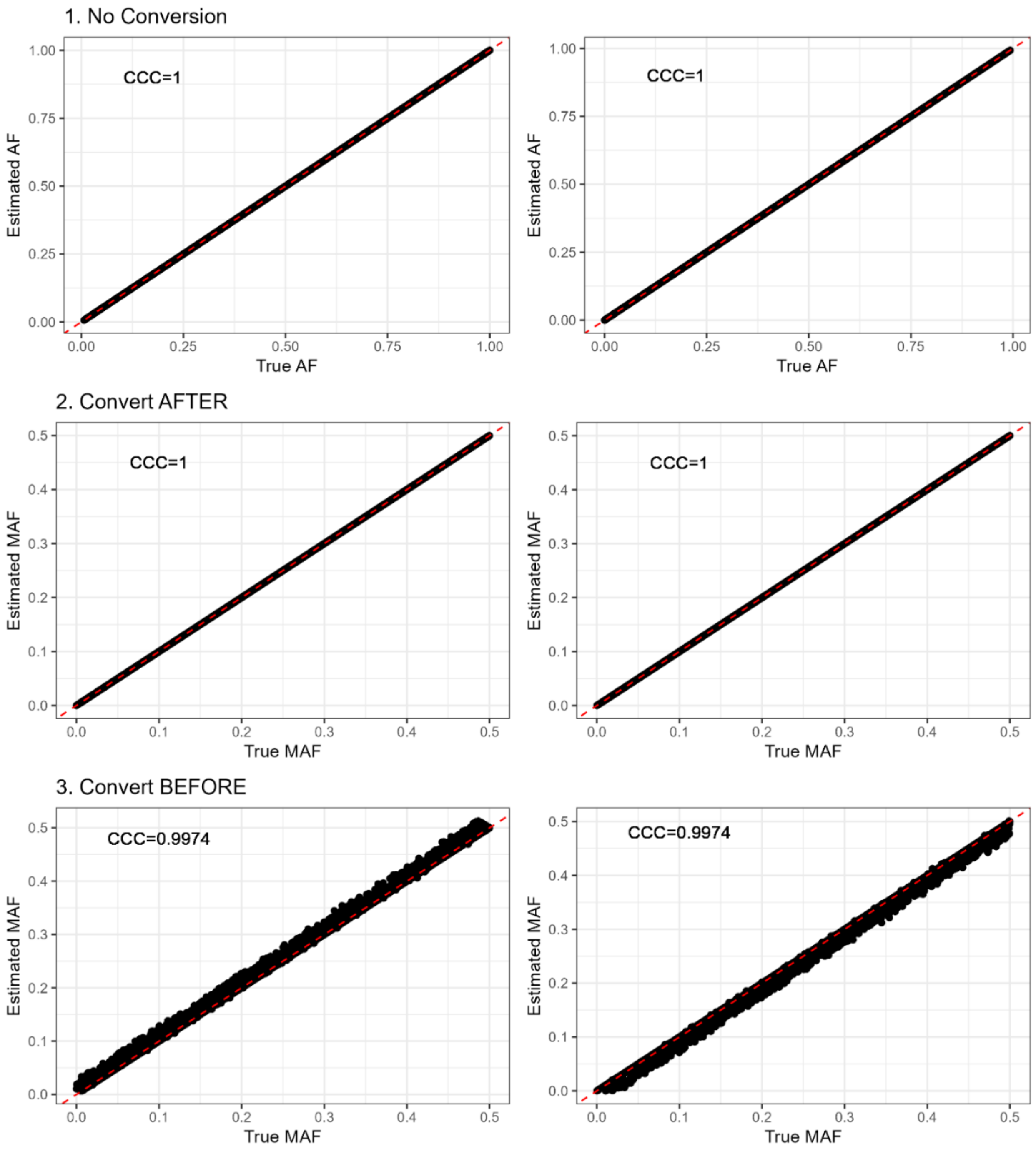

Figure S11. Converting AF to MAF prior to case and control AF estimation increases variability in case and control estimates using CaseControl_AF. Using 2400 simulated variants, three scenarios were used to estimate case AF (Left) and control AF (Right) with CaseControl_AF: 1) using the total AF to estimate case and control AFs 2) using total AF to estimate case and control AFs then converting to minor AF (MAF) 3) converting total AF to MAF and estimating case and control MAFs. Converting first to MAF (scenario 3) introduces variability while using AF (scenario 1) or converting to MAF after estimation (scenario 2) does not introduce variability. Since CaseControl_SE requires converting AF to MAF prior to estimation, additional variability is likely introduced due to this step. Lin's CCC is reported between the true and estimated AF.

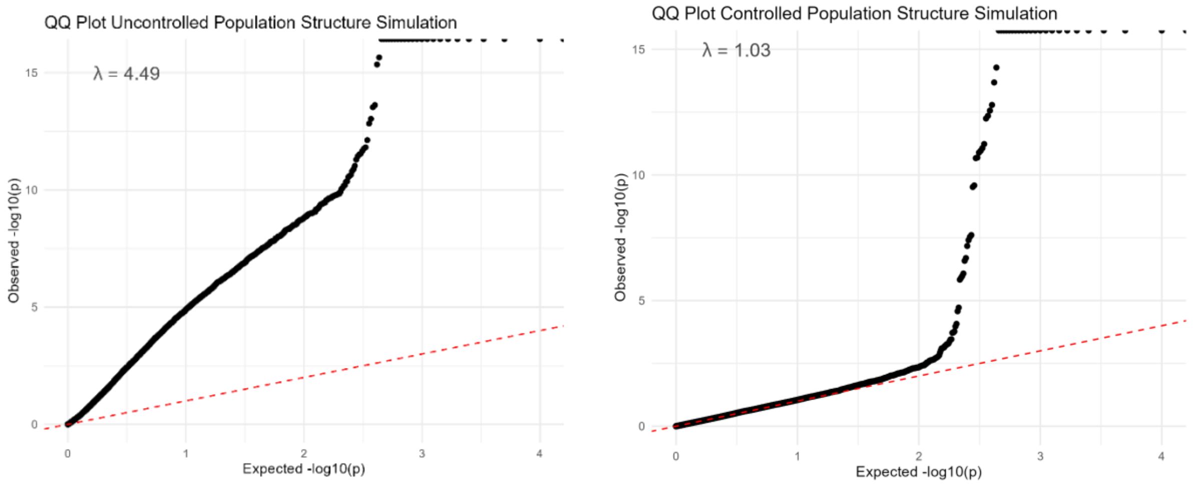

Figure S12. **Population stratification simulations correct genomic inflation.** QQ-plots for the 10,000 variants with simulated population structure without (left) and with (right) correction using principal components analysis. P-values below 10^-16 were rounded resulting in the cap seen here.

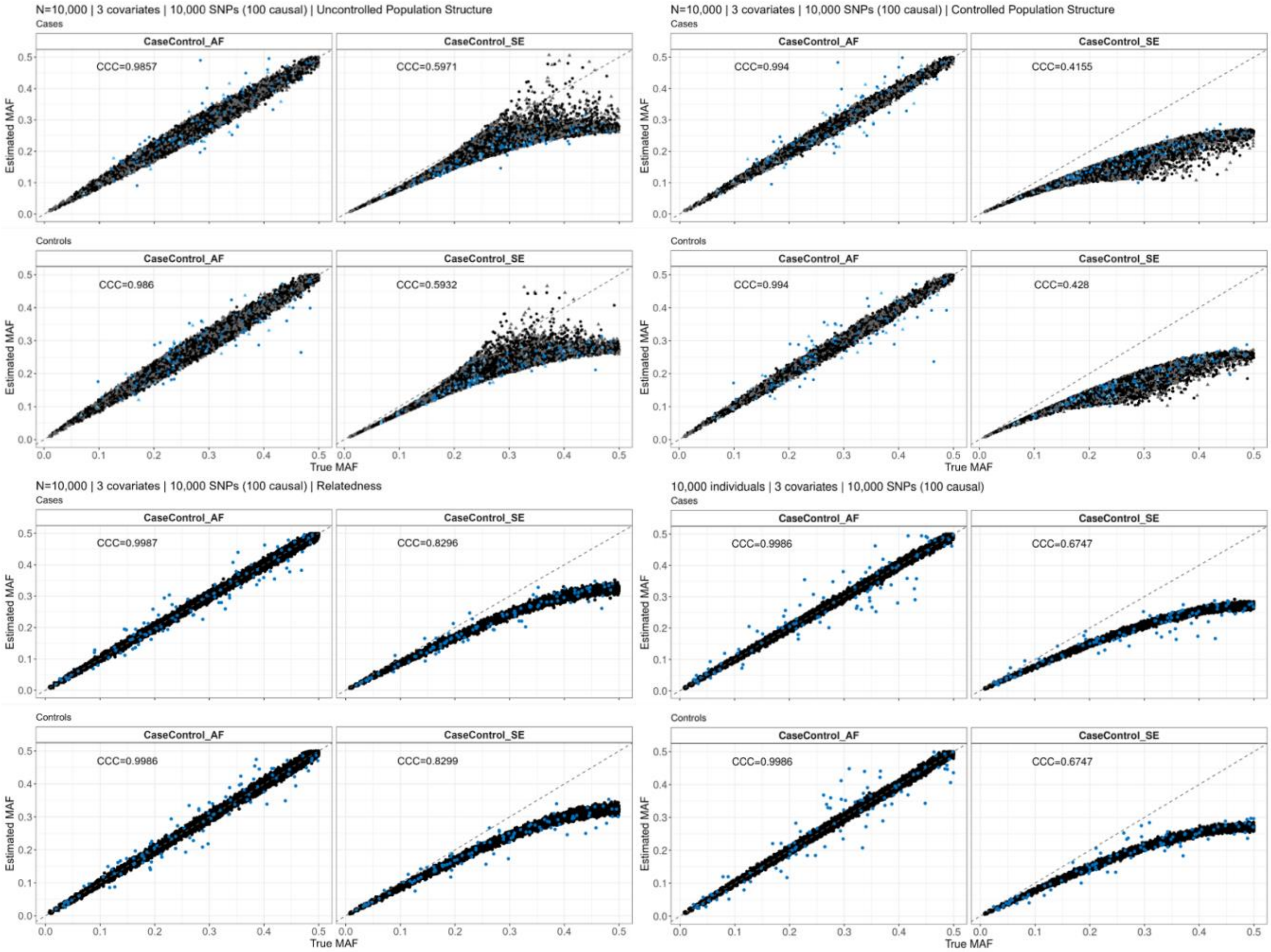

Figure S13. **Uncontrolled population stratification increases AF estimate variability, but not bias; relatedness has no noticeable effects.** Genotypes for 10,000 variants, of which 100 were causal (blue circles) and 9,900 were non-causal (black circles), were generated for 5,000 cases and 5,000 controls. For the **top panels**, 10% of variants (1,000) were simulated to be affected by population stratification (light blue and grey triangles for causal and non-causal respectively). Logistic regression was used along with 3 covariates to generate per variant summary statistics, without **(top left)** or with **(top right)** adjustment for population structure using 10 PCs. While adjusting for population structure resulted in lower variability in AF estimates for both CaseControl_AF and CaseControl_SE compared to not adjusting, AFs derived from GWAS summary statistics that had population stratification (both adjusted and unadjusted) had higher variability compared to summary statistics from GWAS without population structure **(bottom right)**. In the **bottom left** panel, relatedness was simulated to be 36% of the total phenotypic variance and did not have noticeable impact to the variance or bias of the AF estimates for either method.

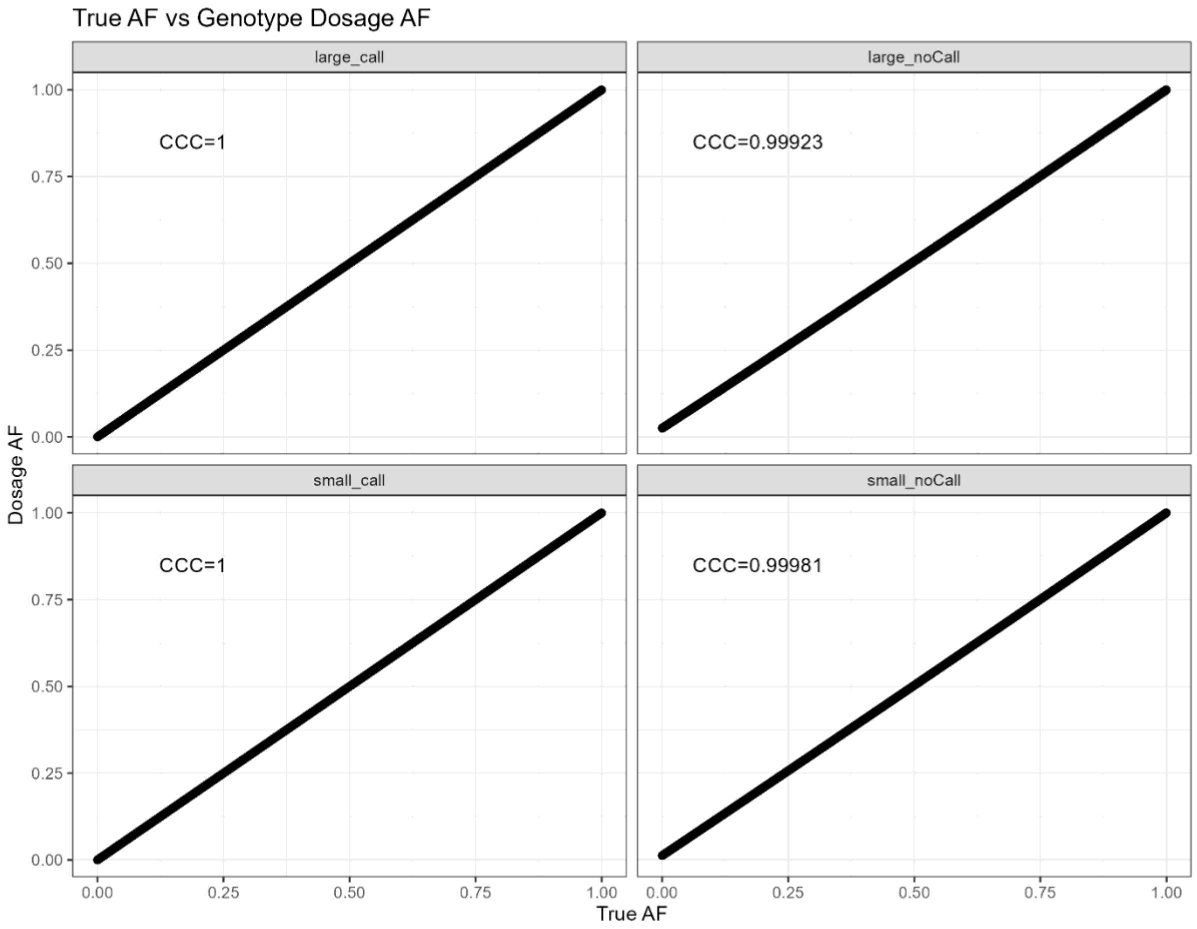

Figure S14. **Genotype dosage simulations.** Allele counts for bialleic variants (0,1,2) were simulated for 10,000 variants and 10,000 individuals. These counts were used to calculate exact allele frequency. Allele dosage with small (+/- 0.1) and large (+/-0.2) variability were simulated and used directly to calculate AF (right). Alleles were then called and used to calculate AF. These results were then compared to the exact AF (left). Lin's CCC was calculated comparing the exact simulated AF to the AFs calculated with added variability and with and without the allele calling.

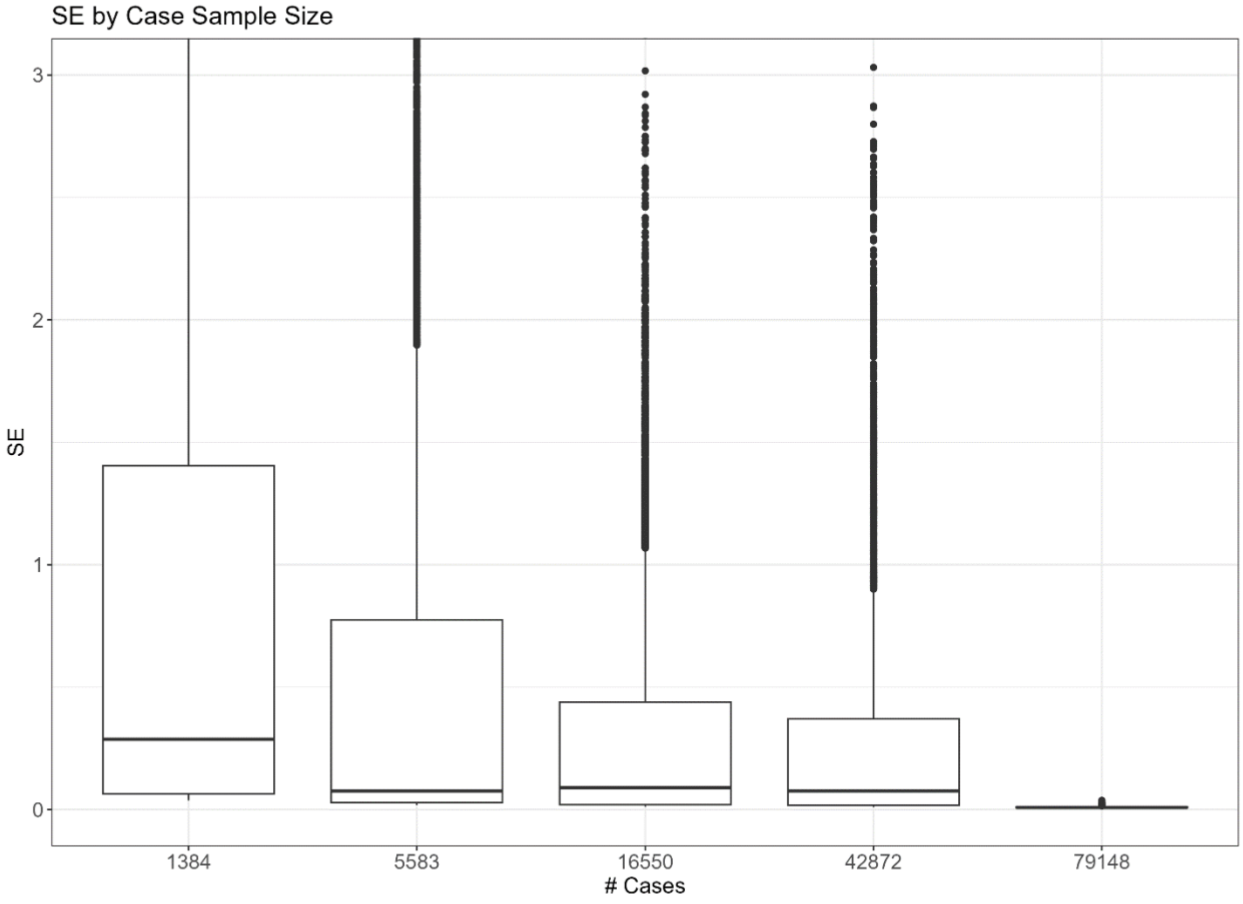

Figure S15. Larger sample sizes have smaller SE and less variability in SE. Here we examined the relationship between the case sample size (x-axis) and the standard error (y-axis). Studies with larger case sample sizes have a smaller SE including the minimum observed SE. Additionally, larger case sample sizes result in less variability in the SE across the variants. For each boxplot the center line shows the median and the upper and lower hinge represent the 75th and 25th percentile, respectively. The upper and lower whiskers are the largest and smallest values no more than 1.5* interquartile range (IQR) from the hinge.

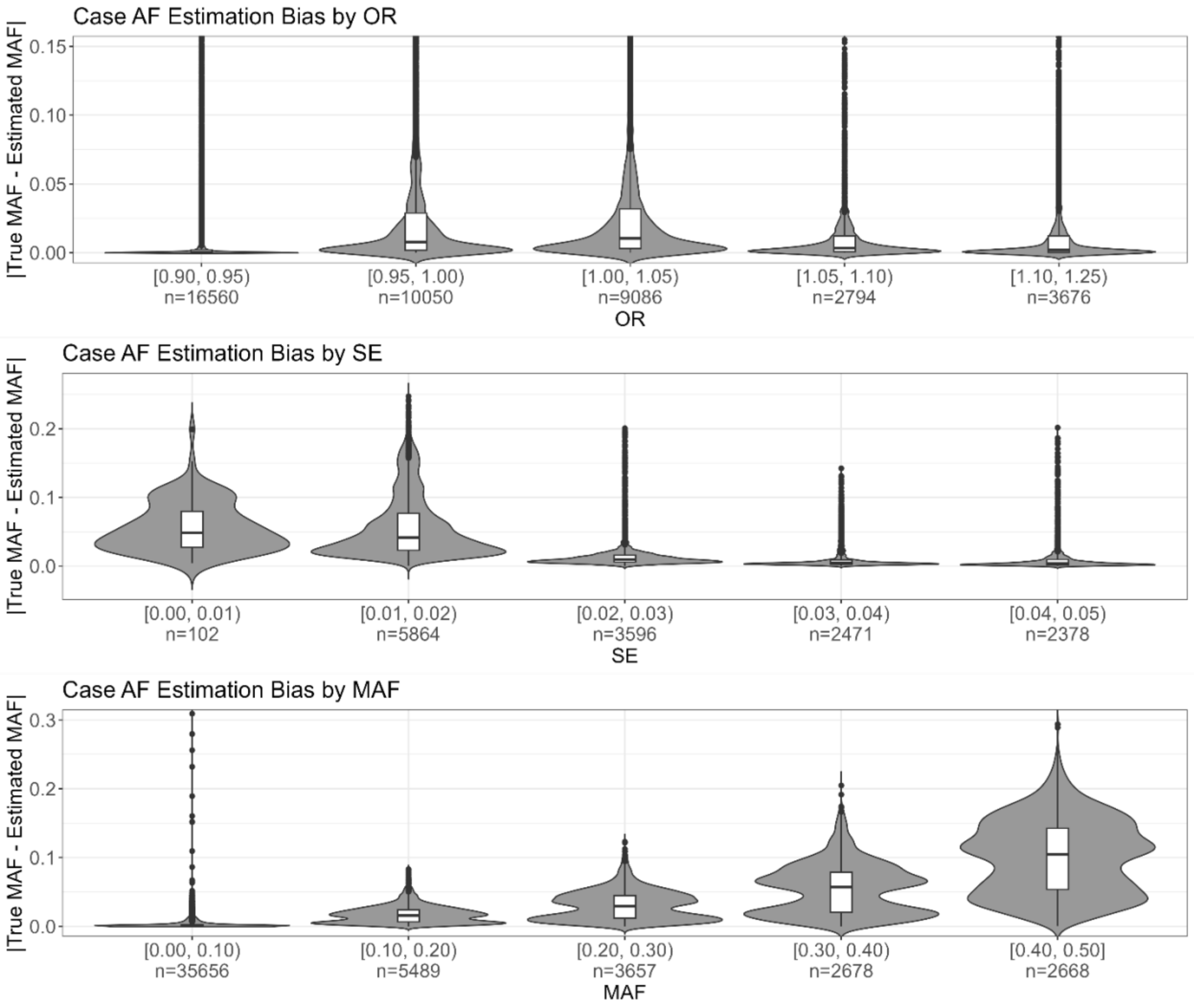

Figure S16. CaseControl_SE has high bias at large MAF and small SE. The difference between the true MAF and CaseControl_SE estimated MAF is compared across different bins for the OR (top), SE (middle), and MAF (bottom). Here the results from the 148 prostate cancer variants and 25,000 randomly sampled variants from Pan-UKBB diabetes in AFR and EUR are aggregated and plotted, with the number of SNPs in each bin shown on the x-axis. We see that bias is higher for smaller SE and larger MAF

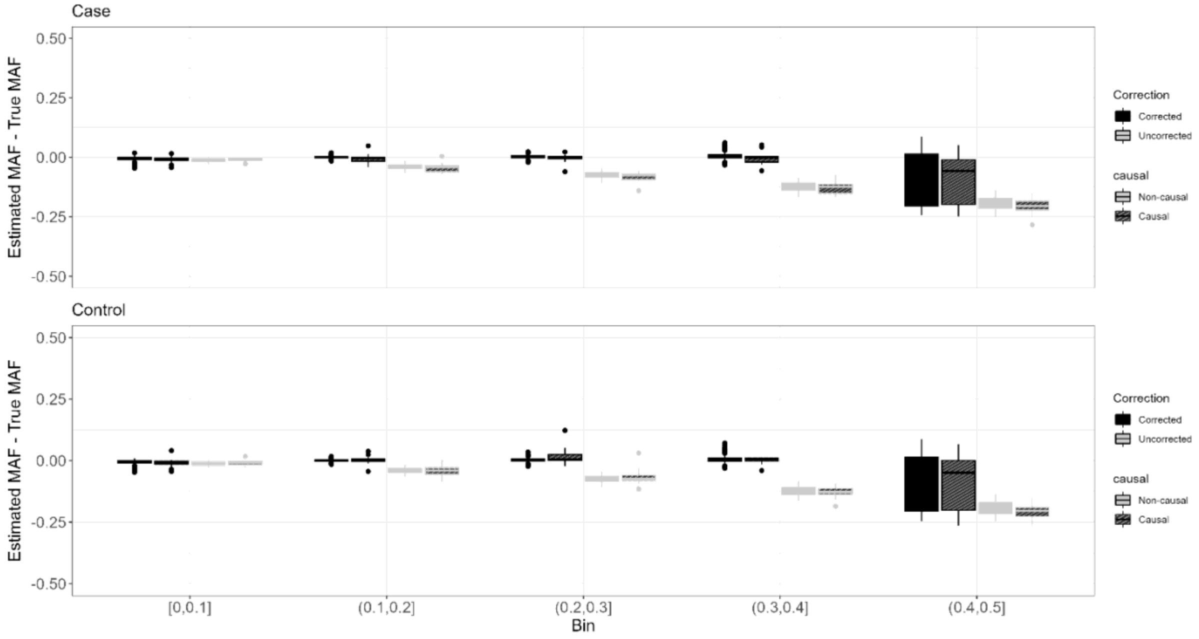

Figure S17. **Simulations show reduced bias using correction framework.** For the simulation of 10,000 variants (100 causal) for 10,000 individuals with 3 covariates, a proxy sample was also simulated to test the bias correction framework. The corrected estimates have much less bias than the uncorrected, but variability remains in the largest minor allele frequency bin. There is no notable difference in bias between the causal and non-causal variants before or after correction.

Table S1. Simulation scenarios to test root solutions

Parameter	Values
N_{case}	1000, 5000, 9000
$N_{control}$	9000, 5000, 1000
N_{total}	10000
AF_{total}	0, 0.1, 0.25, 0.5, 0.75, 1
OR	0.1, 0.4, 0.6, 0.8, 1.1, 1.2, 3, 5

Table S2. Simulations show only one possible root (root2) falls within valid interval [0,1] for control AF solution

OR	AF	N_case	N_control	N_total	a	b	c	root1	root2	sim
0.1	0	5000	5000	10000	-0.9000	1.1000	0.0000	1.2222	0.0000	1
0.4	0	5000	5000	10000	-0.6000	1.4000	0.0000	2.3333	0.0000	2
0.6	0	5000	5000	10000	-0.4000	1.6000	0.0000	4.0000	0.0000	3
0.8	0	5000	5000	10000	-0.2000	1.8000	0.0000	9.0000	0.0000	4
0.1	0	9000	1000	10000	-0.1000	0.2111	0.0000	2.1111	0.0000	5
0.4	0	9000	1000	10000	-0.0667	0.5111	0.0000	7.6667	0.0000	6
0.6	0	9000	1000	10000	-0.0444	0.7111	0.0000	16.0000	0.0000	7
0.8	0	9000	1000	10000	-0.0222	0.9111	0.0000	41.0000	0.0000	8
0.1	0	1000	9000	10000	-8.1000	9.1000	0.0000	1.1235	0.0000	9
0.4	0	1000	9000	10000	-5.4000	9.4000	0.0000	1.7407	0.0000	10
0.6	0	1000	9000	10000	-3.6000	9.6000	0.0000	2.6667	0.0000	11
0.8	0	1000	9000	10000	-1.8000	9.8000	0.0000	5.4444	0.0000	12
0.1	0.1	5000	5000	10000	-0.9000	1.2800	-0.2000	1.2435	0.1787	13
0.4	0.1	5000	5000	10000	-0.6000	1.5200	-0.2000	2.3941	0.1392	14
0.6	0.1	5000	5000	10000	-0.4000	1.6800	-0.2000	4.0774	0.1226	15
0.8	0.1	5000	5000	10000	-0.2000	1.8400	-0.2000	9.0900	0.1100	16
0.1	0.1	9000	1000	10000	-0.1000	0.3111	-0.1111	2.6995	0.4116	17
0.4	0.1	9000	1000	10000	-0.0667	0.5778	-0.1111	8.4699	0.1968	18
0.6	0.1	9000	1000	10000	-0.0444	0.7556	-0.1111	16.8516	0.1484	19
0.8	0.1	9000	1000	10000	-0.0222	0.9333	-0.1111	41.8806	0.1194	20
0.1	0.1	1000	9000	10000	-8.1000	10.0000	-1.0000	1.1248	0.1098	21
0.4	0.1	1000	9000	10000	-5.4000	10.0000	-1.0000	1.7458	0.1061	22
0.6	0.1	1000	9000	10000	-3.6000	10.0000	-1.0000	2.6739	0.1039	23
0.8	0.1	1000	9000	10000	-1.8000	10.0000	-1.0000	5.4537	0.1019	24
0.1	0.25	5000	5000	10000	-0.9000	1.5500	-0.5000	1.2923	0.4299	25
0.4	0.25	5000	5000	10000	-0.6000	1.7000	-0.5000	2.5000	0.3333	26
0.6	0.25	5000	5000	10000	-0.4000	1.8000	-0.5000	4.2026	0.2974	27
0.8	0.25	5000	5000	10000	-0.2000	1.9000	-0.5000	9.2291	0.2709	28
0.1	0.25	9000	1000	10000	-0.1000	0.4611	-0.2778	3.8986	0.7125	29
0.4	0.25	9000	1000	10000	-0.0667	0.6778	-0.2778	9.7388	0.4278	30
0.6	0.25	9000	1000	10000	-0.0444	0.8222	-0.2778	18.1558	0.3442	31
0.8	0.25	9000	1000	10000	-0.0222	0.9667	-0.2778	43.2107	0.2893	32
0.1	0.25	1000	9000	10000	-8.1000	11.3500	-2.5000	1.1275	0.2737	33
0.4	0.25	1000	9000	10000	-5.4000	10.9000	-2.5000	1.7547	0.2638	34
0.6	0.25	1000	9000	10000	-3.6000	10.6000	-2.5000	2.6859	0.2586	35
0.8	0.25	1000	9000	10000	-1.8000	10.3000	-2.5000	5.4682	0.2540	36
0.1	0.5	5000	5000	10000	-0.9000	2.0000	-1.0000	1.4625	0.7597	37
0.4	0.5	5000	5000	10000	-0.6000	2.0000	-1.0000	2.7208	0.6126	38
0.6	0.5	5000	5000	10000	-0.4000	2.0000	-1.0000	4.4365	0.5635	39
0.8	0.5	5000	5000	10000	-0.2000	2.0000	-1.0000	9.4721	0.5279	40
0.1	0.5	9000	1000	10000	-0.1000	0.7111	-0.5556	6.2176	0.8935	41

0.4	0.5	9000	1000	10000	-0.0667	0.8444	-0.5556	11.9705	0.6962	42
0.6	0.5	9000	1000	10000	-0.0444	0.9333	-0.5556	20.3869	0.6131	43
0.8	0.5	9000	1000	10000	-0.0222	1.0222	-0.5556	45.4499	0.5501	44
0.1	0.5	1000	9000	10000	-8.1000	13.6000	-5.0000	1.1353	0.5437	45
0.4	0.5	1000	9000	10000	-5.4000	12.4000	-5.0000	1.7745	0.5218	46
0.6	0.5	1000	9000	10000	-3.6000	11.6000	-5.0000	2.7097	0.5126	47
0.8	0.5	1000	9000	10000	-1.8000	10.8000	-5.0000	5.4944	0.5056	48
0.1	0.75	5000	5000	10000	-0.9000	2.4500	-1.5000	1.7923	0.9299	49
0.4	0.75	5000	5000	10000	-0.6000	2.3000	-1.5000	3.0000	0.8333	50
0.6	0.75	5000	5000	10000	-0.4000	2.2000	-1.5000	4.7026	0.7974	51
0.8	0.75	5000	5000	10000	-0.2000	2.1000	-1.5000	9.7291	0.7709	52
0.1	0.75	9000	1000	10000	-0.1000	0.9611	-0.8333	8.6474	0.9637	53
0.4	0.75	9000	1000	10000	-0.0667	1.0111	-0.8333	14.2921	0.8746	54
0.6	0.75	9000	1000	10000	-0.0444	1.0444	-0.8333	22.6730	0.8270	55
0.8	0.75	9000	1000	10000	-0.0222	1.0778	-0.8333	47.7141	0.7859	56
0.1	0.75	1000	9000	10000	-8.1000	15.8500	-7.5000	1.1554	0.8014	57
0.4	0.75	1000	9000	10000	-5.4000	13.9000	-7.5000	1.8043	0.7698	58
0.6	0.75	1000	9000	10000	-3.6000	12.6000	-7.5000	2.7395	0.7605	59
0.8	0.75	1000	9000	10000	-1.8000	11.3000	-7.5000	5.5234	0.7544	60
0.1	1	5000	5000	10000	-0.9000	2.9000	-2.0000	2.2222	1.0000	61
0.4	1	5000	5000	10000	-0.6000	2.6000	-2.0000	3.3333	1.0000	62
0.6	1	5000	5000	10000	-0.4000	2.4000	-2.0000	5.0000	1.0000	63
0.8	1	5000	5000	10000	-0.2000	2.2000	-2.0000	10.0000	1.0000	64
0.1	1	9000	1000	10000	-0.1000	1.2111	-1.1111	11.1111	1.0000	65
0.4	1	9000	1000	10000	-0.0667	1.1778	-1.1111	16.6667	1.0000	66
0.6	1	9000	1000	10000	-0.0444	1.1556	-1.1111	25.0000	1.0000	67
0.8	1	9000	1000	10000	-0.0222	1.1333	-1.1111	50.0000	1.0000	68
0.1	1	1000	9000	10000	-8.1000	18.1000	-10.0000	1.2346	1.0000	69
0.4	1	1000	9000	10000	-5.4000	15.4000	-10.0000	1.8519	1.0000	70
0.6	1	1000	9000	10000	-3.6000	13.6000	-10.0000	2.7778	1.0000	71
0.8	1	1000	9000	10000	-1.8000	11.8000	-10.0000	5.5556	1.0000	72
1.1	0	5000	5000	10000	0.1000	2.1000	0.0000	-21.0000	0.0000	73
1.2	0	5000	5000	10000	0.2000	2.2000	0.0000	-11.0000	0.0000	74
3	0	5000	5000	10000	2.0000	4.0000	0.0000	-2.0000	0.0000	75
5	0	5000	5000	10000	4.0000	6.0000	0.0000	-1.5000	0.0000	76
1.1	0	9000	1000	10000	0.0111	1.2111	0.0000	-109.0000	0.0000	77
1.2	0	9000	1000	10000	0.0222	1.3111	0.0000	-59.0000	0.0000	78
3	0	9000	1000	10000	0.2222	3.1111	0.0000	-14.0000	0.0000	79
5	0	9000	1000	10000	0.4444	5.1111	0.0000	-11.5000	0.0000	80
1.1	0	1000	9000	10000	0.9000	10.1000	0.0000	-11.2222	0.0000	81
1.2	0	1000	9000	10000	1.8000	10.2000	0.0000	-5.6667	0.0000	82
3	0	1000	9000	10000	18.0000	12.0000	0.0000	-0.6667	0.0000	83
5	0	1000	9000	10000	36.0000	14.0000	0.0000	-0.3889	0.0000	84

1.1	0.1	5000	5000	10000	0.1000	2.0800	-0.2000	-20.8957	0.0957	85
1.2	0.1	5000	5000	10000	0.2000	2.1600	-0.2000	-10.8918	0.0918	86
3	0.1	5000	5000	10000	2.0000	3.6000	-0.2000	-1.8539	0.0539	87
5	0.1	5000	5000	10000	4.0000	5.2000	-0.2000	-1.3374	0.0374	88
1.1	0.1	9000	1000	10000	0.0111	1.2000	-0.1111	-108.0925	0.0925	89
1.2	0.1	9000	1000	10000	0.0222	1.2889	-0.1111	-58.0861	0.0861	90
3	0.1	9000	1000	10000	0.2222	2.8889	-0.1111	-13.0383	0.0383	91
5	0.1	9000	1000	10000	0.4444	4.6667	-0.1111	-10.5238	0.0238	92
1.1	0.1	1000	9000	10000	0.9000	10.0000	-1.0000	-11.2102	0.0991	93
1.2	0.1	1000	9000	10000	1.8000	10.0000	-1.0000	-5.6538	0.0983	94
3	0.1	1000	9000	10000	18.0000	10.0000	-1.0000	-0.6421	0.0865	95
5	0.1	1000	9000	10000	36.0000	10.0000	-1.0000	-0.3558	0.0781	96
1.1	0.25	5000	5000	10000	0.1000	2.0500	-0.5000	-20.7411	0.2411	97
1.2	0.25	5000	5000	10000	0.2000	2.1000	-0.5000	-10.7329	0.2329	98
3	0.25	5000	5000	10000	2.0000	3.0000	-0.5000	-1.6514	0.1514	99
5	0.25	5000	5000	10000	4.0000	4.0000	-0.5000	-1.1124	0.1124	100
1.1	0.25	9000	1000	10000	0.0111	1.1833	-0.2778	-106.7342	0.2342	101
1.2	0.25	9000	1000	10000	0.0222	1.2556	-0.2778	-56.7204	0.2204	102
3	0.25	9000	1000	10000	0.2222	2.5556	-0.2778	-11.6077	0.1077	103
5	0.25	9000	1000	10000	0.4444	4.0000	-0.2778	-9.0689	0.0689	104
1.1	0.25	1000	9000	10000	0.9000	9.8500	-2.5000	-11.1926	0.2482	105
1.2	0.25	1000	9000	10000	1.8000	9.7000	-2.5000	-5.6353	0.2465	106
3	0.25	1000	9000	10000	18.0000	7.0000	-2.5000	-0.6148	0.2259	107
5	0.25	1000	9000	10000	36.0000	4.0000	-2.5000	-0.3249	0.2138	108
1.1	0.5	5000	5000	10000	0.1000	2.0000	-1.0000	-20.4881	0.4881	109
1.2	0.5	5000	5000	10000	0.2000	2.0000	-1.0000	-10.4772	0.4772	110
3	0.5	5000	5000	10000	2.0000	2.0000	-1.0000	-1.3660	0.3660	111
5	0.5	5000	5000	10000	4.0000	2.0000	-1.0000	-0.8090	0.3090	112
1.1	0.5	9000	1000	10000	0.0111	1.1556	-0.5556	-104.4786	0.4786	113
1.2	0.5	9000	1000	10000	0.0222	1.2000	-0.5556	-54.4591	0.4591	114
3	0.5	9000	1000	10000	0.2222	2.0000	-0.5556	-9.2697	0.2697	115
5	0.5	9000	1000	10000	0.4444	2.8889	-0.5556	-6.6869	0.1869	116
1.1	0.5	1000	9000	10000	0.9000	9.6000	-5.0000	-11.1643	0.4976	117
1.2	0.5	1000	9000	10000	1.8000	9.2000	-5.0000	-5.6066	0.4955	118
3	0.5	1000	9000	10000	18.0000	2.0000	-5.0000	-0.5855	0.4744	119
5	0.5	1000	9000	10000	36.0000	-6.0000	-5.0000	-0.2985	0.4652	120
1.1	0.75	5000	5000	10000	0.1000	1.9500	-1.5000	-20.2411	0.7411	121
1.2	0.75	5000	5000	10000	0.2000	1.9000	-1.5000	-10.2329	0.7329	122
3	0.75	5000	5000	10000	2.0000	1.0000	-1.5000	-1.1514	0.6514	123
5	0.75	5000	5000	10000	4.0000	0.0000	-1.5000	-0.6124	0.6124	124
1.1	0.75	9000	1000	10000	0.0111	1.1278	-0.8333	-102.2336	0.7336	125
1.2	0.75	9000	1000	10000	0.0222	1.1444	-0.8333	-52.2181	0.7181	126
3	0.75	9000	1000	10000	0.2222	1.4444	-0.8333	-7.0332	0.5332	127

5	0.75	9000	1000	10000	0.4444	1.7778	-0.8333	-4.4238	0.4238	128
1.1	0.75	1000	9000	10000	0.9000	9.3500	-7.5000	-11.1371	0.7482	129
1.2	0.75	1000	9000	10000	1.8000	8.7000	-7.5000	-5.5800	0.7467	130
3	0.75	1000	9000	10000	18.0000	-3.0000	-7.5000	-0.5675	0.7342	131
5	0.75	1000	9000	10000	36.0000	-16.0000	-7.5000	-0.2854	0.7299	132
1.1	1	5000	5000	10000	0.1000	1.9000	-2.0000	-20.0000	1.0000	133
1.2	1	5000	5000	10000	0.2000	1.8000	-2.0000	-10.0000	1.0000	134
3	1	5000	5000	10000	2.0000	0.0000	-2.0000	-1.0000	1.0000	135
5	1	5000	5000	10000	4.0000	-2.0000	-2.0000	-0.5000	1.0000	136
1.1	1	9000	1000	10000	0.0111	1.1000	-1.1111	-100.0000	1.0000	137
1.2	1	9000	1000	10000	0.0222	1.0889	-1.1111	-50.0000	1.0000	138
3	1	9000	1000	10000	0.2222	0.8889	-1.1111	-5.0000	1.0000	139
5	1	9000	1000	10000	0.4444	0.6667	-1.1111	-2.5000	1.0000	140
1.1	1	1000	9000	10000	0.9000	9.1000	-10.0000	-11.1111	1.0000	141
1.2	1	1000	9000	10000	1.8000	8.2000	-10.0000	-5.5556	1.0000	142
3	1	1000	9000	10000	18.0000	-8.0000	-10.0000	-0.5556	1.0000	143
5	1	1000	9000	10000	36.0000	-26.0000	-10.0000	-0.2778	1.0000	144

Table S3. Control sample results for Lin's Concordance Correlation Coefficient for CaseControl_AF, CaseControl_SE, and bias corrected CaseControl_SE between true and estimated MAF

					Lin's CCC		
**Trait**	**Cases**	**Controls**	**Bin**	**N Variants**	**CCAFE AF**	**CCAFE SE**	**CCAFE SE Corrected**
Prostate Cancer	79148	61106	[0.0, 0.1]	18	1	0.90315	NA*
			(0.1, 0.2]	27	1	0.73818	NA
			(0.2, 0.3]	35	1	0.50244	NA
			(0.3, 0.4]	32	1	0.24222	NA
			(0.4, 0.5]	36	1	0.03586	NA
PanUKBB Diabetes	16550	403923	[0.0, 0.1]	3721679	1	0.9700	0.9651
			(0.1, 0.2]	1766962	1	0.7338	0.9802
			(0.2, 0.3]	1355742	1	0.3972	0.9389
			(0.3, 0.4]	1203911	1	0.1428	0.8214
			(0.4, 0.5]	1130270	1	0.0213	0.2060
PanUKBB Diabetes	668	5956	[0.0, 0.1]	3453818	0.99998	0.9567	0.9760
			(0.1, 0.2]	1871888	0.99995	0.7097	0.8850
			(0.2, 0.3]	1487374	0.99994	0.4129	0.7449
			(0.3, 0.4]	1242683	0.99993	0.1651	0.4508
			(0.4, 0.5]	1126168	0.99992	0.0281	0.2175

* Prostate cancer MAFs were not corrected due to small number of overlapping variants with gnomAD

Table S4. Effect of rounding for CaseControl_SE. Lin's Concordance Correlation Coefficient (CCC) for known vs estimated case and control AFs***.***

**Case**	**Number of Decimal Places Rounded**			
**bin**	**5**	**4**	**3**	**2**
[0,0.1]	0.9843	0.9842	0.9842	0.9711
(0.1,0.2]	0.7332	0.7329	0.7359	0.1376
(0.2,0.3]	0.3946	0.3936	0.4316	0.0134
(0.3,0.4]	0.1377	0.1383	0.2317	0.0000
(0.4,0.5]	0.0215	0.0214	0.0026	0.0000
**Control**	**Number of Decimal Places Rounded**			
**bin**	**5**	**4**	**3**	**2**
[0,0.1]	0.9803	0.9803	0.9803	0.9677
(0.1,0.2]	0.7347	0.7344	0.7375	0.1386
(0.2,0.3]	0.3964	0.3954	0.4332	0.0136
(0.3,0.4]	0.1407	0.1413	0.2350	0.0007
(0.4,0.5]	0.0216	0.0214	0.0027	0.0008

Table S5. Effect of rounding for CaseControl_AF. Lin's Concordance Correlation Coefficient (CCC) for known vs estimated case and control AFs***.***

**Case**	**Number of Decimal Places Rounded**			
**bin**	**5**	**4**	**3**	**2**
[0,0.1]	1.0000	1.0000	0.9999	0.9970
(0.1,0.2]	0.9999	0.9999	0.9999	0.9951
(0.2,0.3]	0.9999	0.9999	0.9998	0.9950
(0.3,0.4]	0.9999	0.9999	0.9998	0.9950
(0.4,0.5]	0.9999	0.9999	0.9998	0.9950
**Control**	**Number of Decimal Places Rounded**			
**bin**	**5**	**4**	**3**	**2**
[0,0.1]	1.0000	1.0000	1.0000	0.9971
(0.1,0.2]	1.0000	1.0000	1.0000	0.9952
(0.2,0.3]	1.0000	1.0000	1.0000	0.9951
(0.3,0.4]	1.0000	1.0000	1.0000	0.9952
(0.4,0.5]	1.0000	1.0000	1.0000	0.9951

* rounding to 6-9 decimal places was the same as five
