# Supplementary material for: CCAFE: Estimating case and control allele frequencies from GWAS summary statistics
Source: HGG Adv. 2026 Apr 20;7(3):100616. doi: 10.1016/j.xhgg.2026.100616 (PMC13347939; doi:10.1016/j.xhgg.2026.100616)
Supplement: Document S2. Article plus supplemental information [file mmc2.pdf]

HGG Advances

ARTICLE

CCAFE: Estimating case and control allele frequencies from GWAS summary statistics

Hayley R. Stoneman,[1,2,6,*] Hugo Lemus Gomez,[1,3] Adelle Price,[1,3] Christopher R. Gignoux,[1,2,4] and Audrey E. Hendricks[1,2,3,4,5]

Summary

Genetic summary statistics can be used in a variety of analyses, such as causal inference, genetic correlation, and risk scores, to provide insights into the genetic architecture of conditions and traits. However, complete statistics are often not reported, limiting the utility of these data. Indeed, many post hoc analyses of diseases require case and control allele frequencies (AFs), which are not always published. Here, we present methods and software to derive case and control AFs from genome-wide association study (GWAS) summary statistics using the odds ratio, case and control sample sizes, and either the total (case and control aggregated) AF or the standard error (SE). In simulations and real data, derivations of case and control AFs using total AFs are highly accurate, whereas using SE underestimates AFs when covariates were included in the GWAS. While estimating case and control AFs using the total AF is preferred due to its high accuracy, the SE is more commonly available. Thus, we developed a bias adjustment using gnomAD AFs as a proxy for true AFs, reducing bias when using the SE. The methods and software provided here expand the utility of publicly available genetic summary statistics and promote the reusability of genomic data. The R package Case-Control Allele Frequency Estimation (CCAFE) is freely available on Bioconductor and GitHub.

Introduction

Growth in genomics research has rapidly increased the number of genome-wide association studies (GWASs), which are often made publicly available through summary statistics to ease storage and privacy concerns. This increase in summary-level data has catalyzed our understanding of the relationship between genetics and health and disease through the development and application of methods such as GWAS meta-analysis,[1] polygenic risk scores (PRSs),[2,3] Mendelian randomization (MR),[4] and external common controls.[5–7] GWAS summary statistics often include a subset of odds ratios (ORs) or effect sizes (beta), standard errors (SEs), *p* values, allele frequencies (AFs), and sample sizes. However, there is a lack of standardization in reporting summary statistics, in both content and format, presenting challenges for data reuse.[8–10] In 2021, a study of 327 previously curated, publicly available summary statistics files found over 100 unique formats[11] resulting from different types of traits studied (i.e., binary or quantitative), the software used for analysis, or simply the summary statistics that the author chose to include in the final summary file. While movements have been made to standardize summary statistics reporting,[8,12] inconsistency in reporting, including missing case and control AFs, can limit and sometimes fully hinder the use of these data for further studies.

In an assessment of the 2021 requirements for submission to the GWAS catalog, Hayhurst et al. found that over 50% of studies submitted between January 2020 and July 2022 were missing at least one mandatory field.[13] Notably, the most commonly missing fields were effect AFs (>40% missing) and SEs (>25% missing)—although SEs can be recapitulated from the effect estimate and *p* value, which are more commonly included (0% and <20% missing, respectively). Inclusion of both case and control AFs was even more rare. Further, clear standards for reporting the effect AFs do not exist, with researchers frequently reporting the total sample AF, resulting in the aggregation of case and control samples. Without access to case- and control-specific AFs, secondary uses for summary data such as case-case GWASs, group PRSs,[14] and external controls may not be possible. The implementation of standards for the content and format of GWAS summary statistics has the potential to greatly increase the number of usable datasets for downstream analyses in the future. However, the close to 90,000 available studies in the GWAS catalog as of March 2025 are not likely to be updated, limiting the utility of a vast amount of existing genetic summary data.

In 2022, Yang et al. presented a framework to reconstruct GWAS case and control AFs using case and control sample sizes, ORs, and SEs as part of a meta-analysis software for the reconstruction of allelic and genotypic

[1]Department of Biomedical Informatics, University of Colorado Anschutz Medical Campus, Aurora, CO 80045, USA; [2]Human Medical Genetics and Genomics Program, University of Colorado Anschutz Medical Campus, Aurora, CO 80045, USA; [3]Department of Mathematical and Statistical Sciences, University of Colorado Denver, Denver, CO 80204, USA; [4]Colorado Center for Personalized Medicine, University of Colorado Anschutz Medical Campus, Aurora, CO 80045, USA
[5]All editorial responsibility for this paper was handled by other members of the editorial board.
[6]Lead contact
*Correspondence: hay.bale@hotmail.com
https://doi.org/10.1016/j.xhgg.2026.100616.
© 2026 The Authors. Published by Elsevier Inc. on behalf of American Society of Human Genetics.
This is an open access article under the CC BY license (http://creativecommons.org/licenses/by/4.0/).

frequencies and counts (ReACt).[14] While Yang et al. used the derived case and control AFs in secondary analyses, such as case-case GWAS and meta-analysis, there was no evaluation of the derived case-control AFs and thus no understanding of the method's accuracy or performance to estimate case and control AFs. To address this gap, we performed a rigorous evaluation of this SE-based case and control AF derivation in simulation and real data. Upon finding biased performance, we further developed a bias adjustment for Yang et al.'s method. Additionally, estimation of case and control AFs is embedded within the ReACt software and is not available as an independent function, further limiting use. We provide a user-friendly, stand-alone function that implements this method (and our subsequent bias adjustment) within the Case-Control Allele Frequency Estimation (CCAFE) software.

As another option for estimating case and control AFs, we developed a computationally efficient method that uses the total AF (instead of the SE). In real data and simulations, we evaluated and compared this AF-based method with Yang et al.'s SE method with and without our bias adjustment.

We provide functions for all methods in the *CCAFE R* software package,[15] available on GitHub and Bioconductor.[16] CCAFE enables derivation of GWAS case and control AFs using the GWAS total sample AF or SE, number of cases and controls, and effect estimates, supporting broader use of GWAS summary statistics. These well-documented and user-friendly functions will help to expand the use of summary statistics, enabling novel applications of existing data.

Material and methods

Implementation

CaseControl_AF mathematical framework

We derive case and control AFs for a given variant i using the case and control sample sizes (n_{case} and $n_{control}$), OR_i, and total AF ($AF_{total,i}$). $AF_{total,i}$ and OR_i can be represented as shown in Equations 1 and 2.

$$AF_{total,i} = \frac{\left(n_{case}AF_{case,i} + n_{control}AF_{control,i}\right)}{n_{total}} \quad \text{(Equation 1)}$$

$$OR_i = \frac{a_i d_i}{b_i c_i} \quad \text{(Equation 2)}$$

Here, a, b, c, and d are the cells of a two-by-two contingency table representing the allele counts of the effect and non-effect, or alternate and reference, alleles for cases and controls. These quantities can be calculated as shown in Equations 3, 4, 5, and 6.

$$a_i = 2n_{case} * AF_{case,i} \quad \text{(Equation 3)}$$

$$b_i = 2n_{case}\left(1 - AF_{case,i}\right) \quad \text{(Equation 4)}$$

$$c_i = 2n_{control} * AF_{control,i} \quad \text{(Equation 5)}$$

$$d_i = 2n_{control}\left(1 - AF_{control,i}\right) \quad \text{(Equation 6)}$$

Substituting Equations 3, 4, 5, and 6 into the OR equation in Equation 2, we arrive at Equation 7.

$$OR_i = \frac{AF_{case,i}\left(1 - AF_{control,i}\right)}{\left(1 - AF_{case,i}\right)AF_{control,i}} \quad \text{(Equation 7)}$$

We can then use Equation 1 to solve for $AF_{case,i}$ (shown in Equation 8) and substitute it into Equation 7, which results in a quadratic as shown in Equation 9.

$$AF_{case,i} = \frac{n_{total}}{n_{case}}AF_{total,i} - \frac{n_{control}}{n_{case}}AF_{control,i} \quad \text{(Equation 8)}$$

$$AF^2_{control,i}\left[\frac{n_{control}}{n_{case}}(OR_i - 1)\right] + AF_{control,i}\left[OR_i\left(1 - \left(\frac{n_{total}}{n_{case}}AF_{total,i}\right)\right) + \frac{1}{n_{case}}\left(n_{control} + n_{total}AF_{total,i}\right)\right] - \frac{n_{total}}{n_{case}}AF_{total,i} = 0 \quad \text{(Equation 9)}$$

We solve for the quadratic roots and choose $AF_{control,i}$ to be the root between 0 and 1. The full derivation is shown in Note S1. Additionally, we show there is only one solution for $AF_{control,i}$ that falls between the bounds of 0 and 1 (Notes S2 and S3; Figure S1; Tables S1 and S2). $AF_{case,i}$ is then estimated using Equation 8.

CaseControl_SE mathematical framework

The full derivation for the case and control AFs using SE can be found in the supplemental information of the original publication by Yang et al. Briefly, the method relies on allele counts a, b, c, and d as shown in Equations 3, 4, 5, and 6. Since this results in four unknown quantities, four equations are used to solve the system. These four unknown quantities are related to the SE, the sample size of cases and controls, and the OR. Ultimately, Yang et al. solve for d and use the resulting quadratic to solve for the allele counts and then the AFs. To fulfill the assumption that the solution is between [0, 1], Yang et al. use the minor AF (MAF), resulting in a loss of connection with an allele. While the minor and major alleles can likely be accurately inferred when the MAF is very different from 0.5, accurate inference of the minor allele becomes difficult as the MAF approaches 0.5. Notably, the resulting quadratic solved in this framework utilizes the total allele number (AN; i.e., 2*n for autosomes) in cases and controls rather than the AF. This results in the need for sex-chromosome-specific implementations and thus requires information on the number of X and Y chromosomes in case and control samples.

CaseControl_SE bias correction

To reduce bias in the estimation of case and control AFs using the SE, we present a bias adjustment method. As the bias between the estimated AF and true AF was observed to be a curve, we tested a variety of appropriate models, finding the second-order polynomial to fit best. Because the bias trends with the MAF bin, we fit regression models by MAF bin. We evaluated using 5% vs. 10% MAF bins and observed nearly identical least squares (LS) difference. We therefore chose 10% MAF bins for computational efficiency. We recommend 10,000 variants per MAF bin in the proxy dataset for optimal bias correction (Note S4; Figures S2 and S3). For the implementation here, we fit a second-order polynomial

where the outcome is the estimated MAF from the SE method and the predictor is the gnomAD MAF (Equation 10) for each MAF bin ([0, 0.1) [0.1, 0.2) [0.2, 0.3) [0.3, 0.4) [0.4, 0.5]) (Figure S4). We identified and excluded outliers defined as observations with an absolute value of the studentized residual >3 and refit the models.

The bias adjustment algorithm is shown below.

$$\widehat{MAF}_{CaseControl\_SE} = \hat{a} * MAF^2_{gnomAD} + \hat{b} * MAF_{gnomAD} + \hat{c} \qquad \text{(Equation 10)}$$

The bias can then be estimated as

$$\widehat{bias} = MAF_{gnomAD} - \widehat{MAF}_{CaseControl_{SE}}. \qquad \text{(Equation 11)}$$

To complete the bias adjustment for variants not in the proxy data, the following steps are used.

(1) Polynomial regression (second order) is fit using the proxy data (MAF_{gnomAD}) as the predictor and the CaseControl_SE MAF ($MAF_{CaseControl\_SE}$) as the outcome by gnomAD MAF bin, resulting in estimates for $\hat{a}, \hat{b}$, and $\hat{c}$.

(2) For variants not in the proxy dataset (i.e., CaseControl_SE MAF, but unknown gnomAD MAF), solve for the estimated gnomAD MAF ($\widehat{MAF}_{gnomAD}$) using $\hat{a}, \hat{b}$, and $\hat{c}$.

$$0 = \hat{a} * MAF^2_{gnomAD} + \hat{b} * MAF_{gnomAD} + \left(\hat{c} - MAF_{CaseControl\_SE}\right) \qquad \text{(Equation 12)}$$

(3) Calculate $\widehat{MAF}_{CaseControl\_SE}$.

$$\widehat{MAF}_{CaseControl\_SE} = \hat{a} * \widehat{MAF}^2_{gnomAD} + \hat{b} * \widehat{MAF}_{gnomAD} + \hat{c} \qquad \text{(Equation 13)}$$

(4) Calculate $\widehat{bias}$.

$$\widehat{bias} = \widehat{MAF}_{gnomAD} - \widehat{MAF}_{CaseControl\_SE} \qquad \text{(Equation 14)}$$

(5) Calculate $MAF^*_{CaseControl\_SE}$.

$$MAF^*_{CaseControl\_SE} = \widehat{MAF}_{CaseControl\_SE} + \widehat{bias} \qquad \text{(Equation 15)}$$

Implementation in R

We include the method from Yang et al. as a function, named CaseControl_SE, in our R package. As this method was originally embedded in a GWAS meta-analysis software developed in C, we replicated the results using their original software in C and our version in R to ensure complete and robust translation of the code (Note S5). We also include our framework as the function CaseControl_AF. Both methods (using either total AF or SE) rely on solving the roots of a quadratic. As we know that these quantities (AFs) exist and are real numbers, this can be done easily in R in closed form, ensuring scalability for large datasets. Both methods use the number of cases and controls, ORs, and either the total AF or SE as inputs. Note that the OR can be derived from the beta estimate, and the SE can be derived given the effect estimate and either the *p* value or the test statistic. To use the SE to derive the MAF for sex chromosomes, the user must also include the number of X and Y chromosomes per case and control sample.

Additionally, the user may obtain bias-corrected MAF estimates from CaseControl_SE by including a data frame with variants from the observed data and a proxy dataset, such as gnomAD. This data frame must contain variant information (chromosome and position) and the proxy MAF (e.g., from gnomAD) and be merged so that the AF matches the same allele. As the bias correction can be estimated by a subset of variants, this data frame does not need to contain all variants in the observed dataset, which helps ensure more efficient computation. The adjusted MAFs (total, case, and control) are appended as three additional columns to the original data input.

Simulation study

We used the R package *PhenotypeSimulator*[17] to simulate models with genotypes for 10,000 variants for one binary phenotype and zero, one, or three covariates (Figure S5). *PhenotypeSimulator* generates phenotypes as the sum of genetic variant effects, covariate effects, and observational noise. We simulated multiple sample sizes (n = 1,000, 6,000, 10,000, 50,000, and 100,000) with equal numbers of cases and controls, as well as two imbalanced sample sizes (n = 6,000: 600 cases and 5,400 controls emulating the Pan-UK Biobank (Pan-UKBB) African Diabetes sample; n = 50,000: 1,200 cases and 48,800 controls). When including one covariate, a binary variable, such as sex, was simulated with a probability of 0.5. For the three covariate simulation scenarios, two additional variables were simulated: one categorical variable, such as educational attainment or geographical region (five categories), and a continuous variable, e.g., similar to age.

The binary phenotype was simulated with *PhenotypeSimulator* using a binomial distribution with the probability determined by the proportion of variance explained for causal variants (genVar). The additional parameters of the simulation model split the remaining variance (i.e., 1 – genVar) proportionally: kinship (eta), correlated covariates (rho), uncorrelated covariates (delta), and random noise (phi). Non-causal variants have an effect size of zero by default. For our simulations, we simulated the proportion variance explained by causal variants (genVar) as 0.8, leaving 0.2 leftover, which was split 0.6 for uncorrelated covariates (delta) and 0.4 for random noise (phi). In other words, $0.6 \times 0.2 = 0.12$ and $0.4 \times 0.2 = 0.08$ of the variance was explained by uncorrelated covariates and random noise, respectively. In the initial simulations, we did not simulate correlated covariates or relatedness (i.e., eta = 0). We simulated 100 causal variants and 9,900 non-causal variants. The 9,900 non-causal variants were simulated using the same framework, with the proportion of variance explained only by covariates. More information can be found in the *PhenotypeSimulator* publication[17] and the CRAN manual (https://cran.r-project.org/web/packages/PhenotypeSimulator/index.html).

The simulated genotype, phenotype, and covariate data were then used to fit a logistic regression model from which the OR and SE for each genetic variant were obtained. Using the logistic regression summary statistics (e.g., OR and SE) in CCAFE, the case and control AFs were estimated and compared to the true calculated values from the case and control samples assessed using Lin's concordance correlation coefficient (CCC)[18] (additional details are provided in data and code availability). Lin's CCC measures the concordance between two measures of the same variable (here, the estimated AF and true AF), with perfect agreement at 1. To assess the bias correction framework, we simulated proxy MAFs by adding a small amount of random noise (uniform distribution from −0.075 to 0.075) to the simulated total MAFs for both non-causal and causal variants.

Table 1. Lin's concordance correlation coefficient in simulations

				Lin's CCC			
				Cases		Controls	
Cases	Controls	Total	Covariates	CCAFE AF	CCAFE SE	CCAFE AF	CCAFE SE
500	500	1,000	0	0.9916	0.9811	0.9917	0.9811
			1	0.9905	0.7430	0.9914	0.7420
			3	0.9916	0.6913	0.9905	0.6899
3,000	3,000	6,000	0	0.9982	0.9952	0.9983	0.9951
			1	0.9981	0.8457	0.9980	0.8442
			3	0.9981	0.6690	0.9982	0.6690
5,000	5,000	10,000	0	0.9988	0.9967	0.9988	0.9966
			1	0.9987	0.8607	0.9988	0.8599
			3	0.9986	0.6747	0.9986	0.6747
25,000	25,000	50,000	0	0.9995	0.9989	0.9995	0.9989
			1	0.9993	0.8641	0.9993	0.8643
			3	0.9994	0.6806	0.9993	0.6808
50,000	50,000	100,000	0	0.9995	0.9993	0.9995	0.9993
			1	0.9995	0.7589	0.9994	0.7593
			3	0.9995	0.6761	0.9995	0.6754
600	5,400	6,000	0	0.9921	0.9941	0.9980	0.9882
			1	0.9916	0.8410	0.9980	0.8369
			3	0.9918	0.8234	0.9980	0.8189
1,200	48,800	50,000	0	0.9956	0.9992	0.9994	0.9955
			1	0.9954	0.9903	0.9994	0.9868
			3	0.9954	0.9773	0.9994	0.9731

All simulations contained 10,000 variants (100 causal). AF and SE columns contain Lin's CCC between estimated and true case and control AFs for CaseControl_AF and CaseControl_SE, respectively.

To evaluate the methods in the presence of population stratification, we simulated population-specific AFs for two groups using gnomAD African (AFR-like) and European (EUR-like) AFs. We combined the AFs, weighting 80% AFR-like and 20% EUR-like. For a random subset of 10% ($N = 1{,}000$) of variants, we only used the EUR-like AF. We simulated the two reference groups to have different case probabilities (1.5× and 1× for AFR-like and EUR-like, respectively), thus creating confounding due to population stratification. Simulation of the phenotype and three covariates proceeded as previously described. The first 10 principal components (PCs) were estimated using the *stats* R package and were used for the adjusted regressions. We also tested the effect of relatedness by including kinship in *PhenotypeSimulator* (eta = 0.6), which allows part of the genetic variance to be explained by relatedness between individuals rather than causal SNPs.

Genotype dosage can help account for uncertainty in genotype calling. Thus, we evaluated a scenario in which the OR may be estimated from genotype dosage while the AF is estimated from genotype calls. We simulated AC for bi-allelic variants (i.e., 0, 1, or 2) for 10,000 variants in 10,000 individuals using *rmultinom* in R with a p equal to the AF. We added a small (± 0.1) or moderate (± 0.2) amount of random noise using a uniform distribution to each genotype. We then assessed concordance in AFs calculated using the true genotypes without added uncertainty, genotype dosages with added uncertainty, and genotype calls obtained by rounding genotype dosages.

Real data application

Publicly available datasets

We tested the ability of the methods to reconstruct case and control AFs from real datasets for which case and control AFs were publicly available (Table 1). These datasets had a range of case and control sample sizes, numbers of genetic variants, and covariates included in the original GWAS. We used 148 variants from a 2018 prostate cancer PGS (https://www.ebi.ac.uk/gwas/studies/GCST006085), which was generated from data collected for a GWAS of 79,148 cases and 61,106 controls.[19] This GWAS published control AFs, which were used with the OR to derive the case AFs (Note S6). Additionally, we use the Pan-UKBB GWAS summary statistics for diabetes in EUR and AFR samples (https://pan-dev.ukbb.broadinstitute.org/docs/per-phenotype-files/index.html).[20] Here 9,178,564 genome-wide variants were available. The Pan-UKBB EUR GWAS contained 16,550 cases and 403,923 controls, while the Pan-UKBB AFR GWAS contained 668 cases and 5,956 controls. We also used the Pan-UKBB EUR

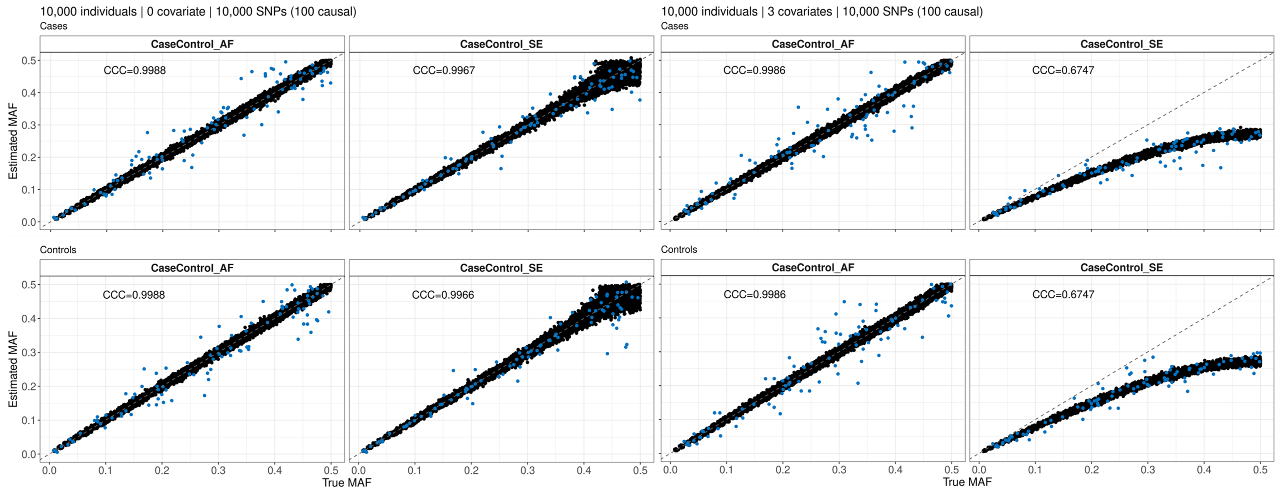

Figure 1. Estimated case and control AFs from summary statistics
Simulated genotypes and phenotypes were generated using the *PhenotypeSimulator* R package. Genotypes for 10,000 variants, of which 100 were causal (shown in blue), were generated for 5,000 cases and 5,000 controls. Logistic regression was used along with 0 (A) or 3 (B) covariates to generate per-variant summary statistics. CaseControl_AF and CaseControl_SE methods were used to estimate the case and control AFs. When covariates were included in the original GWAS, bias increased as MAF increased for CaseControl_SE, resulting in a systematic underestimation of MAFs (B). CaseControl_AF was accurate across the MAF spectrum, regardless of whether covariates were included. Lin's CCC is shown between the true simulated MAF and the estimated MAF.

GWAS data to evaluate the effect of summary statistic rounding. As the AF usually has more significant digits reported, we evaluated Lin's CCC with rounding from nine to two decimal places for AFs, whereas we evaluated five to two decimal places for SEs. We compared the estimated case and control AFs to the known AFs using Lin's CCC per MAF bin.

Correction using gnomAD as proxies

We applied our bias correction to the Pan-UKBB diabetes GWAS data (>9 million variants), filtering down to chromosome 1 biallelic variants from gnomAD as proxies (https://gnomad.broadinstitute.org/downloads). We lifted over Pan-UKBB from GRCh37 to GRCh38 using LiftOver and merged with gnomAD v.3.1.2,[21] retaining the intersection of variants. We removed variants where the allele pair did not match between the two datasets. We flipped informative alleles (e.g., A/G and C/T) and the AF so that the reference and alternate alleles were the same between datasets. Non-informative allele pairs (i.e., A/T and C/G) were removed. The final dataset contained 1,212,618 variants on chromosome 1. We used the gnomAD non-Finnish European (NFE) group as a proxy for total MAFs in the Pan-UKBB EUR sample and the gnomAD AFR/African American (AFR/AFRAM) group as a proxy for MAFs in the Pan-UKBB AFR sample. We then adjusted the estimated MAFs for all >9 million genome-wide variants in each sample.

Results

Simulation results

For CaseControl_SE, the previously published method that uses SE rather than total AF, we found an increase in the variance in AF estimates at higher MAFs (i.e., MAF > 0.4) (Figures 1 and S6). When simulated covariates were included in the regression to produce the summary statistics, CaseControl_SE also had increasing bias (average difference between the estimated and true MAF) as the MAF increased, with the MAF systematically underestimated (Figures 1 and S7–S10). Conversely, CaseControl_AF, the method introduced here that uses total AF, provided accurate estimates with small variation across the MAF spectrum in all tested scenarios, even with the presence of covariates (Figure S6). This was reinforced by the Lin's CCC between the true MAF and the estimated MAF, which decreased as the number of covariates increased for CaseControl_SE but remained near 1 for CaseControl_AF (Table 1). Notably for both methods, the estimates for some causal variants were variable compared to the estimates for the non-causal variants (Figures 1 and S6–S8). In addition, by evaluating AF-to-MAF transformations in the CaseControl_AF method (which can use either AF or MAF), we found that using the total MAF rather than the total AF consistently increased the variability of the estimates across the MAF spectrum (Figure S11).

We further assessed the effects of population stratification, relatedness, and genotype dosage. For both Control_AF and CaseControl_SE, we found that confounding due to population stratification (Figure S12) increased the variability of the AF estimates compared to simulations without confounding, whereas relatedness in the sample used to generate the summary statistics did not noticeably affect the results (Figure S13). Correcting for population stratification by including the first 10 PCs reduced the variability but not to the level seen without population stratification (Figure 1). While variability increased, the bias was not substantially affected in the presence of population stratification or relatedness. We found high concordance between the true AF, dosage AF, and genotype from dosage AF, even given the small and moderate uncertainty added to the latter two scenarios (Figure S14).

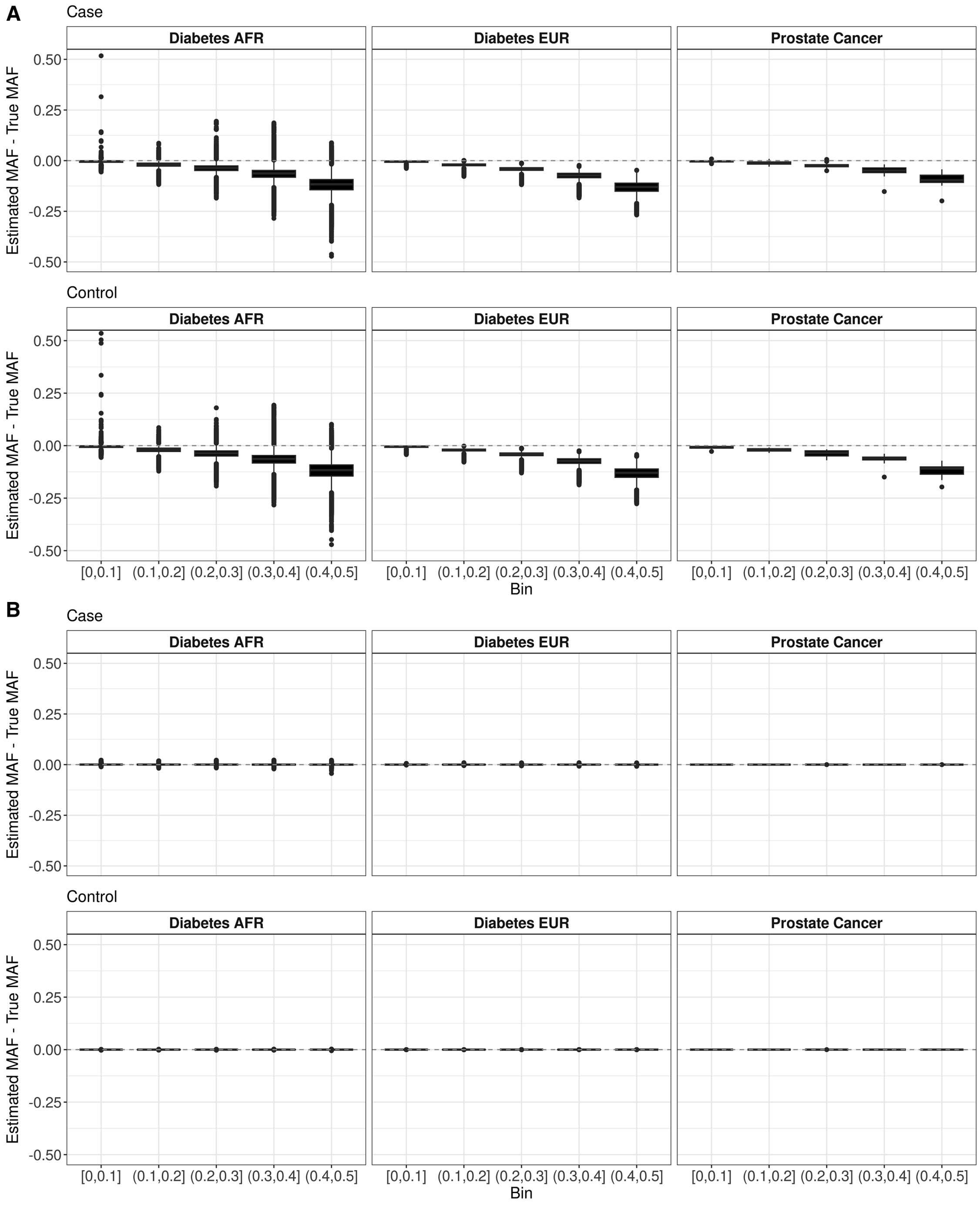

Figure 2. Comparison of case and control AF estimation in real datasets

Results of estimating case MAFs for six datasets with various sample sizes. The prostate cancer dataset ($n_{case} = 79{,}148$; $n_{control} = 61{,}106$) has 148 variants from a 2018 PRS. Diabetes EUR ($n_{case} = 16{,}550$; $n_{control} = 403{,}923$) and Diabetes AFR ($n_{case} = 668$; $n_{control} = 5{,}956$) contain >9 million variants from the PanUKBB GWAS. The lower and upper hinges of boxes correspond to the 25th and 75th percentiles, respectively. Upper and lower whiskers are the largest and smallest values no further than 1.5× the inter-quartile range (IQR) from the hinge, and the center line represents the median.

(A) CaseControl_SE, the method proposed in the ReACt software using SE, underestimates the true MAF, with bias increasing and precision (width of the boxplot) decreasing as the true MAF increases.

(B) CaseControl_AF, the framework developed here using total AF produces a highly accurate estimation of known AFs using CaseControl_AF, with some variability in the Diabetes AFR dataset, which has a smaller sample size.

Real data analysis

CaseControl_SE underestimated the true case and control AFs in all datasets, especially for variants with a higher MAF (Figure 2A). We identified the highest bias for variants with small SEs (i.e., SE < 0.02) (Figures S15 and S16). Variants with smaller SEs were often those

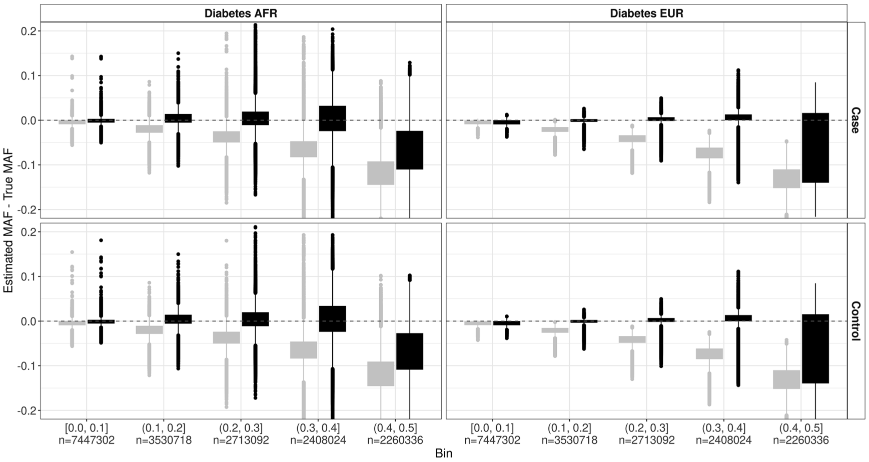

Figure 3. Correction mitigates bias in CaseControl_SE MAF estimates
We use our bias correction to adjust the case and control MAF estimates from CaseControl_SE for >9 million genome-wide variants from the AFR and EUR Pan-UKBB Diabetes datasets. To estimate the bias correction, we used >1.2 million variants on chromosome 1 that were harmonized between Pan-UKBB and gnomAD v.3.1.2. The gnomAD African/African American (AFR/AFRAM) group was used as a proxy for true MAFs for the AFR sample (left) and the gnomAD non-Finnish European (NFE) group was used as a proxy for true MAFs for the EUR sample (right). We see an improvement (i.e., less bias and greater Lin's CCC) when using the bias correction (gray; AFR CCC = 0.9877, EUR CCC = 0.9943) compared to the uncorrected CaseControl_SE MAF estimates (black; AFR CCC = 0.9369, EUR CCC = 0.9382). The lower and upper hinges of boxes correspond to the 25th and 75th percentiles, respectively. Upper and lower whiskers are the largest and smallest values no further than 1.5× the inter-quartile range (IQR) from the hinge, and the center line represents the median.

with a large MAF (e.g., MAF > 0.3). Additionally, as expected, the minimum SE was smaller when case sample sizes were larger, resulting in a positive correlation between bias and sample size. When using the SE to reconstruct the case and control AFs, the CCC was lower for higher MAF bins (i.e., CCC < 0.06 for MAF [0.4, 0.5] vs. CCC > 0.90 for MAF [0, 0.1)), and this pattern was particularly compounded for datasets with larger numbers of cases (Tables S2 and S3). Conversely, CaseControl_AF accurately reconstructed the case and control AFs in all datasets (Figure 2B), and the CCC of the estimated case and control AFs with the true AFs remained at or near 1 (Tables S2 and S3). Rounding the SE to three or more decimal places produced no notable decrease in accuracy; however, rounding to two decimal places resulted in a decrease in accuracy for CaseControl_SE, especially for MAFs > 0.1 (Tables S4 and S5). While Lin's CCC also decreased when rounding AF to two decimal places, overall concordance was still high (>0.99).

CaseControl_SE bias correction

We developed a bias correction framework using gnomAD v.3.1.2 AFs as proxies for the true AFs. We tested our framework using the Pan-UKBB Diabetes datasets. The adjusted MAFs using the bias correction had less bias and a higher Lin's CCC for both the AFR and EUR samples than the unadjusted method (Figure 3; Table 2). The average CCC across all variants improved from 0.9369 to 0.9877 for the PanUKBB AFR diabetes GWAS and from 0.9382 to 0.9943 for the EUR sample. While the overall bias is much lower with the bias correction, we still observe an increase in bias and variance as the MAF increases, especially for higher MAF bins (Figure 3). Similar results were found in simulations (Figure S17). Notably, we observe similar bias in the causal vs. non-causal variants for both the bias-corrected and uncorrected estimates.

Discussion

Here, we present methods and software (CCAFE) to estimate case- and control-specific AFs from GWAS summary statistics. While our method using total AF outperforms the SE-based method and has lower bias and variability in its estimates, both methods allow flexibility based on what summary statistics are avaiable. This is especially important given the inconsistency in the summary statistics released in repositories such as the GWAS catalog, with the effect AF missing in >40% of them.[12] Indeed, SE is often more available and easily derivable (e.g., given the effect estimate and either a p value or test statistic) than total AF. To increase robust use of the SE method, we developed and provided a bias adjustment within the R package that greatly decreases the bias in the case and control AF estimates, which can be especially large for higher variants with higher MAFs.

In both simulations and real data, we show that CaseControl_AF provides highly accurate estimates of case and control AFs across a variety of sample sizes and in the presence of covariates. Conversely, while CaseControl_SE provides accurate AF estimates when no covariates are included in the original GWAS, it is biased when the original GWAS includes covariates. Since CaseControl_AF is much less biased and more precise, we recommend using CaseControl_AF when total AFs are available. We hypothesize that the bias in CaseControl_SE arises from an underestimation of the SE used for the derivation in the ReACt method when covariates are included in the original GWAS. Specifically, the ReACT method derivation assumes a simple linear regression with no covariates. However, most publicly available GWAS summary statistics are from models that adjust for covariates. Adjusting for covariates usually reduces the model's residual error and, consequently, the SE as well, resulting in a smaller SE than a simple

Table 2. Concordance of inferred AF in real GWAS datasets for cases

					Lin's CCC		
Trait	Cases	Controls	MAF bin	*N* variants	CCAFE AF	CCAFE SE	CCAFE SE corrected
Prostate cancer	79,148	61,106	[0.0, 0.1]	18	1	0.9643	N/A[a]
			(0.1, 0.2]	27	1	0.8560	N/A
			(0.2, 0.3]	35	1	0.6592	N/A
			(0.3, 0.4]	32	1	0.2401	N/A
			(0.4, 0.5]	36	1	0.0596	N/A
PanUKBB Diabetes EUR	16,550	403,923	[0.0, 0.1]	3,721,679	0.99998	0.9701	0.9651
			(0.1, 0.2]	1,766,962	0.99993	0.7346	0.9805
			(0.2, 0.3]	1,355,742	0.99989	0.3977	0.9404
			(0.3, 0.4]	1,203,911	0.99986	0.1425	0.8244
			(0.4, 0.5]	1,130,270	0.99985	0.0213	0.2022
PanUKBB Diabetes AFR	668	5,956	[0.0, 0.1]	3,453,818	0.99871	0.9624	0.9774
			(0.1, 0.2]	1,871,888	0.99640	0.7179	0.8891
			(0.2, 0.3]	1,487,374	0.99500	0.4168	0.7523
			(0.3, 0.4]	1,242,683	0.99409	0.1666	0.4580
			(0.4, 0.5]	1,126,168	0.99358	0.0278	0.2146

Lin's concordance correlation coefficient for CaseControl_AF, CaseControl_SE, and bias-corrected CaseControl_SE between true and estimated MAFs in real GWAS datasets.
[a]Prostate cancer MAFs were not corrected due to the small number of overlapping variants with gnomAD.

regression without covariates.[22] Using the MAF in CaseControl_SE also results in more variability, most likely due to a loss of information in allele polarity, which can result in errors when harmonizing datasets. To ensure the estimated AF is between zero and one, a key assumption of the SE method by Yang et al. is that the estimated allele is always the minor allele. As such, there is no connection between the reported alleles in the summary statistics and the estimated MAF. This loss of information regarding alleles can complicate secondary analyses, as accurately inferring the minor allele may not be possible, especially when the MAF is close to 0.5. Conversely, CaseControl_AF outputs the estimated case and control AFs for which the total sample AF was reported, retaining allele and AF pairing information.

Notably, both methods assume that the total AN is known. In reality, for a given variant, the total AN and the AN for cases and controls may vary across the variants. When available, a vector, rather than a single value, can be used for AN, providing variant-level information about the total AN. Unfortunately, variant-level AN is often not provided in summary statistics.

An interesting scenario for use beyond those explored here is meta-analysis. In a fixed-effects meta-analysis where the assumption is that all studies share the same underlying true effect, we expect the methods to work similarly to the scenarios evaluated here. However, in a meta-analysis using a random-effects or similar framework where the underlying true effects differ in the studies, it is possible that there would be additional variability in the AF estimates not captured by the current methods we present. Best practice is to publish both the meta-analysis summary statistics and the summary statistics for each study, in which case the methods presented here could be easily used.

As SE is more commonly available in GWAS summary data than total AF, we developed a bias correction that can be used when the total sample AF is not available. While bias correction requires harmonization of the observed data with a publicly available data source, such as gnomAD, we find that even a subset of the data, such as chromosome 1, as we use here, is sufficient to estimate and correct for bias. This eases computational and person-time burden and enables bias correction for variants not in the public proxy data. While the bias correction improves the accuracy of the case and control AF estimates, variability and bias in higher MAF bins remain and are especially prominent when the sample size is small. Additionally, a publicly available database containing ancestrally matched proxy AFs is required for the bias correction, highlighting the need for large, public, and ancestrally diverse databases. While gnomAD is an expansive resource for genomics, studies of admixed samples may not have an ancestrally matched gnomAD group to use as the proxy. For bias correction of the Pan-UKBB Diabetes GWAS in AFR individuals, we used gnomAD MAFs from the AFR/AFRAM group as proxies and found that, while not a perfect genetic-similarity

match, bias of the estimates was still greatly reduced. Summary data harmonization methods, such as *Summix2*,[23] can adjust AFs to match genetic similarity between samples and could be used to harmonize the population structure between the proxy and GWAS data here. The use of this bias correction framework for ancestrally diverse or admixed samples requires further investigation.

Conclusion

We have introduced methods and software to derive case and control AFs from GWAS summary data. These AFs can then be used in downstream analyses, such as in association studies as external controls, case-case GWAS, and meta-analysis. The functions available in the CCAFE package provide researchers with user-friendly and open-source methods to enhance the reuse of publicly available genetic summary statistics.

Data and code availability

The CCAFE software is available for download on GitHub (https://github.com/wolffha/CCAFE/) and through Bioconductor (https://bioconductor.org/packages/CCAFE/). The code used to generate simulated datasets and perform analyses is available at https://github.com/wolffha/CaseControlAF_manuscript.

Acknowledgments

This work was supported by the National Human Genome Research Institute (R35HG011293, R01HG011345, U01HG011715, and R01HL151152). This work also used the computing resources at the Center for Computational Mathematics, University of Colorado Denver, including the Alderaan cluster, supported by the National Science Foundation award OAC-2019089.

Declaration of interests

A.E.H. is an associate editor for *HGG Advances*. C.R.G. owns stock in 23andMe, Inc.

Supplemental information

Supplemental information can be found online at https://doi.org/10.1016/j.xhgg.2026.100616.

Web resources

Bioconductor - CCAFE, https://bioconductor.org/packages/CCAFE/

CRAN - PhenotypeSimulator, https://cran.r-project.org/web/packages/PhenotypeSimulator/index.html

EBI - Prostate Cancer GWAS, https://www.ebi.ac.uk/gwas/studies/GCST006085

GitHub - CCAFE, https://github.com/wolffha/CCAFE/

GitHub - CaseControlAF_manuscript, https://github.com/wolffha/CaseControlAF_manuscript

gnomAD, https://gnomad.broadinstitute.org/downloads

Pan-UK Biobank, https://pan-dev.ukbb.broadinstitute.org/docs/per-phenotype-files/index.html

Received: March 14, 2025
Accepted: April 16, 2026

References

1. Tam, V., Patel, N., Turcotte, M., Bossé, Y., Paré, G., and Meyre, D. (2019). Benefits and limitations of genome-wide association studies. Nat. Rev. Genet. *20*, 467–484. https://doi.org/10.1038/s41576-019-0127-1.
2. Kullo, I.J., Lewis, C.M., Inouye, M., Martin, A.R., Ripatti, S., and Chatterjee, N. (2022). Polygenic scores in biomedical research. Nat. Rev. Genet. *23*, 524–532. https://doi.org/10.1038/s41576-022-00470-z.
3. van Rheenen, W., Peyrot, W.J., Schork, A.J., Lee, S.H., and Wray, N.R. (2019). Genetic correlations of polygenic disease traits: from theory to practice. Nat. Rev. Genet. *20*, 567–581. https://doi.org/10.1038/s41576-019-0137-z.
4. Sanderson, E., Glymour, M.M., Holmes, M.V., Kang, H., Morrison, J., Munafò, M.R., Palmer, T., Schooling, C.M., Wallace, C., Zhao, Q., and Smith, G.D. (2022). Mendelian randomization. Nat. Rev. Methods Primers *2*, 6. https://doi.org/10.1038/s43586-021-00092-5.
5. Lee, S., Kim, S., and Fuchsberger, C. (2017). Improving power for rare-variant tests by integrating external controls. Genet. Epidemiol. *41*, 610–619. https://doi.org/10.1002/gepi.22057.
6. Hendricks, A.E., Billups, S.C., Pike, H.N.C., Farooqi, I.S., Zeggini, E., Santorico, S.A., Barroso, I., and Dupuis, J. (2018). ProxECAT: Proxy External Controls Association Test. A new case-control gene region association test using allele frequencies from public controls. PLoS Genet. *14*, e1007591. https://doi.org/10.1371/journal.pgen.1007591.
7. Wojcik, G.L., Murphy, J., Edelson, J.L., Gignoux, C.R., Ioannidis, A.G., Manning, A., Rivas, M.A., Buyske, S., and Hendricks, A.E. (2022). Opportunities and challenges for the use of common controls in sequencing studies. Nat. Rev. Genet. *23*, 665–679. https://doi.org/10.1038/s41576-022-00487-4.
8. Lyon, M.S., Andrews, S.J., Elsworth, B., Gaunt, T.R., Hemani, G., and Marcora, E. (2021). The variant call format provides efficient and robust storage of GWAS summary statistics. Genome Biol. *22*, 32. https://doi.org/10.1186/s13059-020-02248-0.
9. Matushyn, M., Bose, M., Mahmoud, A.A., Cuthbertson, L., Tello, C., Bircan, K.O., Terpolovsky, A., Bamunusinghe, V., Khan, U., Novković, B., et al. (2022). SumStatsRehab: an efficient algorithm for GWAS summary statistics assessment and restoration. BMC Bioinf. *23*, 443. https://doi.org/10.1186/s12859-022-04920-7.
10. Thelwall, M., Munafò, M., Mas-Bleda, A., Stuart, E., Makita, M., Weigert, V., Keene, C., Khan, N., Drax, K., and Kousha, K. (2020). Is useful research data usually shared? An investigation of genome-wide association study summary statistics. PLoS One *15*, e0229578. https://doi.org/10.1371/journal.pone.0229578.
11. Murphy, A.E., Schilder, B.M., and Skene, N.G. (2021). MungeSumstats: a Bioconductor package for the standardization and quality control of many GWAS summary statistics. Bioinformatics *37*, 4593–4596. https://doi.org/10.1093/bioinformatics/btab665.
12. Buniello, A., MacArthur, J.A.L., Cerezo, M., Harris, L.W., Hayhurst, J., Malangone, C., McMahon, A., Morales, J., Mountjoy, E., Sollis, E., et al. (2019). The NHGRI-EBI GWAS Catalog of published genome-wide association studies, targeted

arrays and summary statistics 2019. Nucleic Acids Res. *47*, D1005–D1012. https://doi.org/10.1093/nar/gky1120.

13. Hayhurst, J., Buniello, A., Harris, L., Mosaku, A., Chang, C., Gignoux, C.R., Hatzikotoulas, K., Karim, M.A., Lambert, S.A., Lyon, M., et al. (2023). A Community Driven GWAS Summary Statistics Standard. Preprint at bioRxiv. https://doi.org/10.1101/2022.07.15.500230.
14. Yang, Z., Paschou, P., and Drineas, P. (2022). Reconstructing SNP allele and genotype frequencies from GWAS summary statistics. Sci. Rep. *12*, 8242. https://doi.org/10.1038/s41598-022-12185-6.
15. R Core Team. (2024). R: A Language and Environment for Statistical Computing (R Foundation for Statistical Computing).
16. Huber, W., Carey, V.J., Gentleman, R., Anders, S., Carlson, M., Carvalho, B.S., Bravo, H.C., Davis, S., Gatto, L., Girke, T., et al. (2015). Orchestrating high-throughput genomic analysis with Bioconductor. Nat. Methods *12*, 115–121. https://doi.org/10.1038/nmeth.3252.
17. Meyer, H.V., and Birney, E. (2018). PhenotypeSimulator: A comprehensive framework for simulating multi-trait, multi-locus genotype to phenotype relationships. Bioinformatics *34*, 2951–2956. https://doi.org/10.1093/bioinformatics/bty197.
18. Lin, L.I. (1989). A concordance correlation coefficient to evaluate reproducibility. Biometrics *45*, 255–268.
19. Schumacher, F.R., Al Olama, A.A., Berndt, S.I., Benlloch, S., Ahmed, M., Saunders, E.J., Dadaev, T., Leongamornlert, D., Anokian, E., Cieza-Borrella, C., et al. (2018). Association analyses of more than 140,000 men identify 63 new prostate cancer susceptibility loci. Nat. Genet. *50*, 928–936. https://doi.org/10.1038/s41588-018-0142-8.
20. Karczewski, Gupta, Kanai, et al. (2025). Pan-UK Biobank genome-wide association analyses enhance discovery and resolution of ancestry-enriched effects. Nat Genet *57*, 2408–2417. https://doi.org/10.1038/s41588-025-02335-7.
21. Karczewski, K.J., Francioli, L.C., Tiao, G., Cummings, B.B., Alföldi, J., Wang, Q., Collins, R.L., Laricchia, K.M., Ganna, A., Birnbaum, D.P., et al. (2020). The mutational constraint spectrum quantified from variation in 141,456 humans. Nature *581*, 434–443. https://doi.org/10.1038/s41586-020-2308-7.
22. Xing, G., and Xing, C. (2010). Adjusting for covariates in logistic regression models. Genet. Epidemiol. *34*, 769–772. https://doi.org/10.1002/gepi.20526.
23. Stoneman, H.R., Price, A., Trout, N.S., Lamont, R., Tifour, S., Pozdeyev, N., Colorado Center for Personalized Medicine, Crooks, K., Lin, M., Rafaels, N., et al. (2025). Characterizing substructure via mixture modeling in large-scale genetic summary statistics. American Journal of Human Genetics. https://doi.org/10.1016/j.ajhg.2024.12.007.

HGGA, Volume 7

Supplemental information

CCAFE: Estimating case and control allele frequencies from GWAS summary statistics

Hayley R. Stoneman, Hugo Lemus Gomez, Adelle Price, Christopher R. Gignoux, and Audrey E. Hendricks

Note S1. Full Derivation for CaseControl_AF

Known:

$$AF_{total} = \frac{(N_{case}AF_{case} + N_{control}AF_{control})}{N_{total}} \tag{1}$$

$$OR = \frac{ad}{bc} \tag{2}$$

Where:

$a = 2N_{case} * AF_{case}$
$b = 2N_{case}(1 - AF_{case})$
$c = 2N_{control} * AF_{control}$
$d = 2N_{control}(1 - AF_{control})$

Find $AF_{control}$ and AF_{case}:

Substitute for $a, b, c, \& \ d$ into equation (2) and simplify:

$$OR = \frac{(2N_{case} * AF_{case})[2N_{control}(1 - AF_{control})]}{[2N_{case}(1 - AF_{case})](2N_{control} * AF_{control})} \tag{3}$$

$$OR = \frac{AF_{case}(1 - AF_{control})}{(1 - AF_{case})AF_{control}} \tag{4}$$

Substitute for AF_{case} using equation (1) and distribute:

$$OR = \frac{(\frac{N_{total}}{N_{case}}AF_{total} - \frac{N_{control}}{N_{case}}AF_{control})(1 - AF_{control})}{[1 - \frac{N_{total}}{N_{case}}AF_{total} + \frac{N_{control}}{N_{case}}AF_{control}]AF_{control}} \tag{5}$$

$$OR = \frac{\frac{N_{total}}{N_{case}}AF_{total} - AF_{control}\frac{N_{control}}{N_{case}} - AF_{control}\left(\frac{N_{total}}{N_{case}}AF_{pop}\right) + AF_{control}^2(\frac{N_{control}}{N_{case}})}{AF_{control}[1 - \left(\frac{N_{total}}{N_{case}}AF_{total}\right)] + AF_{control}^2(\frac{N_{control}}{N_{case}})} \tag{6}$$

$$\begin{aligned} AF_{control}^2\left(\frac{N_{control}}{N_{case}}\right)OR + AF_{control}\left[1 - \left(\frac{N_{total}}{N_{case}}AF_{total}\right)\right]OR \\ = AF_{control}^2\left(\frac{N_{control}}{N_{case}}\right) - AF_{control}\left(\frac{N_{control}}{N_{case}} + \frac{N_{total}}{N_{case}}AF_{total}\right) + \frac{N_{total}}{N_{case}}AF_{total} \end{aligned} \tag{7}$$

Arrange as quadratic equation:

$$AF_{control}^2\left[\frac{N_{control}}{N_{case}}(OR - 1)\right] + AF_{control}\left[OR\left(1 - \left(\frac{N_{total}}{N_{case}}AF_{total}\right)\right) + \frac{1}{N_{case}}(N_{control} + N_{total}AF_{total})\right] - \frac{N_{total}}{N_{case}}AF_{total} = 0 \tag{8}$$

Let:

$$\left[a = \frac{N_{control}}{N_{case}}(OR - 1)\right] \tag{9}$$

$$b = \left[OR\left(1 - \left(\frac{N_{total}}{N_{case}}AF_{total}\right)\right) + \frac{1}{N_{case}}(N_{control} + N_{total}AF_{total})\right] \quad (10)$$

$$c = -\frac{N_{total}}{N_{case}}AF_{total} \quad (11)$$

$$AF_{control}^2 a + AF_{control} b + c = 0 \quad (12)$$

Choose the root greater than 0 and less than 1 to be $AF_{control}$ where x_1, x_2 are the roots

$$x_{1,2} = \frac{-b \pm \sqrt{b^2 - 4ac}}{2a} \quad (13)$$

$$AF_{control} = \begin{cases} x_1, & if\ 0 \le x_1 \le 1 \\ x_2, & otherwise \end{cases}$$

Use the calculated $AF_{control}$ to solve for AF_{case} where:

$$AF_{case} = \frac{N_{total}}{N_{case}}AF_{total} - \frac{N_{control}}{N_{case}}AF_{control} \quad (14)$$

Note S2. Simulations Show There is Only One Root [0,1]

Given the quadratic in equation (8) and the coefficients in equations (9 – 11), we sought to determine the possible solutions for the roots as shown in equation (13). Keeping N_{total} constant at 10,000, we examined 144 different scenarios with varying AF, OR, N_{case}, and $N_{control}$ as shown in Table S1 below (all combinations and results in Table S2).

We used the values of the parameters to compute *a, b, c* as shown in equations (9-11), followed by calculation of the two roots using equation (13). Root 1 (x_1) and root 2 (x_2) were calculated as follows:

$$x_1 = \frac{-b - \sqrt{b^2 - 4ac}}{2a} \quad (15)$$

$$x_2 = \frac{-b + \sqrt{b^2 - 4ac}}{2a} \quad (16)$$

We observed the results shown in Figure S1, indicating that for possible combinations of parameters, only one root (x_2) lies within [0,1]. Also note that when $a > 0$, $x_1 < 0$, and when $a < 0$, $x_1 > 1$.

Note S3. Proof That Only 1 Root Lies Within [0,1]

We first note that there are two conditions for which we may need to assess the bounds of the roots, $a > 0\ and\ a < 0$. Looking at equation (9), we see that a is positive when $OR > 1$ and a is negative when $OR < 1$. Since we are working with biallelic variants, we are able to simply switch the allele ($1 - AF$) which will switch the OR to either greater than or less than 1. Thus, we only need to solve the proof under one condition; specifically, we will show that only 1 root lies within [0,1] for the case in which $a > 0$.

We start with the following inequality

$$a + b + c \geq 0 \tag{17}$$

We can show that this is true by plugging in our values for a, b, c from equations (9-11). First, we rearrange each equation to have the same denominator.

Rearrange:

$$a = \frac{OR * N_{control} - N_{control}}{N_{case}} \tag{18}$$

Rearrange:

$$b = OR\left(\frac{N_{case} - N_{total}AF_{total}}{N_{case}}\right) + \frac{N_{control} + N_{total}AF_{total}}{N_{case}} \tag{19}$$

$$b = \frac{OR * N_{case} - OR * N_{total}AF_{total} + N_{control} + N_{total}AF_{total}}{N_{case}} \tag{20}$$

Rearrange:

$$c = -\frac{N_{total}AF_{total}}{N_{case}} \tag{21}$$

Now plug in equations (18, 20, 21) into (17).

$$\frac{OR * N_{control} - N_{control} + OR * N_{case} - OR * N_{total}AF_{total} + N_{control} + N_{total}AF_{total} - N_{total}AF_{total}}{N_{case}} \geq 0 \tag{22}$$

$$\frac{OR(N_{case} + N_{control}) - OR * N_{total}AF_{total}}{N_{case}} \geq 0 \tag{23}$$

$$\frac{OR * N_{total} - OR * N_{total}AF_{total}}{N_{case}} \geq 0 \tag{24}$$

$$\frac{OR * N_{total}(1 - AF_{total})}{N_{case}} \geq 0 \tag{25}$$

Since we know $0 \leq AF_{total} \leq 1, OR > 0, N_{total} > 0, N_{case} > 0$ we know that the inequality in (25) is true, thus proving that under these conditions, the inequality in (17) is true.

We can now use equation (17) to prove that $x_2 < 1$ (as a reminder, x_2 is defined in equation (16)).

$$a + b + c \geq 0 \tag{17}$$

Rearrange

$$a + b \geq -c \tag{26}$$

Multiply by $4a$ (reminder $a > 0$)

$$4a^2 + 4ab \geq -4ac \tag{27}$$

Add b^2 to both sides

$$4a^2 + 4ab + b^2 \geq b^2 - 4ac \tag{28}$$

Factor the right side

$$(2a + b)^2 \geq b^2 - 4ac \tag{29}$$

Take the square root of both sides

$$2a + b \geq \sqrt{b^2 - 4ac} \tag{30}$$

Subtract b and divide by $2a$

$$1 \geq \frac{-b + \sqrt{b^2 - 4ac}}{2a} \tag{31}$$

Substitute equation (16) in the right side

$$1 \geq x_2 \tag{32}$$

We now use the following inequality to prove that $x_2 \geq 0$ as well. We start with the following:

$$-4ac \geq 0 \tag{33}$$

We know that the equality shown in (33) is true because $c < 0$ and we assumed that we are under the condition in which $a > 0$, making the left side of the equality a positive number (or 0).

Add b^2 to both sides

$$b^2 - 4ac \geq b^2 \tag{34}$$

Take the square root of both sides

$$\sqrt{b^2 - 4ac} \geq b \tag{35}$$

Subtract b and divide by $2a$

$$\frac{-b + \sqrt{b^2 - 4ac}}{2a} \geq 0 \tag{36}$$

Substitute equation (16) in the left side

$$x_2 \geq 0 \tag{37}$$

Finally, we show that the other root, x_1 is outside of the valid range of solutions for $AF_{control}$

As a reminder:

$$-4ac \geq 0 \tag{33}$$

Add b^2 to both sides

$$b^2 - 4ac \geq b^2 \tag{38}$$

Take the square root of both sides, this time keeping the negative b

$$\sqrt{b^2 - 4ac} \geq -b \tag{39}$$

Subtract square root term and divide by $2a$

$$0 \geq \frac{-b - \sqrt{b^2 - 4ac}}{2a} \tag{40}$$

Substitute equation (15) in the right side

$$0 \geq x_1 \tag{41}$$

We have now proved that under the assumption that $a > 0$, $0 \leq x_2 \leq 1$, and $x_1 \leq 0$, thus proving that there is only one valid solution for $AF_{control}$.

Note S4. Recommended Number of Variants Per MAF Bin for SE Bias Correction

In order to determine the recommended number of overlapping variants between the proxy data and the user dataset we assessed the impact on the bias correction for the Pan-UKBB diabetes data using

gnomAD as a proxy for both African and European samples. We again used the 1,212,618 chromosome one variants that were harmonized with Pan-UKBB (see **2.2.2**). We subset the number of variants (100, 500, 1000, 5000, 10000, 15000, 30000, 50000) per bin ([0, 0.1), [0.1, 0.2), [0.2, 0.3), [0.3, 0.4), [0.4, 0.5]) over 10 replicates and plotted the mean and median bias per MAF bin. We compared the bias to the unadjusted estimates for all >9 million genome-wide variants in each sample.

In both Pan-UKBB AFR and EUR samples, we observe minimal difference in the adjusted MAF estimates using any number of variants per bin equal to or greater than 10,000 (**Figure S2-S3**). Therefore, we recommend for best results of bias correction a minimum number of variants per bin of 10,000.

Note S5. Replication of embedded code from ReACt

The function to derive case and control AFs from the SE is embedded within a publicly available meta-analysis software ReACt written in C (https://github.com/Paschou-Lab/ReACt). To provide a standalone function for AF estimation, we translated it into R as the CCAFE software function CaseControl_SE. To ensure that the translation was accurate, we tested whether we could replicate the case-case GWAS (CC-GWAS) results published in Yang et al in R that examined bipolar disorder and schizophrenia GWAS results from the Psychiatric Genomics Consortium in CC-GWAS. When we tested our R implementation of their software using the same data and we were able to replicate the published results.

Note S6. Case AF Derivation for Prostate Cancer GWAS

We selected a prostate cancer PGS based on a GWAS that published OR and control AF for each variant (Schumacher et al., 2018). Using the case and control sample sizes ($N_{case}, N_{control}$) we derived allele counts (AC) for the effect and non-effect alleles using $AC = AF * N$. The OR can be calculated using ACs as follows:

$$OR = \frac{AC_{effect,case} * AC_{non-effect,control}}{AC_{effect,control} * AC_{non-effect,case}} \quad (42)$$

We rearrange this equation to solve for the effect allele AC in cases, as needed for our reference data, as follows:

$$AC_{effect,case} = \frac{OR * AC_{effect,control}}{1 - AC_{effect,control} + OR * AC_{effect,control}} \quad (43)$$

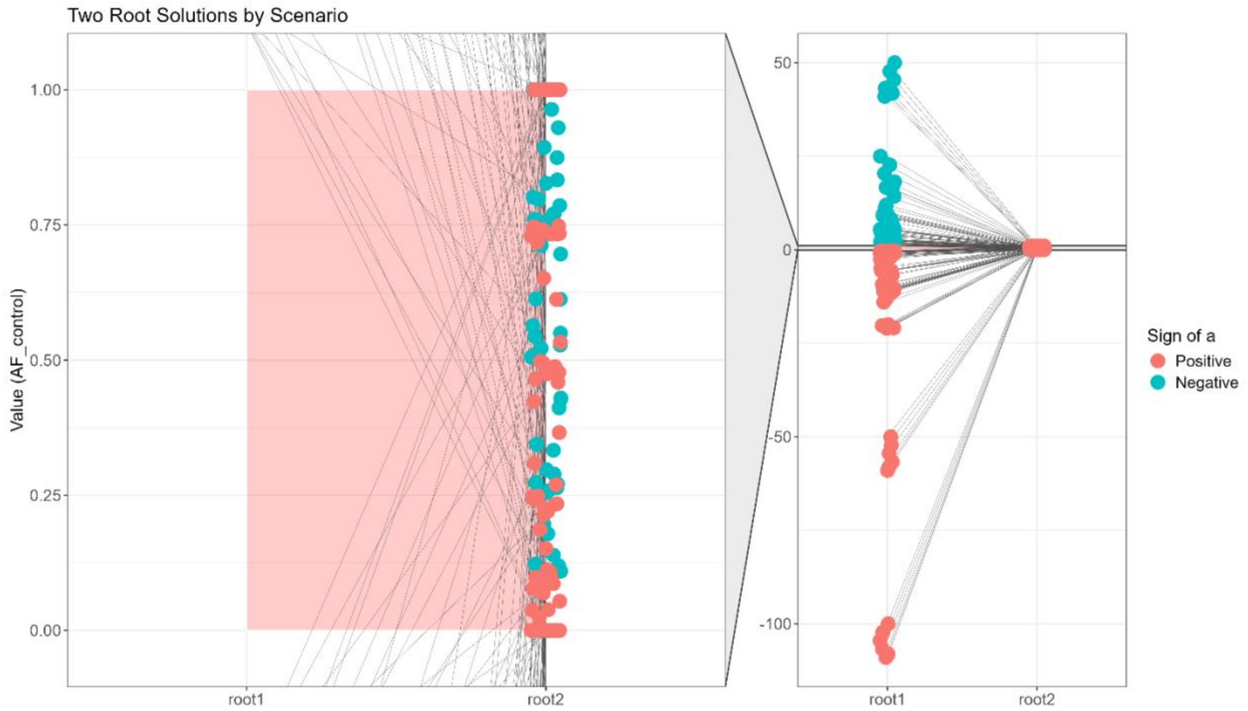

Figure S1. Simulations show only one root solves for valid control AF value. Simulations of 144 different scenarios with varying case and control sample size, OR, and AF were used to calculate the roots (two possible solutions for control AF). Root 2, calculated by equation (16) is always in the valid interval [0,1] (shown in the red rectangle), while root 1, calculated by equation (15) is always outside of the valid interval. We also note that when the coefficient *a* is positive, root 1 is lower than the valid interval (i.e. < 0) and when *a* is negative, root 1 is greater than the valid interval (i.e. > 1).

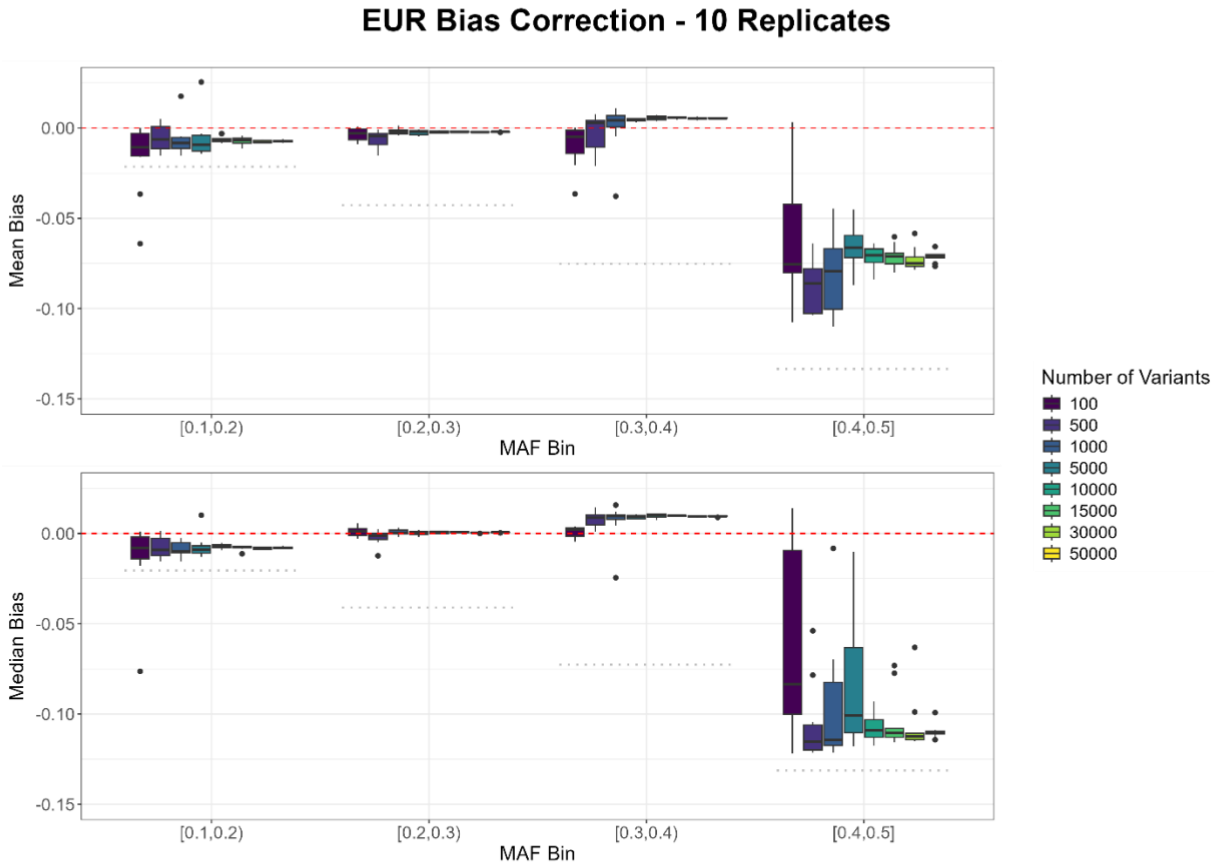

Figure S2. Number of variants needed for bias correction framework to adjust CaseControl_SE estimates using gnomAD as proxies in EUR. The gnomAD v3.1.2 chromosome 1 data (1,212,618 total variants) for the non-Finish European group (NFE) was subset for a given number of variants (shown in the key on the right) per MAF bin (x-axis) for 10 replicates. This subset of variants was used to correct the bias for all >9 million genome-wide variants in the Pan-UKBB EUR diabetes dataset. The mean (top) and median (bottom) bias was calculated per bin. The unadjusted bias is shown with the dotted grey line, while zero bias is shown with the dotted red line.

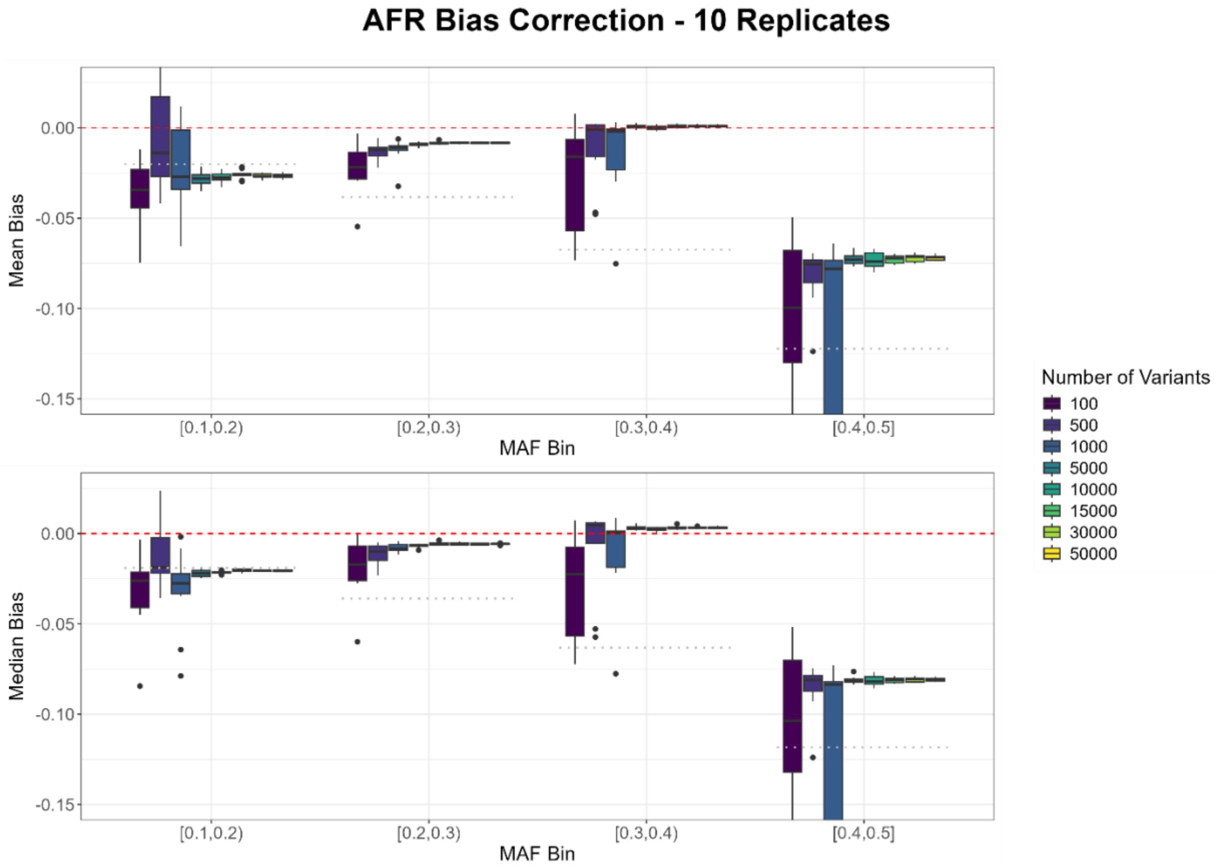

Figure S3. Number of variants needed for bias correction framework to adjust CaseControl_SE estimates using gnomAD as proxies in AFR. The gnomAD v3.1.2 chromosome 1 data (1,212,618 total variants) for the African/African American group (AFR/AFRAM) was subset for a given number of variants (shown in the key on the right) per MAF bin (x-axis) for 10 replicates. This subset of variants was used to correct the bias for all >9 million genome-wide variants in the Pan-UKBB AFR diabetes dataset. The mean (top) and median (bottom) bias was calculated per bin. The unadjusted bias is shown with the dotted grey line, while zero bias is shown with the dotted red line.

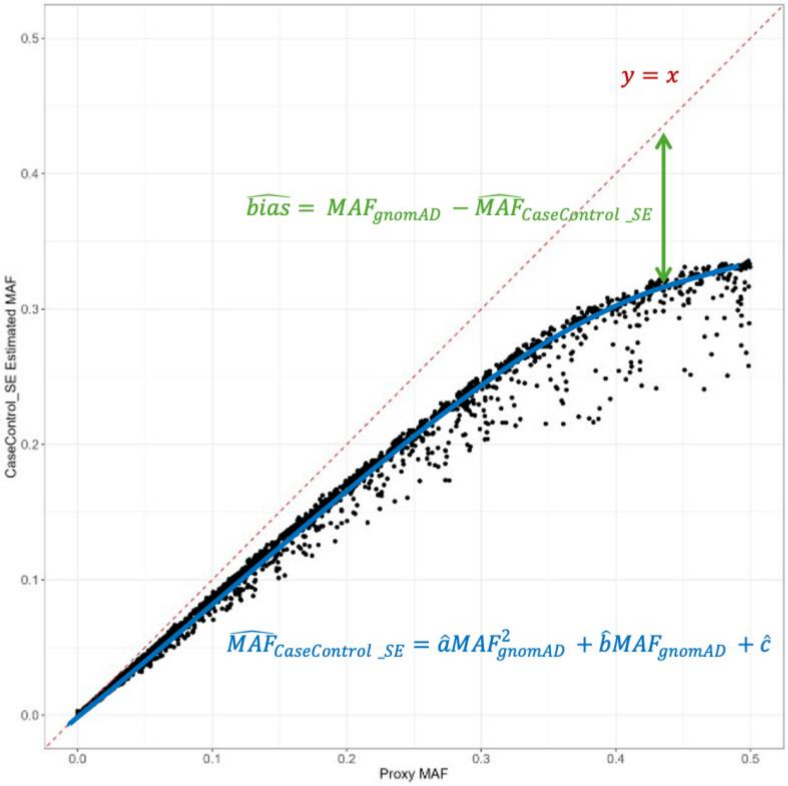

Figure S4. Bias correction framework to adjust CaseControl_SE estimates using gnomAD as proxies. The relationship between the proxy MAF (x-axis) and the estimated MAF (y-axis) is modeled through polynomial regressions (blue). This model is used to estimate the bias (green). The adjusted MAF estimate is estimated by adding the bias to the CaseControl_SE MAF output.

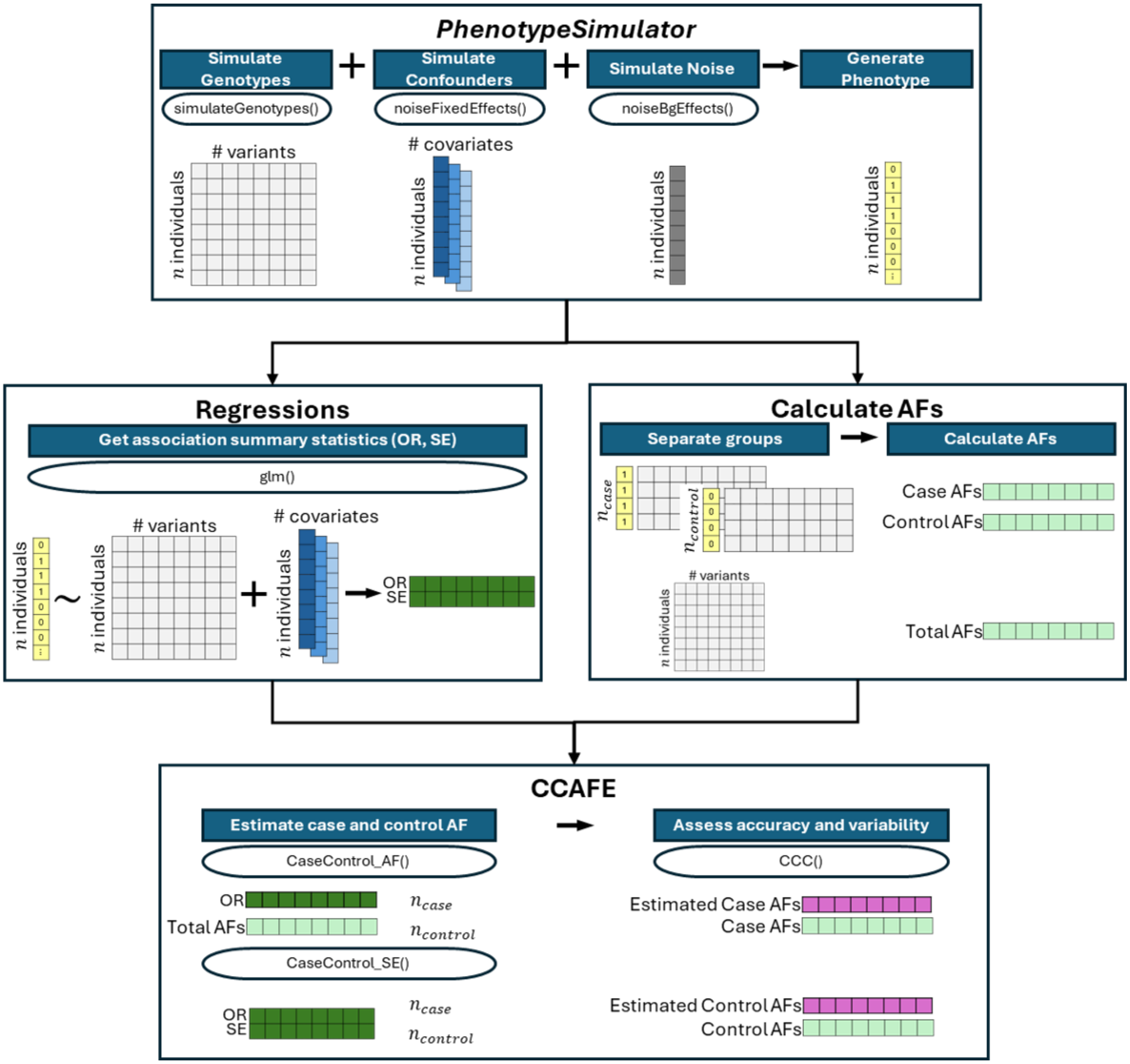

Figure S5. Simulation framework to assess *CCAFE* accuracy and variability. The *PhenotypeSimulator* R package was used to simulate genotypes, a binary phenotype, and covariates. Logistic regression was fit using the simulated genotypes and phenotypes to obtain association summary statistics (OR, SE). The phenotype (i.e., case/control status) was also used to calculate AF for cases and controls. These simulations were used to evaluate CaseControl_AF and CaseControl_SE using the OR, case and control sample sizes, and either total AF or SE respectively The output estimated case and control AFs were compared to the simulated case and control AFs estimating bias, variance, and agreement using Lin's CCC.

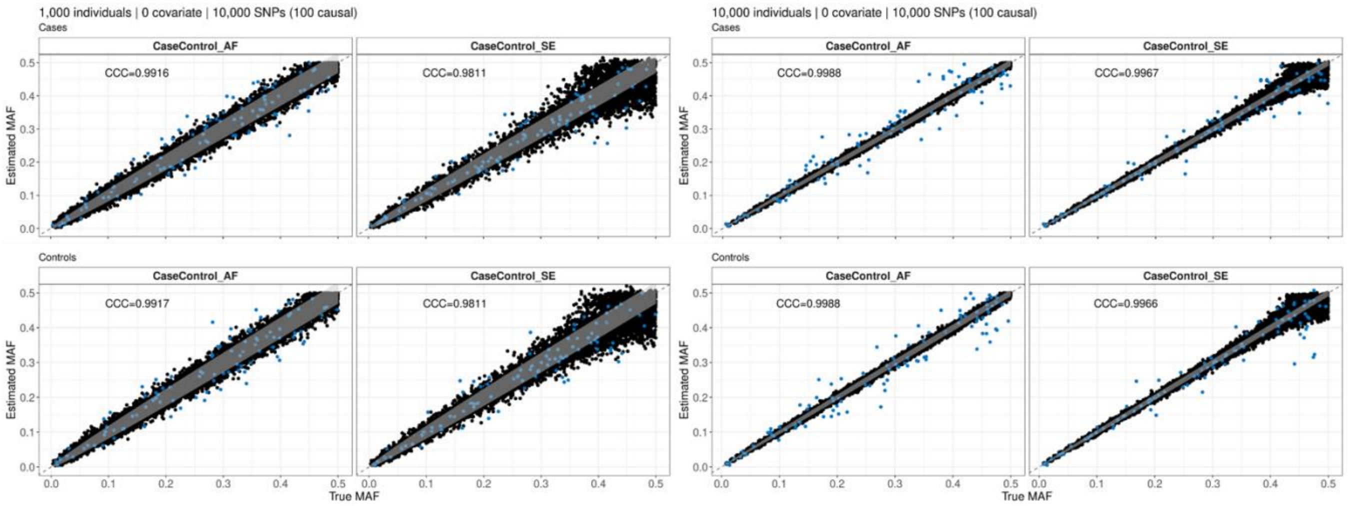

Figure S6. Given no covariates in the original GWAS, CaseControl_AF produces estimates within expected variance while CaseControl_SE has increased variance at high MAFs. Simulated genotypes for 10,000 SNPs of which 100 were causal (blue) and binary (case/control) phenotypes were generated using the PhenotypeSimulator R package with no covariates for N=1,000 (500 cases and 500 controls, **left panel**) and N=10,000 (5,000 cases and 5,000 controls, **right panel**). Logistic regression was used to generate summary statistics. The CCAFE R package was applied to reconstruct the case and control AFs with total AF (1st and 3rd columns) or SE (2nd and 4th columns). The 95% CI of a proportion (AF) given the sample size (N=1000 or 10,000) is shown in the grey ribbon. Using the SE, variance of the estimates was increased for higher MAFs beyond that expected from the CI while variance of the estimates from total AF was similar o the CI. CCC values are reported in Table 1.

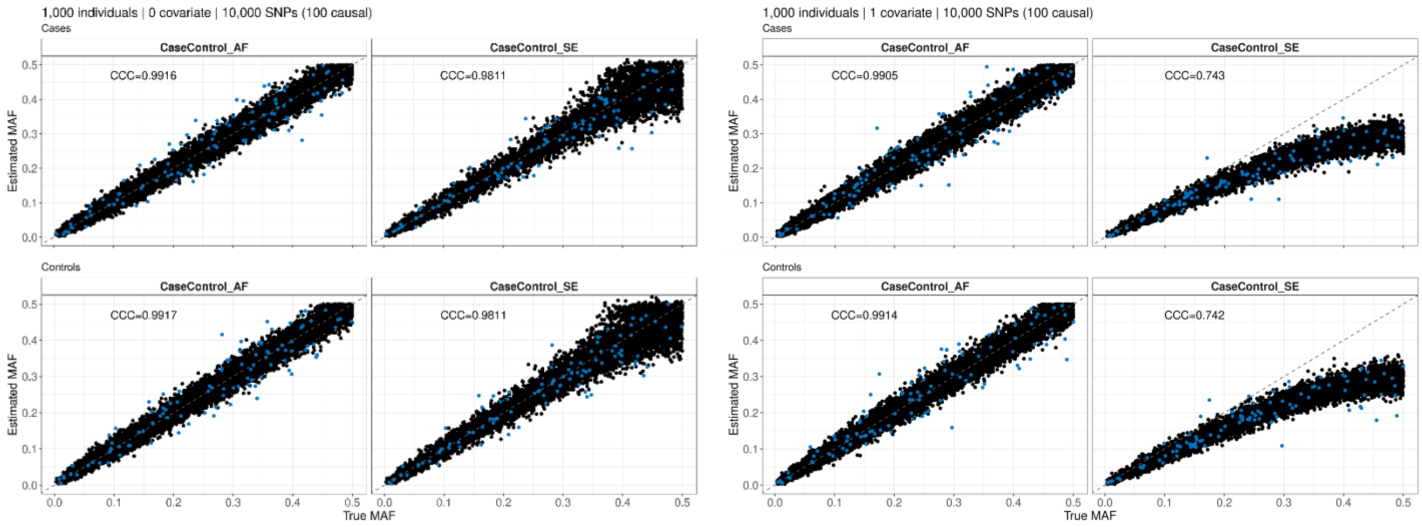

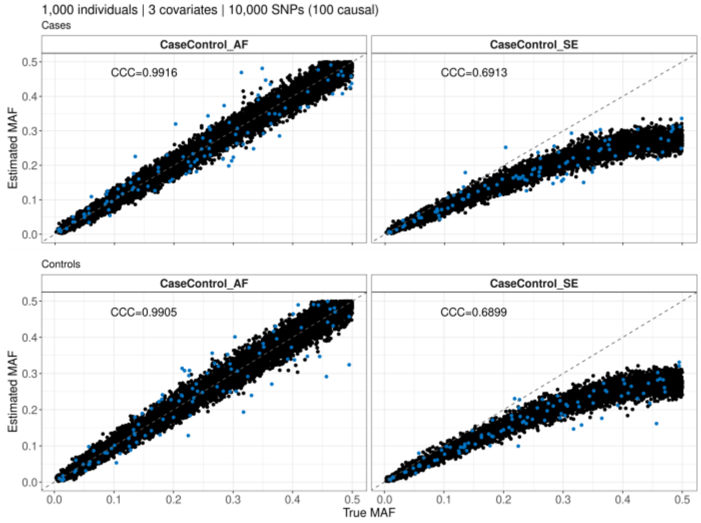

Figure S7. For small sample sizes (N=1,000), CaseControl_AF produces unbiased AF estimates while CaseControl_SE produces biased results in simulation when covariates included. Simulated genotypes and binary (case/control) phenotypes were generated using the PhenotypeSimulator R package for N=1,000 (500 cases and 500 controls). Genotypes for 10,000 SNPs, of which 100 were causal (blue), were generated for 1000 individuals. Logistic regression was used along with 0 (top left), 1 (top right), or 3 (bottom left) simulated covariates to generate summary statistics. The CCAFE R package was used to reconstruct the case and control AFs with total AF (left plots within each quadrant) or SE (right plots within each quadrant). Using the SE, bias existed and was greater for higher MAFs and when more covariates were included. Using total AF was accurate across the simulation parameters evaluated. CCC values are reported in Table 1.

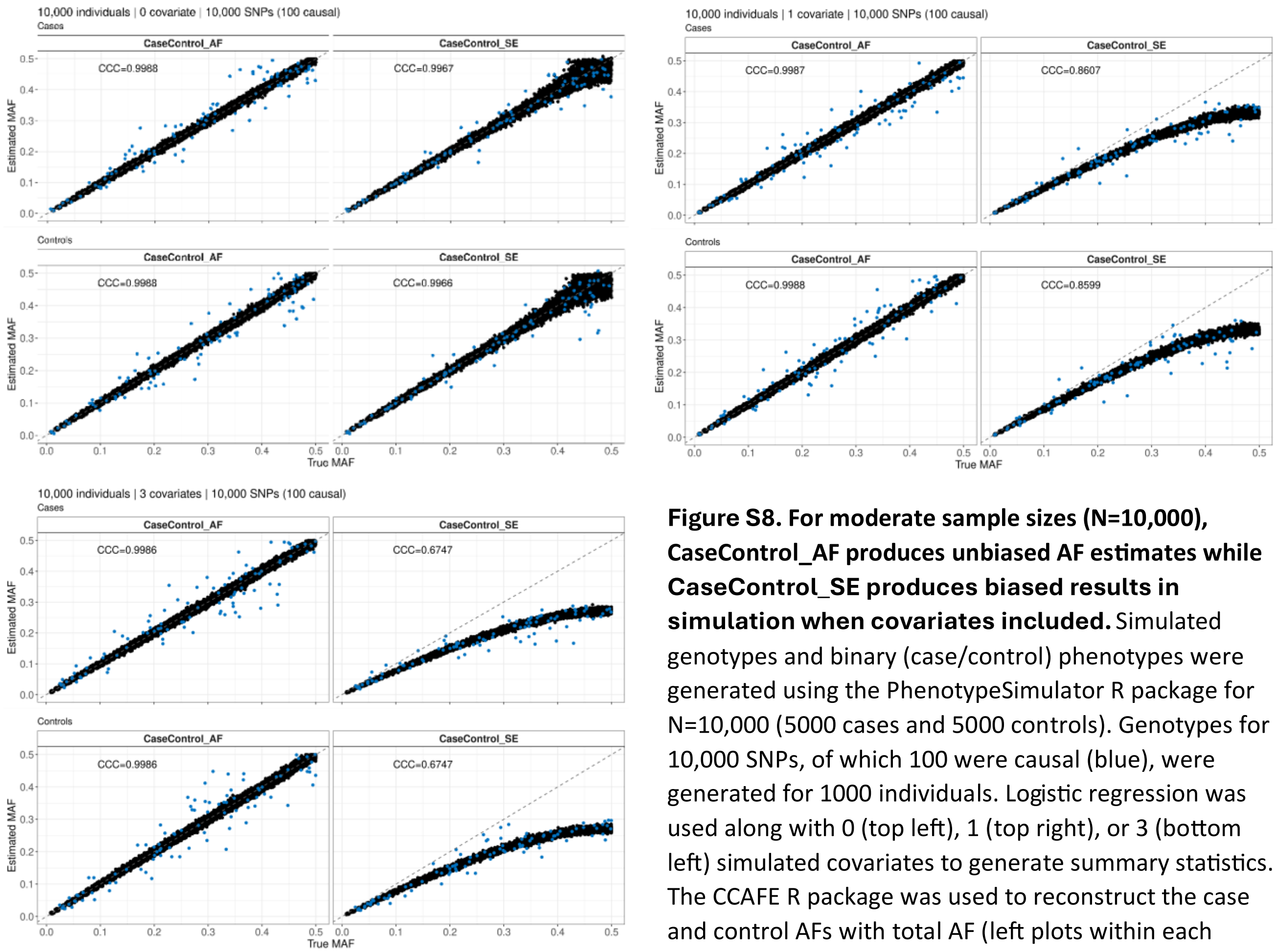

Figure S8. For moderate sample sizes (N=10,000), CaseControl_AF produces unbiased AF estimates while CaseControl_SE produces biased results in simulation when covariates included. Simulated genotypes and binary (case/control) phenotypes were generated using the PhenotypeSimulator R package for N=10,000 (5000 cases and 5000 controls). Genotypes for 10,000 SNPs, of which 100 were causal (blue), were generated for 1000 individuals. Logistic regression was used along with 0 (top left), 1 (top right), or 3 (bottom left) simulated covariates to generate summary statistics. The CCAFE R package was used to reconstruct the case and control AFs with total AF (left plots within each quadrant) or SE (right plots within each quadrant). Using the SE, bias existed and was greater for higher MAFs and when more covariates were included. Using total AF was accurate across the simulation parameters evaluated. CCC values are reported in Table 1.

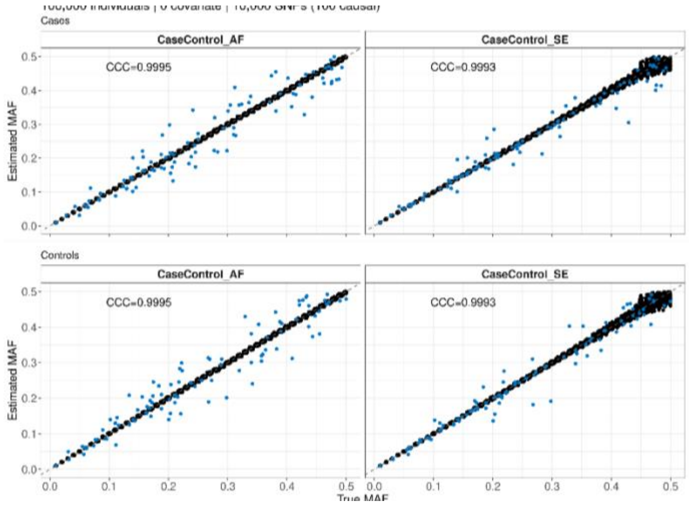

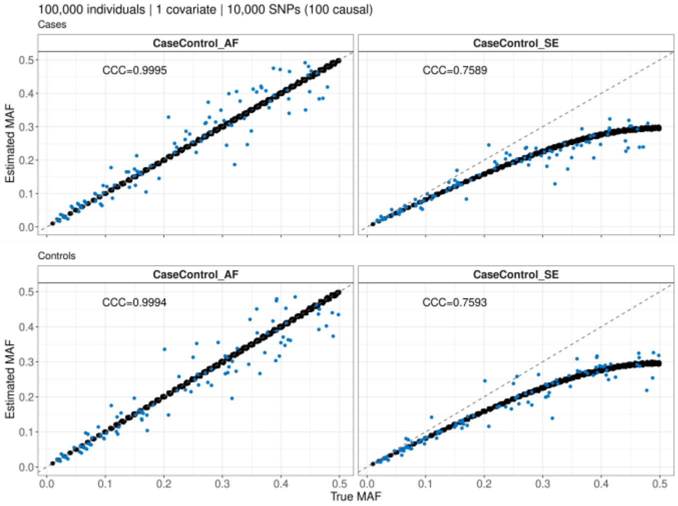

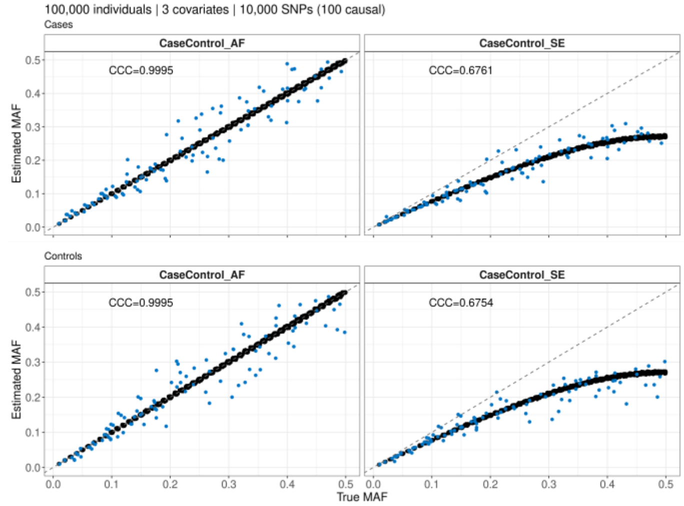

Figure S9. For large sample sizes (N=100,000), CaseControl_AF produces unbiased AF estimates while CaseControl_SE produces biased results in simulation when covariates included. Simulated genotypes and binary (case/control) phenotypes were generated using the PhenotypeSimulator R package for N=100,000 (50,000 cases and 50,000 controls). Genotypes for 10,000 SNPs, of which 100 were causal (blue), were generated for 1000 individuals. Logistic regression was used along with 0 (top left), 1 (top right), or 3 (bottom left) simulated covariates to generate summary statistics. The CCAFE R package was used to reconstruct the case and control AFs with total AF (left plots within each quadrant) or SE (right plots within each quadrant). Using the SE, bias existed and was greater for higher MAFs and when more covariates were included. Using total AF was accurate across the simulation parameters evaluated. CCC values are reported in Table 1.

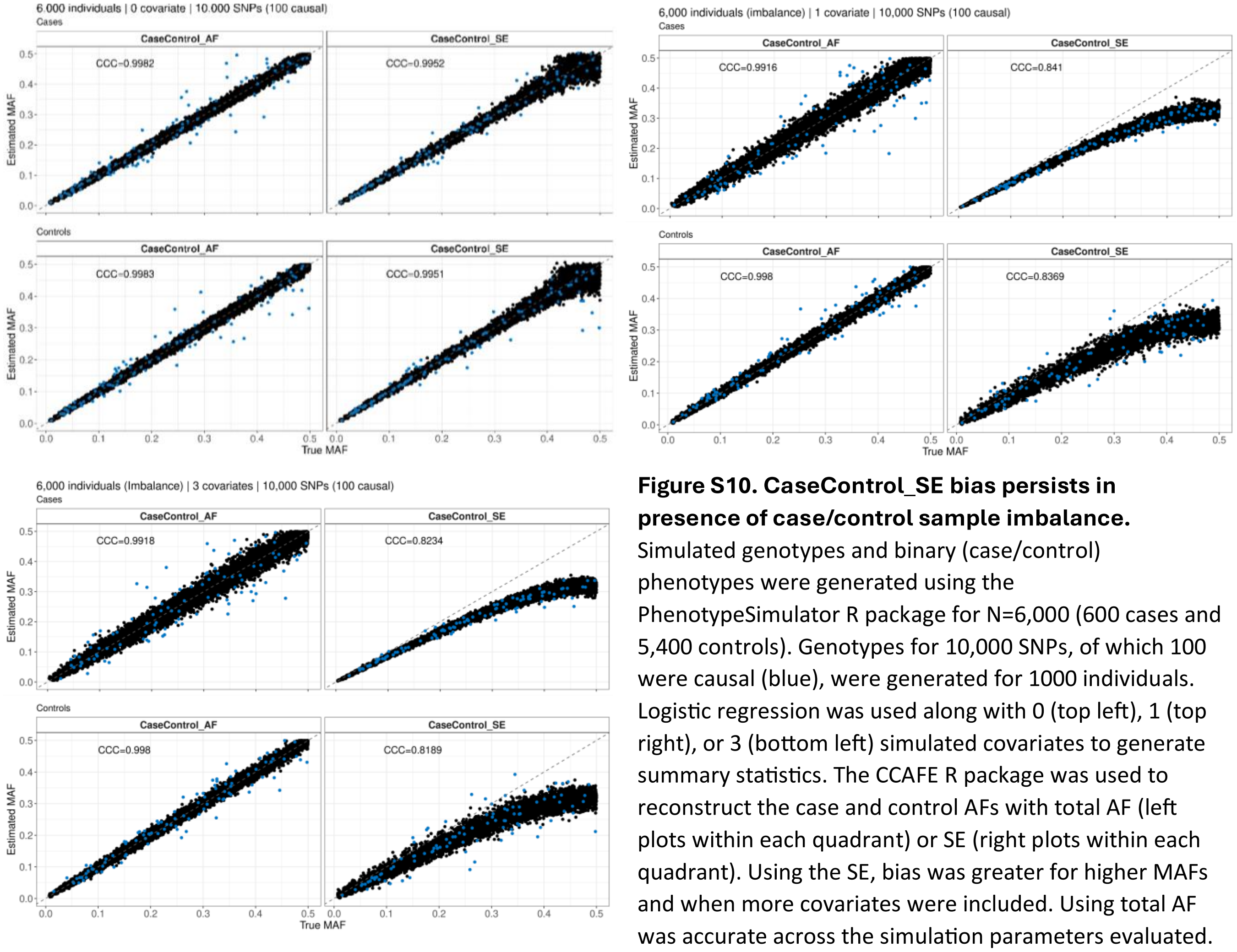

Figure S10. CaseControl_SE bias persists in presence of case/control sample imbalance. Simulated genotypes and binary (case/control) phenotypes were generated using the PhenotypeSimulator R package for N=6,000 (600 cases and 5,400 controls). Genotypes for 10,000 SNPs, of which 100 were causal (blue), were generated for 1000 individuals. Logistic regression was used along with 0 (top left), 1 (top right), or 3 (bottom left) simulated covariates to generate summary statistics. The CCAFE R package was used to reconstruct the case and control AFs with total AF (left plots within each quadrant) or SE (right plots within each quadrant). Using the SE, bias was greater for higher MAFs and when more covariates were included. Using total AF was accurate across the simulation parameters evaluated. CCC values are reported in Table 1.

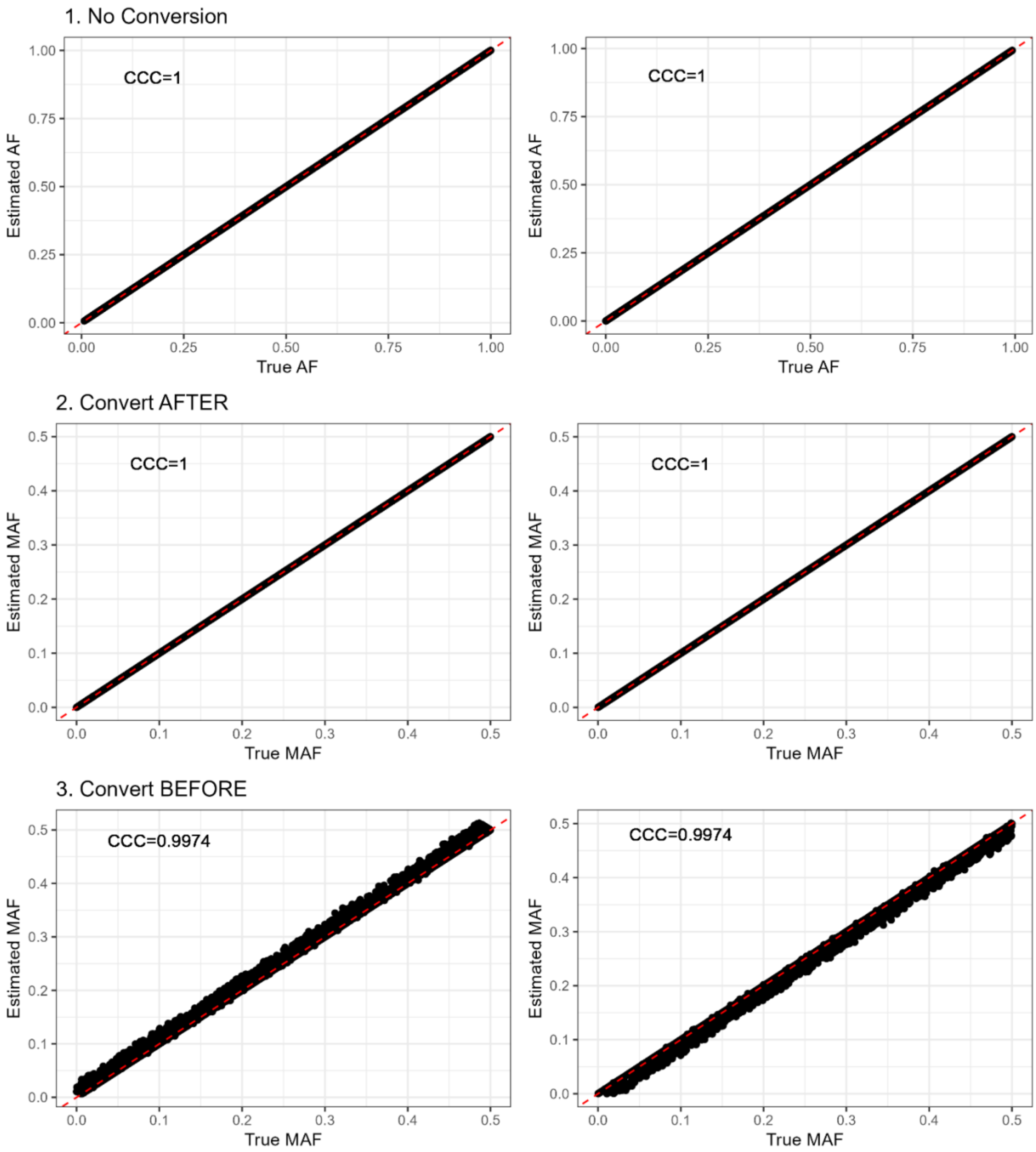

Figure S11. Converting AF to MAF prior to case and control AF estimation increases variability in case and control estimates using CaseControl_AF. Using 2400 simulated variants, three scenarios were used to estimate case AF (Left) and control AF (Right) with CaseControl_AF: 1) using the total AF to estimate case and control AFs 2) using total AF to estimate case and control AFs then converting to minor AF (MAF) 3) converting total AF to MAF and estimating case and control MAFs. Converting first to MAF (scenario 3) introduces variability while using AF (scenario 1) or converting to MAF after estimation (scenario 2) does not introduce variability. Since CaseControl_SE requires converting AF to MAF prior to estimation, additional variability is likely introduced due to this step. Lin's CCC is reported between the true and estimated AF.

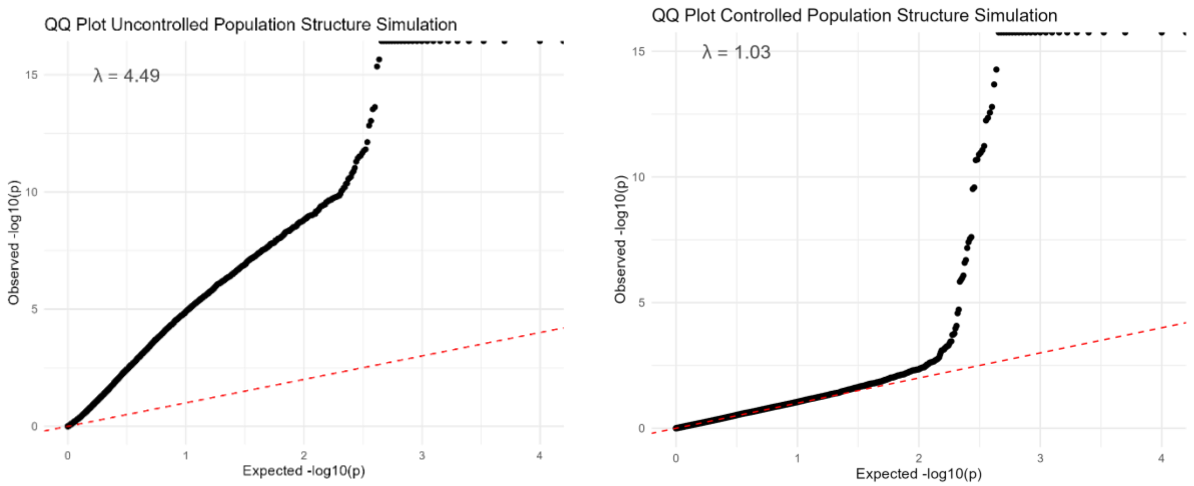

Figure S12. **Population stratification simulations correct genomic inflation.** QQ-plots for the 10,000 variants with simulated population structure without (left) and with (right) correction using principal components analysis. P-values below 10^-16 were rounded resulting in the cap seen here.

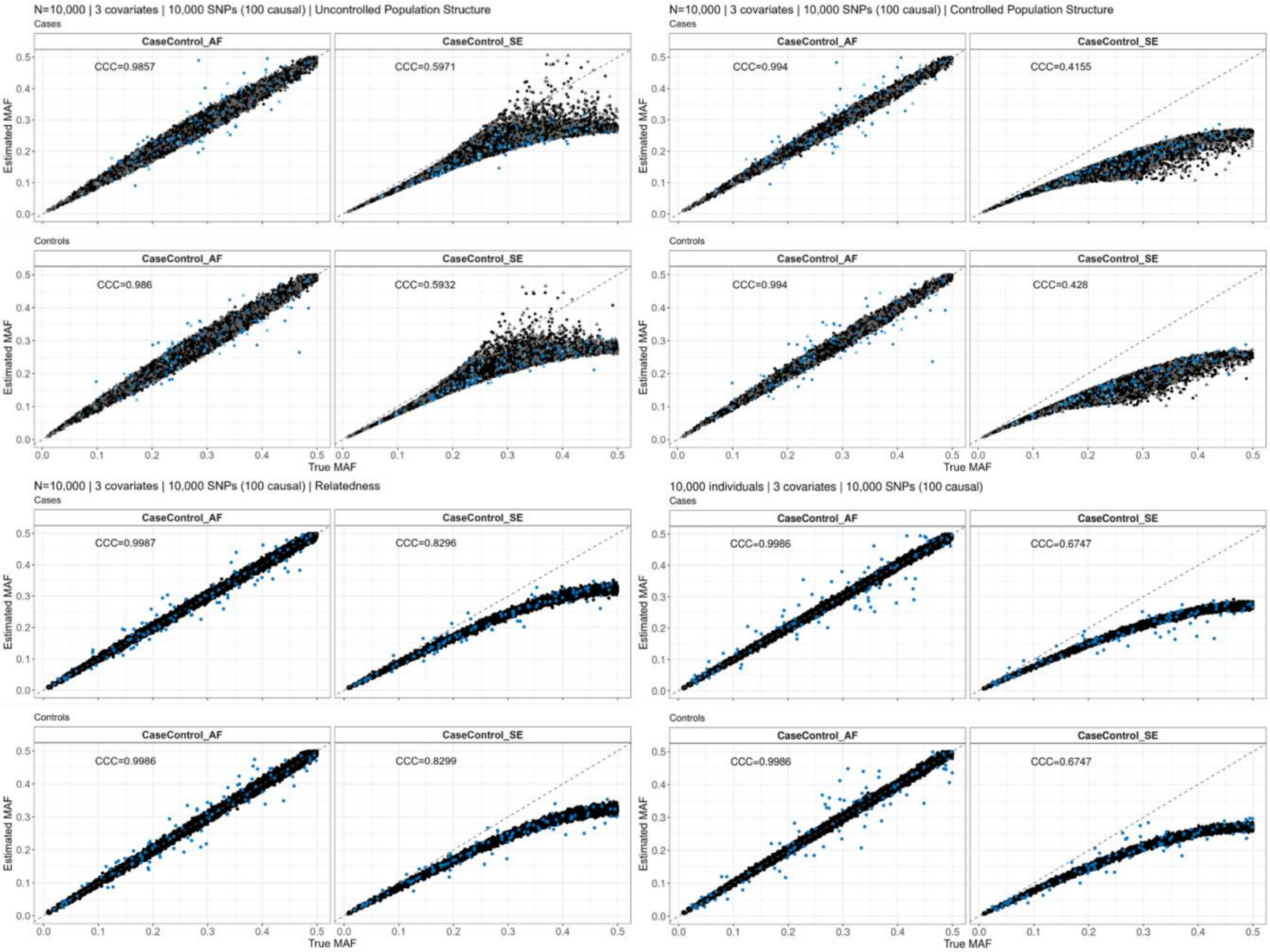

Figure S13. **Uncontrolled population stratification increases AF estimate variability, but not bias; relatedness has no noticeable effects.** Genotypes for 10,000 variants, of which 100 were causal (blue circles) and 9,900 were non-causal (black circles), were generated for 5,000 cases and 5,000 controls. For the **top panels**, 10% of variants (1,000) were simulated to be affected by population stratification (light blue and grey triangles for causal and non-causal respectively). Logistic regression was used along with 3 covariates to generate per variant summary statistics, without **(top left)** or with **(top right)** adjustment for population structure using 10 PCs. While adjusting for population structure resulted in lower variability in AF estimates for both CaseControl_AF and CaseControl_SE compared to not adjusting, AFs derived from GWAS summary statistics that had population stratification (both adjusted and unadjusted) had higher variability compared to summary statistics from GWAS without population structure **(bottom right)**. In the **bottom left** panel, relatedness was simulated to be 36% of the total phenotypic variance and did not have noticeable impact to the variance or bias of the AF estimates for either method.

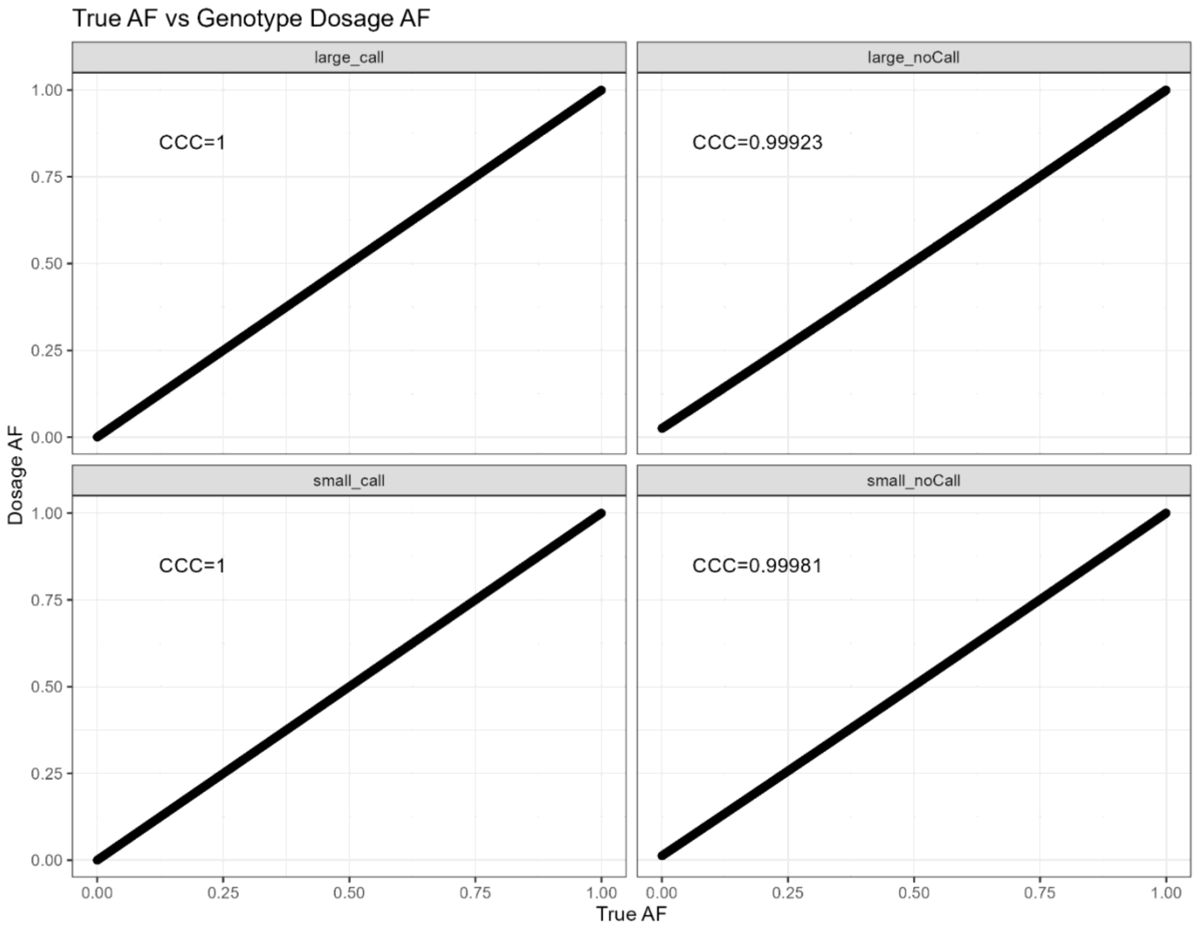

Figure S14. **Genotype dosage simulations.** Allele counts for bialleic variants (0,1,2) were simulated for 10,000 variants and 10,000 individuals. These counts were used to calculate exact allele frequency. Allele dosage with small (+/- 0.1) and large (+/-0.2) variability were simulated and used directly to calculate AF (right). Alleles were then called and used to calculate AF. These results were then compared to the exact AF (left). Lin's CCC was calculated comparing the exact simulated AF to the AFs calculated with added variability and with and without the allele calling.

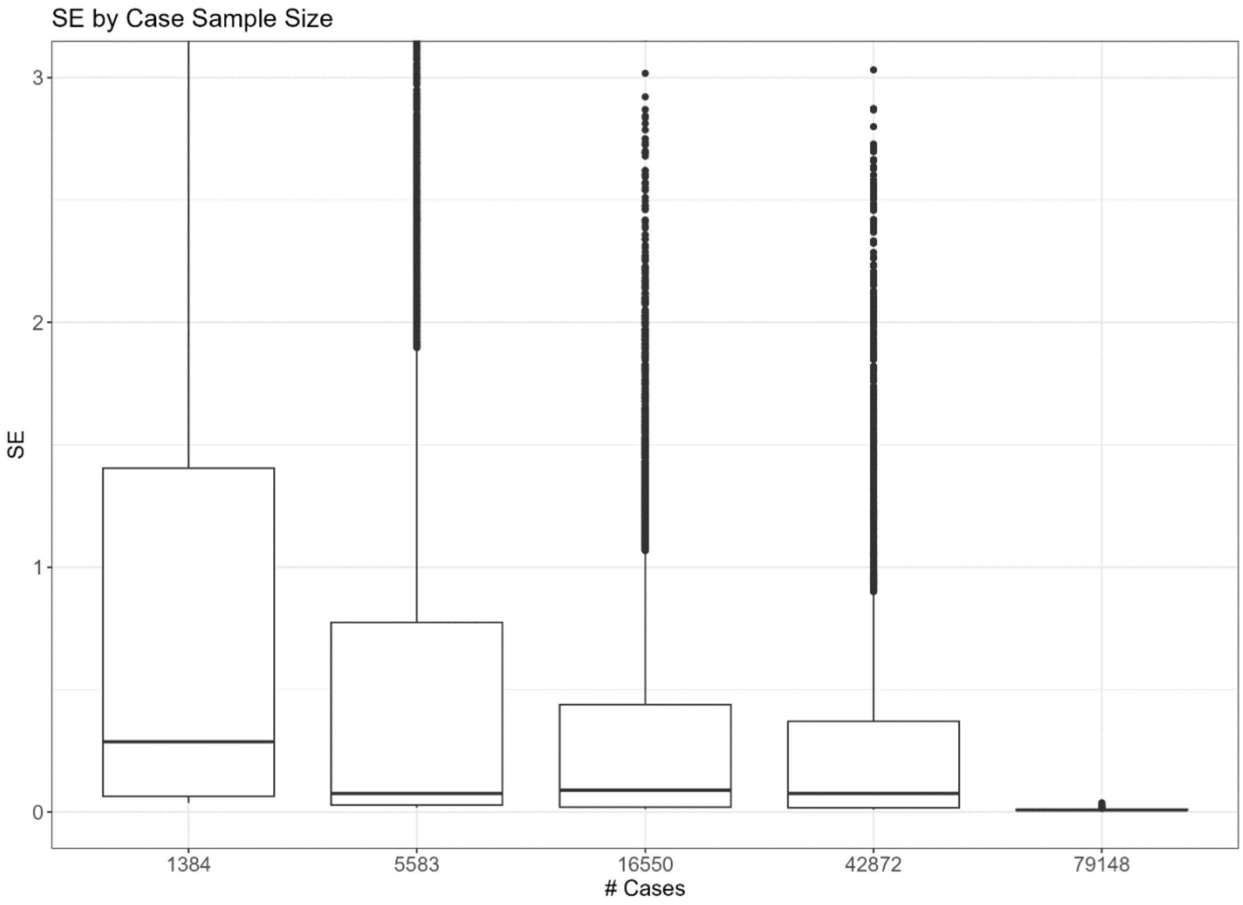

Figure S15. Larger sample sizes have smaller SE and less variability in SE. Here we examined the relationship between the case sample size (x-axis) and the standard error (y-axis). Studies with larger case sample sizes have a smaller SE including the minimum observed SE. Additionally, larger case sample sizes result in less variability in the SE across the variants. For each boxplot the center line shows the median and the upper and lower hinge represent the 75th and 25th percentile, respectively. The upper and lower whiskers are the largest and smallest values no more than 1.5* interquartile range (IQR) from the hinge.

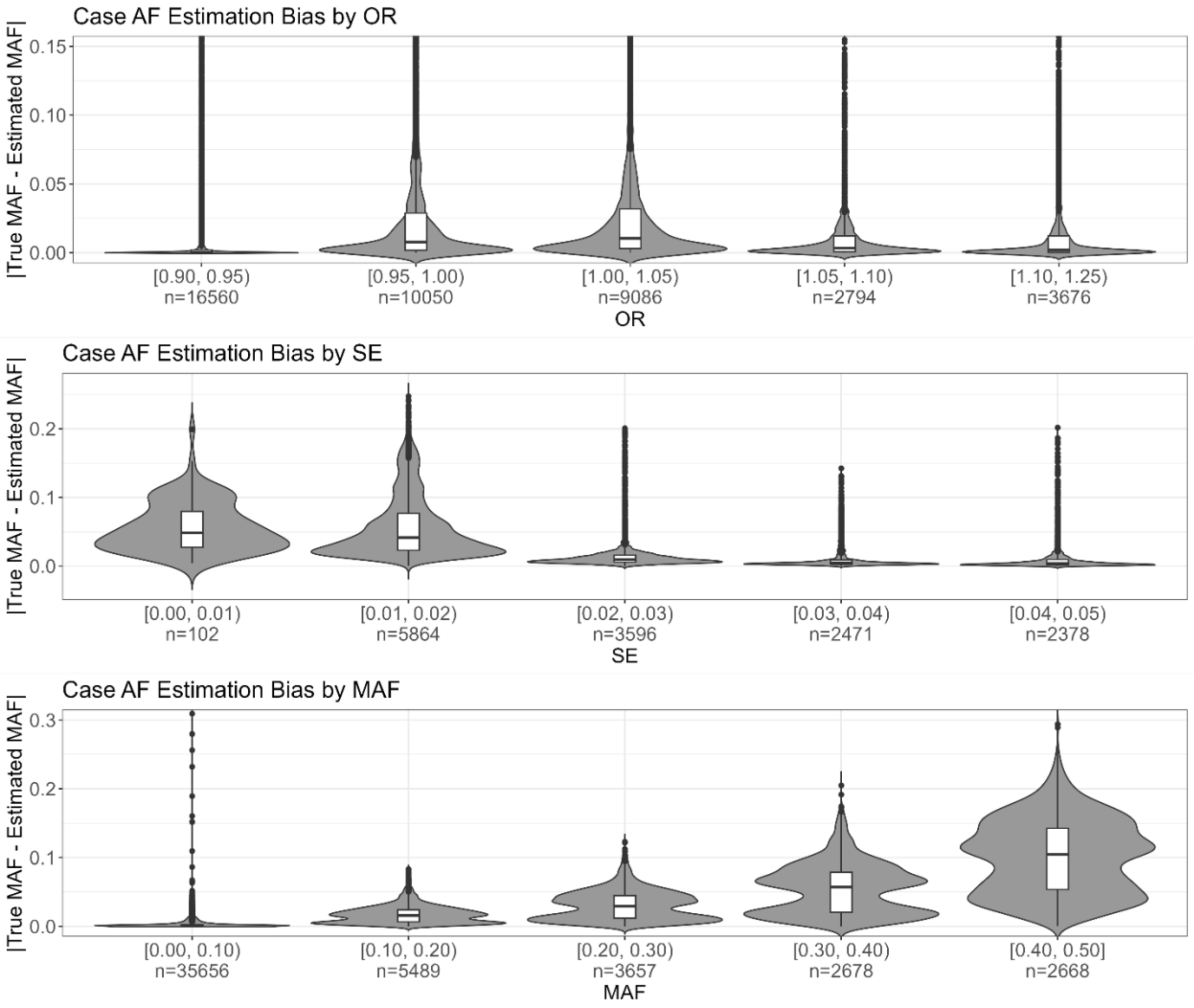

Figure S16. CaseControl_SE has high bias at large MAF and small SE. The difference between the true MAF and CaseControl_SE estimated MAF is compared across different bins for the OR (top), SE (middle), and MAF (bottom). Here the results from the 148 prostate cancer variants and 25,000 randomly sampled variants from Pan-UKBB diabetes in AFR and EUR are aggregated and plotted, with the number of SNPs in each bin shown on the x-axis. We see that bias is higher for smaller SE and larger MAF

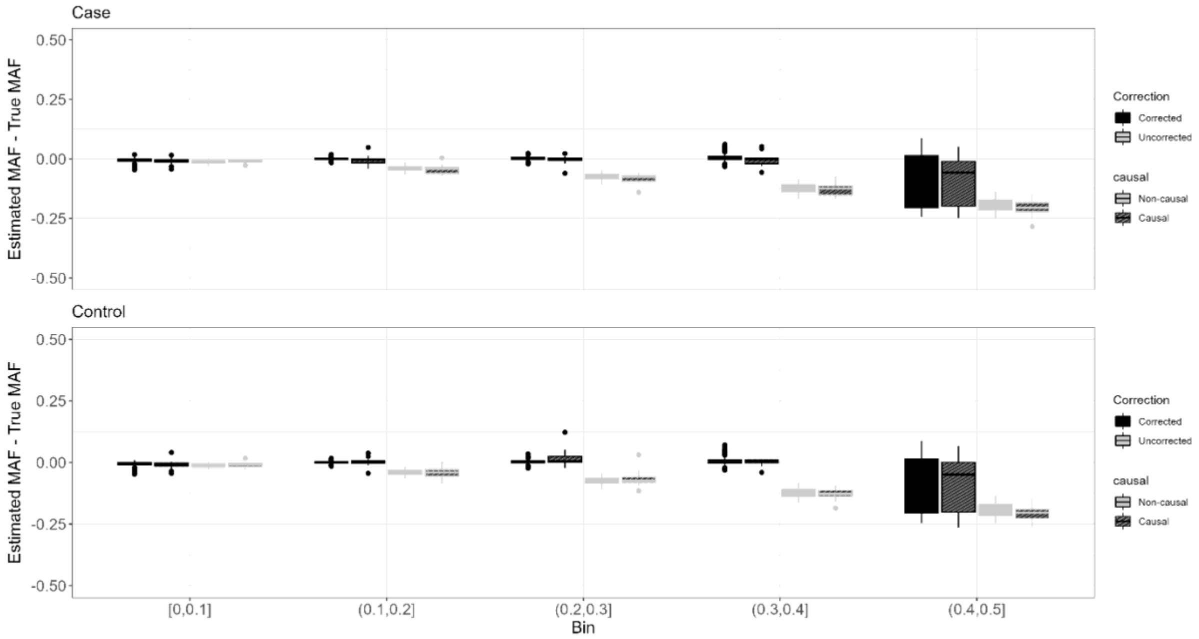

Figure S17. **Simulations show reduced bias using correction framework.** For the simulation of 10,000 variants (100 causal) for 10,000 individuals with 3 covariates, a proxy sample was also simulated to test the bias correction framework. The corrected estimates have much less bias than the uncorrected, but variability remains in the largest minor allele frequency bin. There is no notable difference in bias between the causal and non-causal variants before or after correction.

Table S1. Simulation scenarios to test root solutions

Parameter	Values
N_{case}	1000, 5000, 9000
$N_{control}$	9000, 5000, 1000
N_{total}	10000
AF_{total}	0, 0.1, 0.25, 0.5, 0.75, 1
OR	0.1, 0.4, 0.6, 0.8, 1.1, 1.2, 3, 5

Table S2. Simulations show only one possible root (root2) falls within valid interval [0,1] for control AF solution

OR	AF	N_case	N_control	N_total	a	b	c	root1	root2	sim
0.1	0	5000	5000	10000	-0.9000	1.1000	0.0000	1.2222	0.0000	1
0.4	0	5000	5000	10000	-0.6000	1.4000	0.0000	2.3333	0.0000	2
0.6	0	5000	5000	10000	-0.4000	1.6000	0.0000	4.0000	0.0000	3
0.8	0	5000	5000	10000	-0.2000	1.8000	0.0000	9.0000	0.0000	4
0.1	0	9000	1000	10000	-0.1000	0.2111	0.0000	2.1111	0.0000	5
0.4	0	9000	1000	10000	-0.0667	0.5111	0.0000	7.6667	0.0000	6
0.6	0	9000	1000	10000	-0.0444	0.7111	0.0000	16.0000	0.0000	7
0.8	0	9000	1000	10000	-0.0222	0.9111	0.0000	41.0000	0.0000	8
0.1	0	1000	9000	10000	-8.1000	9.1000	0.0000	1.1235	0.0000	9
0.4	0	1000	9000	10000	-5.4000	9.4000	0.0000	1.7407	0.0000	10
0.6	0	1000	9000	10000	-3.6000	9.6000	0.0000	2.6667	0.0000	11
0.8	0	1000	9000	10000	-1.8000	9.8000	0.0000	5.4444	0.0000	12
0.1	0.1	5000	5000	10000	-0.9000	1.2800	-0.2000	1.2435	0.1787	13
0.4	0.1	5000	5000	10000	-0.6000	1.5200	-0.2000	2.3941	0.1392	14
0.6	0.1	5000	5000	10000	-0.4000	1.6800	-0.2000	4.0774	0.1226	15
0.8	0.1	5000	5000	10000	-0.2000	1.8400	-0.2000	9.0900	0.1100	16
0.1	0.1	9000	1000	10000	-0.1000	0.3111	-0.1111	2.6995	0.4116	17
0.4	0.1	9000	1000	10000	-0.0667	0.5778	-0.1111	8.4699	0.1968	18
0.6	0.1	9000	1000	10000	-0.0444	0.7556	-0.1111	16.8516	0.1484	19
0.8	0.1	9000	1000	10000	-0.0222	0.9333	-0.1111	41.8806	0.1194	20
0.1	0.1	1000	9000	10000	-8.1000	10.0000	-1.0000	1.1248	0.1098	21
0.4	0.1	1000	9000	10000	-5.4000	10.0000	-1.0000	1.7458	0.1061	22
0.6	0.1	1000	9000	10000	-3.6000	10.0000	-1.0000	2.6739	0.1039	23
0.8	0.1	1000	9000	10000	-1.8000	10.0000	-1.0000	5.4537	0.1019	24
0.1	0.25	5000	5000	10000	-0.9000	1.5500	-0.5000	1.2923	0.4299	25
0.4	0.25	5000	5000	10000	-0.6000	1.7000	-0.5000	2.5000	0.3333	26
0.6	0.25	5000	5000	10000	-0.4000	1.8000	-0.5000	4.2026	0.2974	27
0.8	0.25	5000	5000	10000	-0.2000	1.9000	-0.5000	9.2291	0.2709	28
0.1	0.25	9000	1000	10000	-0.1000	0.4611	-0.2778	3.8986	0.7125	29
0.4	0.25	9000	1000	10000	-0.0667	0.6778	-0.2778	9.7388	0.4278	30
0.6	0.25	9000	1000	10000	-0.0444	0.8222	-0.2778	18.1558	0.3442	31
0.8	0.25	9000	1000	10000	-0.0222	0.9667	-0.2778	43.2107	0.2893	32
0.1	0.25	1000	9000	10000	-8.1000	11.3500	-2.5000	1.1275	0.2737	33
0.4	0.25	1000	9000	10000	-5.4000	10.9000	-2.5000	1.7547	0.2638	34
0.6	0.25	1000	9000	10000	-3.6000	10.6000	-2.5000	2.6859	0.2586	35
0.8	0.25	1000	9000	10000	-1.8000	10.3000	-2.5000	5.4682	0.2540	36
0.1	0.5	5000	5000	10000	-0.9000	2.0000	-1.0000	1.4625	0.7597	37
0.4	0.5	5000	5000	10000	-0.6000	2.0000	-1.0000	2.7208	0.6126	38
0.6	0.5	5000	5000	10000	-0.4000	2.0000	-1.0000	4.4365	0.5635	39
0.8	0.5	5000	5000	10000	-0.2000	2.0000	-1.0000	9.4721	0.5279	40
0.1	0.5	9000	1000	10000	-0.1000	0.7111	-0.5556	6.2176	0.8935	41

0.4	0.5	9000	1000	10000	-0.0667	0.8444	-0.5556	11.9705	0.6962	42
0.6	0.5	9000	1000	10000	-0.0444	0.9333	-0.5556	20.3869	0.6131	43
0.8	0.5	9000	1000	10000	-0.0222	1.0222	-0.5556	45.4499	0.5501	44
0.1	0.5	1000	9000	10000	-8.1000	13.6000	-5.0000	1.1353	0.5437	45
0.4	0.5	1000	9000	10000	-5.4000	12.4000	-5.0000	1.7745	0.5218	46
0.6	0.5	1000	9000	10000	-3.6000	11.6000	-5.0000	2.7097	0.5126	47
0.8	0.5	1000	9000	10000	-1.8000	10.8000	-5.0000	5.4944	0.5056	48
0.1	0.75	5000	5000	10000	-0.9000	2.4500	-1.5000	1.7923	0.9299	49
0.4	0.75	5000	5000	10000	-0.6000	2.3000	-1.5000	3.0000	0.8333	50
0.6	0.75	5000	5000	10000	-0.4000	2.2000	-1.5000	4.7026	0.7974	51
0.8	0.75	5000	5000	10000	-0.2000	2.1000	-1.5000	9.7291	0.7709	52
0.1	0.75	9000	1000	10000	-0.1000	0.9611	-0.8333	8.6474	0.9637	53
0.4	0.75	9000	1000	10000	-0.0667	1.0111	-0.8333	14.2921	0.8746	54
0.6	0.75	9000	1000	10000	-0.0444	1.0444	-0.8333	22.6730	0.8270	55
0.8	0.75	9000	1000	10000	-0.0222	1.0778	-0.8333	47.7141	0.7859	56
0.1	0.75	1000	9000	10000	-8.1000	15.8500	-7.5000	1.1554	0.8014	57
0.4	0.75	1000	9000	10000	-5.4000	13.9000	-7.5000	1.8043	0.7698	58
0.6	0.75	1000	9000	10000	-3.6000	12.6000	-7.5000	2.7395	0.7605	59
0.8	0.75	1000	9000	10000	-1.8000	11.3000	-7.5000	5.5234	0.7544	60
0.1	1	5000	5000	10000	-0.9000	2.9000	-2.0000	2.2222	1.0000	61
0.4	1	5000	5000	10000	-0.6000	2.6000	-2.0000	3.3333	1.0000	62
0.6	1	5000	5000	10000	-0.4000	2.4000	-2.0000	5.0000	1.0000	63
0.8	1	5000	5000	10000	-0.2000	2.2000	-2.0000	10.0000	1.0000	64
0.1	1	9000	1000	10000	-0.1000	1.2111	-1.1111	11.1111	1.0000	65
0.4	1	9000	1000	10000	-0.0667	1.1778	-1.1111	16.6667	1.0000	66
0.6	1	9000	1000	10000	-0.0444	1.1556	-1.1111	25.0000	1.0000	67
0.8	1	9000	1000	10000	-0.0222	1.1333	-1.1111	50.0000	1.0000	68
0.1	1	1000	9000	10000	-8.1000	18.1000	-10.0000	1.2346	1.0000	69
0.4	1	1000	9000	10000	-5.4000	15.4000	-10.0000	1.8519	1.0000	70
0.6	1	1000	9000	10000	-3.6000	13.6000	-10.0000	2.7778	1.0000	71
0.8	1	1000	9000	10000	-1.8000	11.8000	-10.0000	5.5556	1.0000	72
1.1	0	5000	5000	10000	0.1000	2.1000	0.0000	-21.0000	0.0000	73
1.2	0	5000	5000	10000	0.2000	2.2000	0.0000	-11.0000	0.0000	74
3	0	5000	5000	10000	2.0000	4.0000	0.0000	-2.0000	0.0000	75
5	0	5000	5000	10000	4.0000	6.0000	0.0000	-1.5000	0.0000	76
1.1	0	9000	1000	10000	0.0111	1.2111	0.0000	-109.0000	0.0000	77
1.2	0	9000	1000	10000	0.0222	1.3111	0.0000	-59.0000	0.0000	78
3	0	9000	1000	10000	0.2222	3.1111	0.0000	-14.0000	0.0000	79
5	0	9000	1000	10000	0.4444	5.1111	0.0000	-11.5000	0.0000	80
1.1	0	1000	9000	10000	0.9000	10.1000	0.0000	-11.2222	0.0000	81
1.2	0	1000	9000	10000	1.8000	10.2000	0.0000	-5.6667	0.0000	82
3	0	1000	9000	10000	18.0000	12.0000	0.0000	-0.6667	0.0000	83
5	0	1000	9000	10000	36.0000	14.0000	0.0000	-0.3889	0.0000	84

1.1	0.1	5000	5000	10000	0.1000	2.0800	-0.2000	-20.8957	0.0957	85
1.2	0.1	5000	5000	10000	0.2000	2.1600	-0.2000	-10.8918	0.0918	86
3	0.1	5000	5000	10000	2.0000	3.6000	-0.2000	-1.8539	0.0539	87
5	0.1	5000	5000	10000	4.0000	5.2000	-0.2000	-1.3374	0.0374	88
1.1	0.1	9000	1000	10000	0.0111	1.2000	-0.1111	-108.0925	0.0925	89
1.2	0.1	9000	1000	10000	0.0222	1.2889	-0.1111	-58.0861	0.0861	90
3	0.1	9000	1000	10000	0.2222	2.8889	-0.1111	-13.0383	0.0383	91
5	0.1	9000	1000	10000	0.4444	4.6667	-0.1111	-10.5238	0.0238	92
1.1	0.1	1000	9000	10000	0.9000	10.0000	-1.0000	-11.2102	0.0991	93
1.2	0.1	1000	9000	10000	1.8000	10.0000	-1.0000	-5.6538	0.0983	94
3	0.1	1000	9000	10000	18.0000	10.0000	-1.0000	-0.6421	0.0865	95
5	0.1	1000	9000	10000	36.0000	10.0000	-1.0000	-0.3558	0.0781	96
1.1	0.25	5000	5000	10000	0.1000	2.0500	-0.5000	-20.7411	0.2411	97
1.2	0.25	5000	5000	10000	0.2000	2.1000	-0.5000	-10.7329	0.2329	98
3	0.25	5000	5000	10000	2.0000	3.0000	-0.5000	-1.6514	0.1514	99
5	0.25	5000	5000	10000	4.0000	4.0000	-0.5000	-1.1124	0.1124	100
1.1	0.25	9000	1000	10000	0.0111	1.1833	-0.2778	-106.7342	0.2342	101
1.2	0.25	9000	1000	10000	0.0222	1.2556	-0.2778	-56.7204	0.2204	102
3	0.25	9000	1000	10000	0.2222	2.5556	-0.2778	-11.6077	0.1077	103
5	0.25	9000	1000	10000	0.4444	4.0000	-0.2778	-9.0689	0.0689	104
1.1	0.25	1000	9000	10000	0.9000	9.8500	-2.5000	-11.1926	0.2482	105
1.2	0.25	1000	9000	10000	1.8000	9.7000	-2.5000	-5.6353	0.2465	106
3	0.25	1000	9000	10000	18.0000	7.0000	-2.5000	-0.6148	0.2259	107
5	0.25	1000	9000	10000	36.0000	4.0000	-2.5000	-0.3249	0.2138	108
1.1	0.5	5000	5000	10000	0.1000	2.0000	-1.0000	-20.4881	0.4881	109
1.2	0.5	5000	5000	10000	0.2000	2.0000	-1.0000	-10.4772	0.4772	110
3	0.5	5000	5000	10000	2.0000	2.0000	-1.0000	-1.3660	0.3660	111
5	0.5	5000	5000	10000	4.0000	2.0000	-1.0000	-0.8090	0.3090	112
1.1	0.5	9000	1000	10000	0.0111	1.1556	-0.5556	-104.4786	0.4786	113
1.2	0.5	9000	1000	10000	0.0222	1.2000	-0.5556	-54.4591	0.4591	114
3	0.5	9000	1000	10000	0.2222	2.0000	-0.5556	-9.2697	0.2697	115
5	0.5	9000	1000	10000	0.4444	2.8889	-0.5556	-6.6869	0.1869	116
1.1	0.5	1000	9000	10000	0.9000	9.6000	-5.0000	-11.1643	0.4976	117
1.2	0.5	1000	9000	10000	1.8000	9.2000	-5.0000	-5.6066	0.4955	118
3	0.5	1000	9000	10000	18.0000	2.0000	-5.0000	-0.5855	0.4744	119
5	0.5	1000	9000	10000	36.0000	-6.0000	-5.0000	-0.2985	0.4652	120
1.1	0.75	5000	5000	10000	0.1000	1.9500	-1.5000	-20.2411	0.7411	121
1.2	0.75	5000	5000	10000	0.2000	1.9000	-1.5000	-10.2329	0.7329	122
3	0.75	5000	5000	10000	2.0000	1.0000	-1.5000	-1.1514	0.6514	123
5	0.75	5000	5000	10000	4.0000	0.0000	-1.5000	-0.6124	0.6124	124
1.1	0.75	9000	1000	10000	0.0111	1.1278	-0.8333	-102.2336	0.7336	125
1.2	0.75	9000	1000	10000	0.0222	1.1444	-0.8333	-52.2181	0.7181	126
3	0.75	9000	1000	10000	0.2222	1.4444	-0.8333	-7.0332	0.5332	127

5	0.75	9000	1000	10000	0.4444	1.7778	-0.8333	-4.4238	0.4238	128
1.1	0.75	1000	9000	10000	0.9000	9.3500	-7.5000	-11.1371	0.7482	129
1.2	0.75	1000	9000	10000	1.8000	8.7000	-7.5000	-5.5800	0.7467	130
3	0.75	1000	9000	10000	18.0000	-3.0000	-7.5000	-0.5675	0.7342	131
5	0.75	1000	9000	10000	36.0000	-16.0000	-7.5000	-0.2854	0.7299	132
1.1	1	5000	5000	10000	0.1000	1.9000	-2.0000	-20.0000	1.0000	133
1.2	1	5000	5000	10000	0.2000	1.8000	-2.0000	-10.0000	1.0000	134
3	1	5000	5000	10000	2.0000	0.0000	-2.0000	-1.0000	1.0000	135
5	1	5000	5000	10000	4.0000	-2.0000	-2.0000	-0.5000	1.0000	136
1.1	1	9000	1000	10000	0.0111	1.1000	-1.1111	-100.0000	1.0000	137
1.2	1	9000	1000	10000	0.0222	1.0889	-1.1111	-50.0000	1.0000	138
3	1	9000	1000	10000	0.2222	0.8889	-1.1111	-5.0000	1.0000	139
5	1	9000	1000	10000	0.4444	0.6667	-1.1111	-2.5000	1.0000	140
1.1	1	1000	9000	10000	0.9000	9.1000	-10.0000	-11.1111	1.0000	141
1.2	1	1000	9000	10000	1.8000	8.2000	-10.0000	-5.5556	1.0000	142
3	1	1000	9000	10000	18.0000	-8.0000	-10.0000	-0.5556	1.0000	143
5	1	1000	9000	10000	36.0000	-26.0000	-10.0000	-0.2778	1.0000	144

Table S3. Control sample results for Lin's Concordance Correlation Coefficient for CaseControl_AF, CaseControl_SE, and bias corrected CaseControl_SE between true and estimated MAF

					Lin's CCC		
**Trait**	**Cases**	**Controls**	**Bin**	**N Variants**	**CCAFE AF**	**CCAFE SE**	**CCAFE SE Corrected**
Prostate Cancer	79148	61106	[0.0, 0.1]	18	1	0.90315	NA*
			(0.1, 0.2]	27	1	0.73818	NA
			(0.2, 0.3]	35	1	0.50244	NA
			(0.3, 0.4]	32	1	0.24222	NA
			(0.4, 0.5]	36	1	0.03586	NA
PanUKBB Diabetes	16550	403923	[0.0, 0.1]	3721679	1	0.9700	0.9651
			(0.1, 0.2]	1766962	1	0.7338	0.9802
			(0.2, 0.3]	1355742	1	0.3972	0.9389
			(0.3, 0.4]	1203911	1	0.1428	0.8214
			(0.4, 0.5]	1130270	1	0.0213	0.2060
PanUKBB Diabetes	668	5956	[0.0, 0.1]	3453818	0.99998	0.9567	0.9760
			(0.1, 0.2]	1871888	0.99995	0.7097	0.8850
			(0.2, 0.3]	1487374	0.99994	0.4129	0.7449
			(0.3, 0.4]	1242683	0.99993	0.1651	0.4508
			(0.4, 0.5]	1126168	0.99992	0.0281	0.2175

* Prostate cancer MAFs were not corrected due to small number of overlapping variants with gnomAD

Table S4. Effect of rounding for CaseControl_SE. Lin's Concordance Correlation Coefficient (CCC) for known vs estimated case and control AFs.

Case	Number of Decimal Places Rounded			
bin	5	4	3	2
[0,0.1]	0.9843	0.9842	0.9842	0.9711
(0.1,0.2]	0.7332	0.7329	0.7359	0.1376
(0.2,0.3]	0.3946	0.3936	0.4316	0.0134
(0.3,0.4]	0.1377	0.1383	0.2317	0.0000
(0.4,0.5]	0.0215	0.0214	0.0026	0.0000
**Control**	**Number of Decimal Places Rounded**			
**bin**	**5**	**4**	**3**	**2**
[0,0.1]	0.9803	0.9803	0.9803	0.9677
(0.1,0.2]	0.7347	0.7344	0.7375	0.1386
(0.2,0.3]	0.3964	0.3954	0.4332	0.0136
(0.3,0.4]	0.1407	0.1413	0.2350	0.0007
(0.4,0.5]	0.0216	0.0214	0.0027	0.0008

Table S5. Effect of rounding for CaseControl_AF. Lin's Concordance Correlation Coefficient (CCC) for known vs estimated case and control AFs.

Case	Number of Decimal Places Rounded			
bin	5	4	3	2
[0,0.1]	1.0000	1.0000	0.9999	0.9970
(0.1,0.2]	0.9999	0.9999	0.9999	0.9951
(0.2,0.3]	0.9999	0.9999	0.9998	0.9950
(0.3,0.4]	0.9999	0.9999	0.9998	0.9950
(0.4,0.5]	0.9999	0.9999	0.9998	0.9950
**Control**	**Number of Decimal Places Rounded**			
**bin**	**5**	**4**	**3**	**2**
[0,0.1]	1.0000	1.0000	1.0000	0.9971
(0.1,0.2]	1.0000	1.0000	1.0000	0.9952
(0.2,0.3]	1.0000	1.0000	1.0000	0.9951
(0.3,0.4]	1.0000	1.0000	1.0000	0.9952
(0.4,0.5]	1.0000	1.0000	1.0000	0.9951

* rounding to 6-9 decimal places was the same as five
